# Whole-genome resequencing provides insights into the population structure and domestication signatures of ducks in eastern China

**DOI:** 10.1186/s12864-021-07710-2

**Published:** 2021-05-31

**Authors:** Peishi Feng, Tao Zeng, Hua Yang, Guohong Chen, Jinping Du, Li Chen, Junda Shen, Zhenrong Tao, Ping Wang, Lin Yang, Lizhi Lu

**Affiliations:** 1 College of Pharmaceutical Science, Zhejiang University of Technology, Hangzhou, China; 2 Institute of Animal Husbandry and Veterinary Medicine, Zhejiang Academy of Agricultural Sciences, Hangzhou, China; 3 Institute of Quality and Standards for Agro-products, Zhejiang Academy of Agricultural Sciences, Hangzhou, China; 4 College of Animal Science and Technology, Yangzhou University, Yangzhou, China; 5 Institute of Animal Husbandry and Veterinary Science, Hubei Academy of Agricultural Science, Wuhan, China; 6 College of Animal Science, South China Agricultural University, Guangzhou, China

**Keywords:** Duck, Domestication, Insulin signaling pathway, Population history, Artificial selection, Adaptation

## Abstract

**Background:**

Duck is an ancient domesticated animal with high economic value, used for its meat, eggs, and feathers. However, the origin of indigenous Chinese ducks remains elusive. To address this question, we performed whole-genome resequencing to first explore the genetic relationship among variants of these domestic ducks with their potential wild ancestors in eastern China, as well as understand how the their genomes were shaped by different natural and artificial selective pressures.

**Results:**

Here, we report the resequencing of 60 ducks from Chinese spot-billed ducks (*Anas zonorhyncha)*, mallards *(Anas platyrhnchos*), Fenghua ducks, Shaoxing ducks, Shanma ducks and Cherry Valley Pekin ducks of eastern China (ten from each population) at an average effective sequencing depth of ~ 6× per individual. The results of population and demographic analysis revealed a deep phylogenetic split between wild (Chinese spot-billed ducks and mallards) and domestic ducks. By applying selective sweep analysis, we identified that several candidate genes, important pathways and GO categories associated with artificial selection were functionally related to cellular adhesion, type 2 diabetes, lipid metabolism, the cell cycle, liver cell proliferation, and muscle functioning in domestic ducks.

**Conclusion:**

Genetic structure analysis showed a close genetic relationship of Chinese spot-billed ducks and mallards, which supported that Chinese spot-billed ducks contributed to the breeding of domestic ducks. During the long history of artificial selection, domestic ducks have developed a complex biological adaptation to captivity.

**Supplementary Information:**

The online version contains supplementary material available at 10.1186/s12864-021-07710-2.

## Background

Domestication is the process of animal adaptation to captive environment and human interventions such as providing protection, offering food and promoting animal breeding [1]. Compared to their wild ancestors, domestic animals have great variation in behavior, morphology and physiology in response to domestication, and this variation is the result of genetic changes across many generations. The genetic differentiation among domestic animals and their wild ancestors is influenced by multiple mechanisms, including selection, mutation, drift and gene flow [2]. Detecting selective signatures associated with domestication is important for understanding the genetic basis of both adaptations to new environments and rapid phenotype change. In recent years, whole-genome resequencing delivers a comprehensive view of detecting the signatures left by domestication, such as in pig [3], chickens [4], dogs [5] and yaks [6].

Chinese domestic ducks are among the earliest domesticated waterfowl in the world dating back to 2228 years before present (YBP) [7]. China is famous for its abundance of waterfowl breeds, as many as 31 domestic duck breeds have been recognized. Owing to domestication and directional breeding, domestic ducks have many typical characteristics in morphology, behavior and production performance, such as reduction in brain size [8], leg morphology changes [9], decrease aggression behaviors [10] and higher egg productivity. Domestic ducks have been bred for various purposes, such as egg and/or meat production. Shaoxing and Shanma ducks are Chinese excellent egg-type duck breeds, characterized by small body size, early maturity and high productivity. In Chinese written history, Shaoxing duck can be traced back to the Song Dynasty about 1000 years ago. Through 50 years of systematic breeding, the egg production of Shaoxing ducks reached 300 at the age of 500 days [11]. Shanma duck, another famous Chinese indigenous duck, has been domesticated for 400 years in Fujian Province [12]. Fenghua (FH) duck is a special dual-purpose local duck breed in Zhejiang Province, which has similar appearance with mallards. Different from other domestic breeds, Fenghua duck still retains some habits of wild ducks such as seasonal reproduction, flying and high disease resistance, because of the short time of domestication. Chinese Pekin ducks are named Cherry Valley Pekin ducks after they were exported to the United Kingdom in1872. After more than 100 years of intensive selection, Cherry Valley Pekin ducks are famous for their fast-growth, high lean rate and high feed conversion ratio [13].

Although many studies have been conducted on the diversity and origin of Chinese domestic ducks by applying microsatellite markers, mitochondrial DNA sequencing and whole-genome resequencing, the origin and evolution of Chinese domestic ducks are still debated. Some scholars suggest that Chinese domestic ducks originated from wild mallards [14, 15], while others argue that domestic ducks might also originate from Chinese spot-billed ducks [16, 17]. Mallard is the most common wild duck species in China, which is of particular economic importance [18]. Chinese spot-billed duck is a close relative of mallard, with distributions partially overlapping in most of Japan, Korea, and northeastern China [19]. Owing to the observed hybridization of mallards and spot-billed ducks in East Asia [19], another hypothesis suggests that domestic ducks might originate from hybrids of mallards and spot-billed ducks [17, 20].

Ducks are not only economically import, but serve as important non-model study systems in evolutionary biology [21]. Thus, elucidating the evolutionary history of the various domestic breeds is essential when attempting to understand how different selective regimes have shaped their genetic variation. Therefore, we sequenced the genomes of 60 individuals from two wild populations, the spot-billed ducks and mallards, and four indigenous Chinese breeds (Fenghua, Shaoxing, Shanma and Cherry Valley Pekin ducks) to explore the genetic relationships among wild and domestic ducks and identify the genomic footprints of selection during the domestication of native ducks.

## Results

**Fig. 1:**
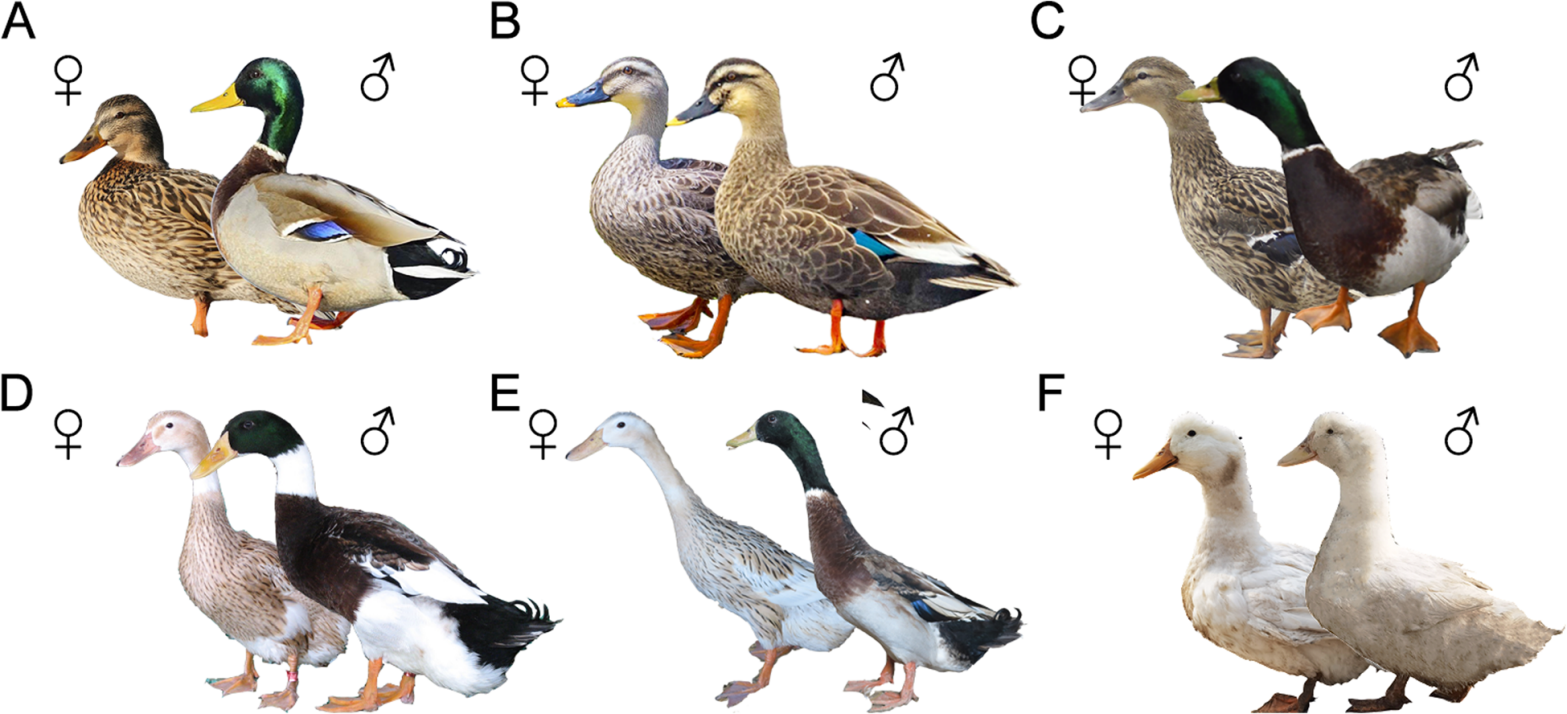
Graphical representation of six duck populations. **a** Mallard (**b**) Chinese Spot-billed duck (**c**) Fenghua duck (**d**) Shaoxing duck (**e**) Shanma duck (**f**) Cherry Valley Pekin duck

We selected 60 individuals from six breeds (mallard, Chinese spot-billed, Fenghua, Shaoxing, Shanma and Cherry Valley Pekin ducks) (Fig. 1 and Supplementary Table S1). Using the Illumina Genome Analyzer platform, we generated a total of 397.88 GB of clean data with an average of 6.63 GB per individuals (Supplementary Table S2). 2.5 billion reads mapped to 95.09% of the reference genome assembly with 6.52-fold average depth (Supplementary Table S3). We called 2,809,077 high-quality single nucleotide polymorphic sites (SNPs) for 60 ducks, 63.92% (1.8 million) of the high-quality SNPs were located in the intergenic regions, and only 1.94% (0.55 million) were located in the exonic regions (Supplementary Table S4–5). We identified 42,463 synonymous SNPs and 12,084 nonsynonymous of exons, for a nonsynonymous/synonymous ratio of 0.28. And 838,413 SNPs were common between six breeds (Supplementary Fig. S1).

### Population genetic structure

**Fig. 2:**
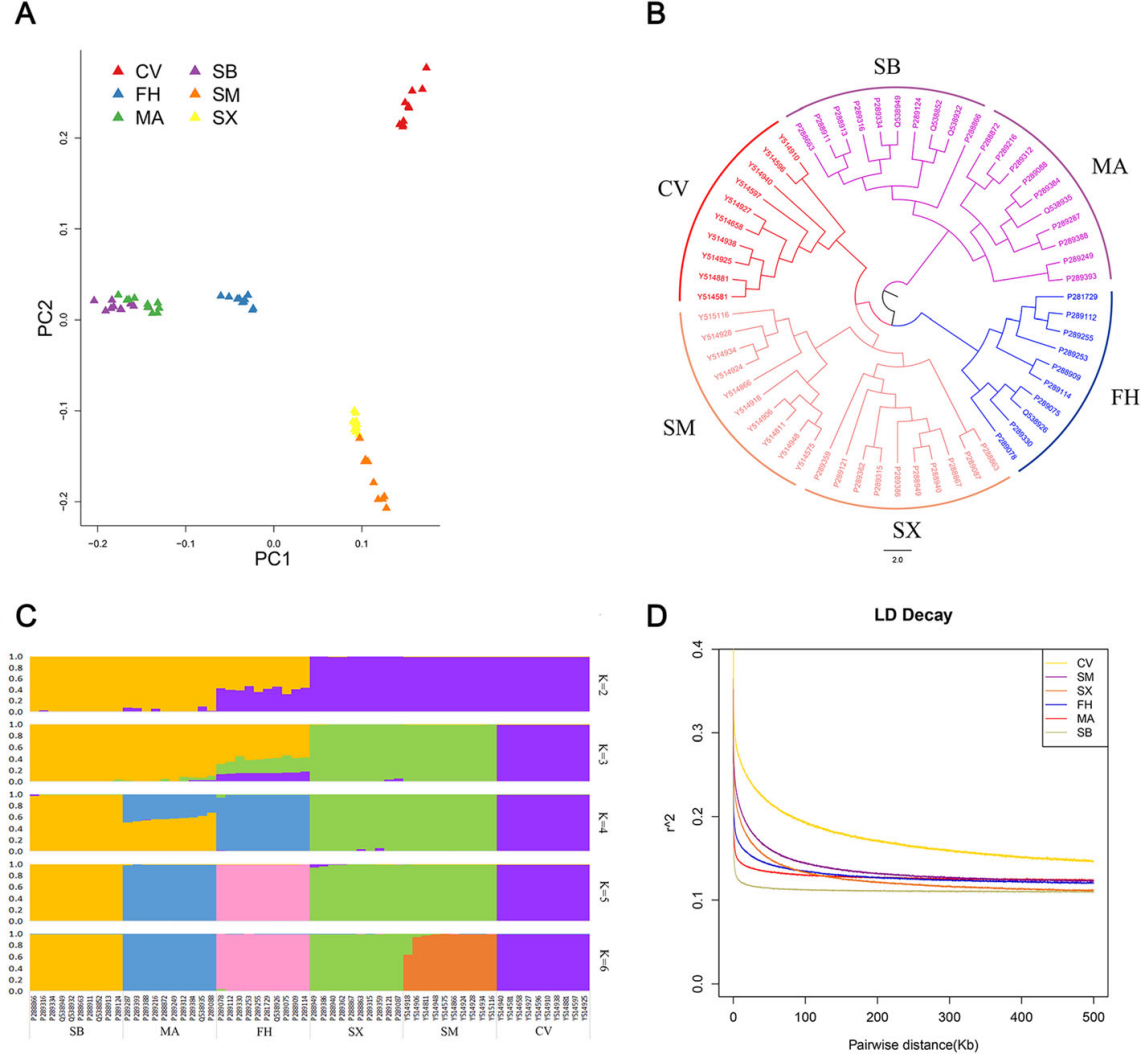
Phylogenetic and population genetic analyses of wild and domestic ducks. MA, mallards; SB, Chinese spot-billed ducks; FH, Fenghua ducks; SX, Shaoxing ducks; SM, Shanma ducks; CV, Cherry Valley Pekin ducks. **a** Principal component plot of 60 individuals. **b** Unrooted neighbor-joining tree constructed using the *p*-distances between individuals. **c** Population structure of 60 ducks (*K* = 2–6). The *y*-axis represents the proportion of the individual’s genome from inferred ancestral populations, and *x*-axis represents the different populations. **d** Genome-wide linkage disequilibrium of ducks

To explore relatedness among the domestic ducks, we conducted a principal component analysis (PCA) based on genome wide SNP data. The laying duck breeds (Shaoxing and Shanma ducks) and meat duck breeds (Cherry Valley Pekin duck) were separated by different clusters that were also distinct from the wild populations (Chinese spot-billed duck and mallard) and Fenghua duck (Fig. 2a, supplementary Fig. S2). The neighbor-joining (NJ) tree revealed that the individuals from Chinese indigenous breeds were clustered into a subclade, suggesting they have a closer genetic relationship and potentially derive from a common ancestor (Fig. 2b). To estimate different ancestral proportions, we further performed a population structure analysis with FRAPPE by assuming *K* ancestral populations (Fig. 2c). When *K* = 2, a clear division was observer between wild and domestic ducks with slight shared ancestry between these two groups. Moreover, Fenghua ducks appeared admixed, with individuals having on average of 59 and 41% assignment probability to wild and domestic breeds, respectively; suggesting these represent a wild × domestic duck hybrid population. When *K* = 5, there was a division between each group except Shaoxing and Shanma ducks.

**Fig. 3:**
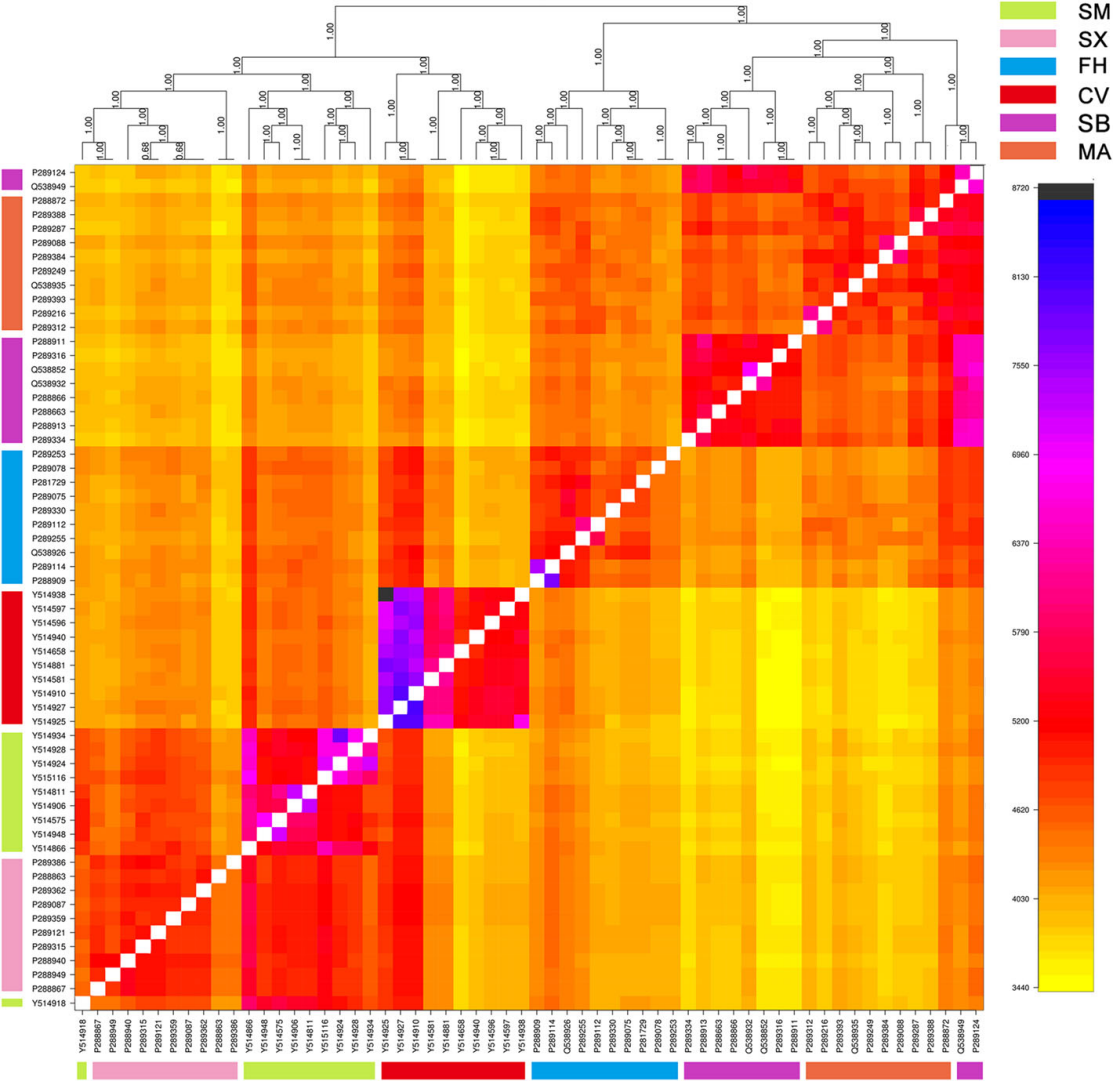
Output of the fineRADstructure individual (above diagonal) and average (below diagonal) coancestry coefficient matrix of the genomic data. The heatmap indicates pairwise coancestry between individuals, with blue and purple representing the highest levels, red and orange indicating intermediate levels, and yellow representing the lowest levels of shared coancestry

Next, we used fineRADstructure [22] to further evaluate population structure by assessing individual coancestry plots across samples (Fig. 3). First, fineRADstructure recovered two major genetic clusters, one including Fenghua ducks, Chinese spot-billed ducks and mallards. The second large group contained Shaoxing ducks, Shanma ducks and Cherry Valley Pekin ducks. Second, the resulting plot also showed higher shared coancestry within each species compared to that between species, and slightly higher coancestry levels were seen between mallards and Chinese spot-billed ducks, as did Shaoxing and Shanma ducks. These findings confirmed PCA, phylogenetic tree and structure results, supporting their close evolutionary relationship [23–25]. Finally, Fenghua ducks shown similar coancestry levels with mallards and Chinese spot-billed ducks, although local records indicated that Fenghua ducks were originated from mallards. Notably, some individuals showed a particularly high proportion of coancestry with others, which are unlikely to be explained by sibling statues and artificial selection, and may be due to complex introgression patterns among these duck population [26].

### Patterns of genomic variation and linkage disequilibrium

The genome-wide average genomic diversity (*θ*_*π*_) values were 5.949 × 10^− 4^ for mallard, 5.862 × 10^− 4^ for Chinese spot-billed duck, 5.815 × 10^− 4^ for Fenghua duck, 5.303 × 10^− 4^ for Shaoxing duck, 5.462 × 10^− 4^ for Shanma duck and 4.694 × 10^− 4^ for Cherry Valley Pekin duck (Supplementary Table S6), These values were much lower than in other animals (Supplementary Table S7). The wild duck had the greatest *θ*_*π*_ and *θ*_*W*_, suggesting that domestication reduces genetic diversity. Additionally, Linkage disequilibrium (LD) also showed that the wild ducks had a faster decay of the pairwise correlation coefficient (*r*^*2*^) than the domestic duck (Fig. 2d).

### Demographic history

**Fig. 4:**
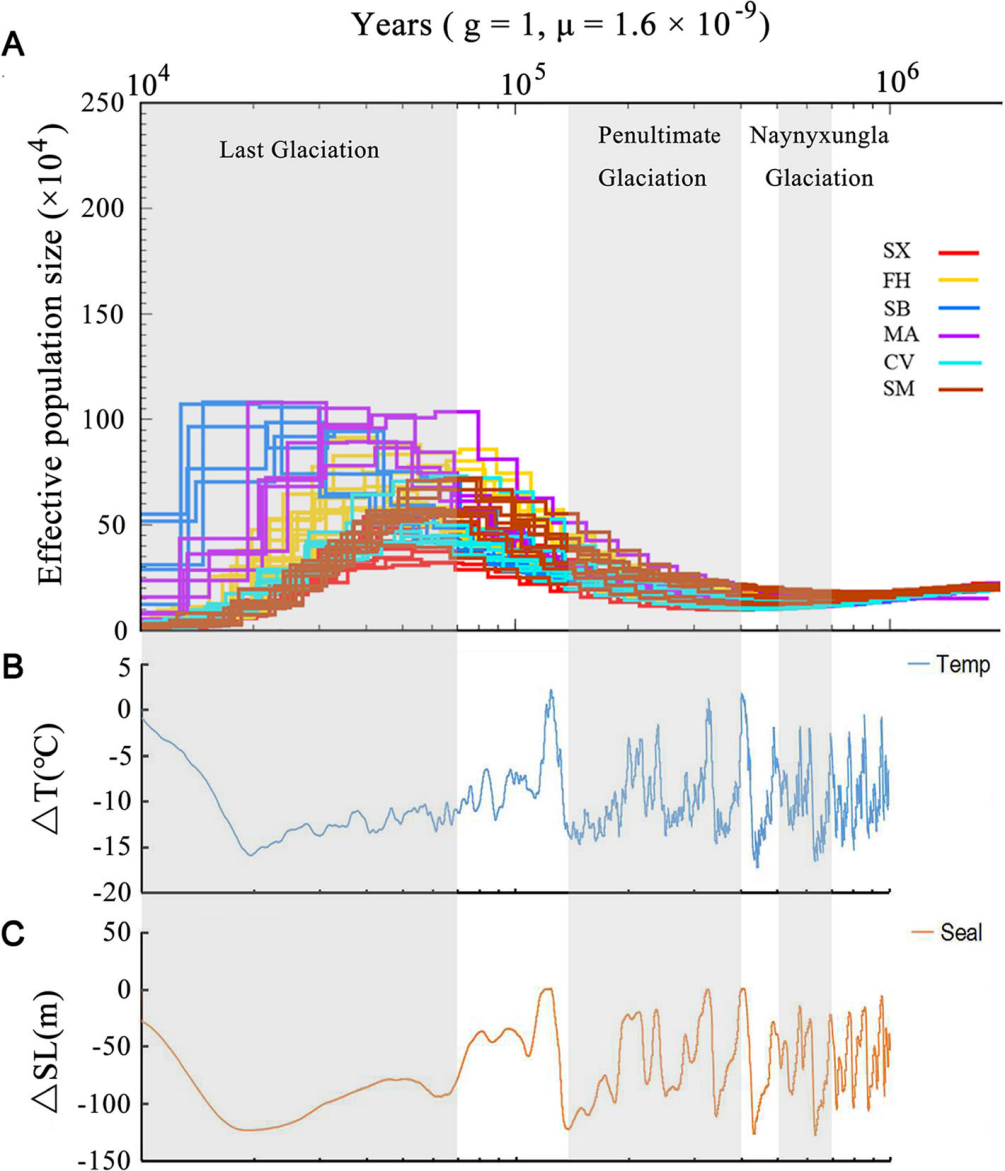
Demographic history of the duck populations. **a** Dynamic changes in the effective population sizes (*N*_e_) of six duck breeds inferred by PSMC. MA, mallards; SB, Chinese spot-billed ducks; FH, Fenghua ducks; SX, Shaoxing ducks; SM, Shanma ducks; CV, Cherry Valley Pekin ducks. The gray-shaded area (from left to right) refers to the Last Glaciation, the Penultimate Glaciation and the Naynyxungla Glaciation [28]. **b** The temperature from 10 KYA to 1000 KYA [29] (**c**) Sea level changed from 10 KYA to 1000 KYA [30]

We employed the pairwise sequentially Markovian coalescent (PSMC) method [27] to infer fluctuations in the ancestral effective population sizes (*N*_e_) of each breed in response to Quaternary climatic change (Fig. 4). From 1 million to 10 thousand years, all of the domestic breeds (Shaoxing, Shanma, Fenghua and Cherry Valley Pekin ducks) exhibited similar demographic trajectories with a peak in ancestral *N*_e_ at 50–60 thousand years ago followed by distinct declines (Supplementary Fig. S3). The decline occurred ~ 60 thousand year ago, coinciding with the beginning of the Last Glacial Maximum [28]. The effective population sizes of mallard and spot-billed duck appears to have increased rapidly after ~ 40 and ~ 20 thousand year ago, respectively (Supplementary Fig. S3).

### Genome-wide selective sweep test

**Fig. 5:**
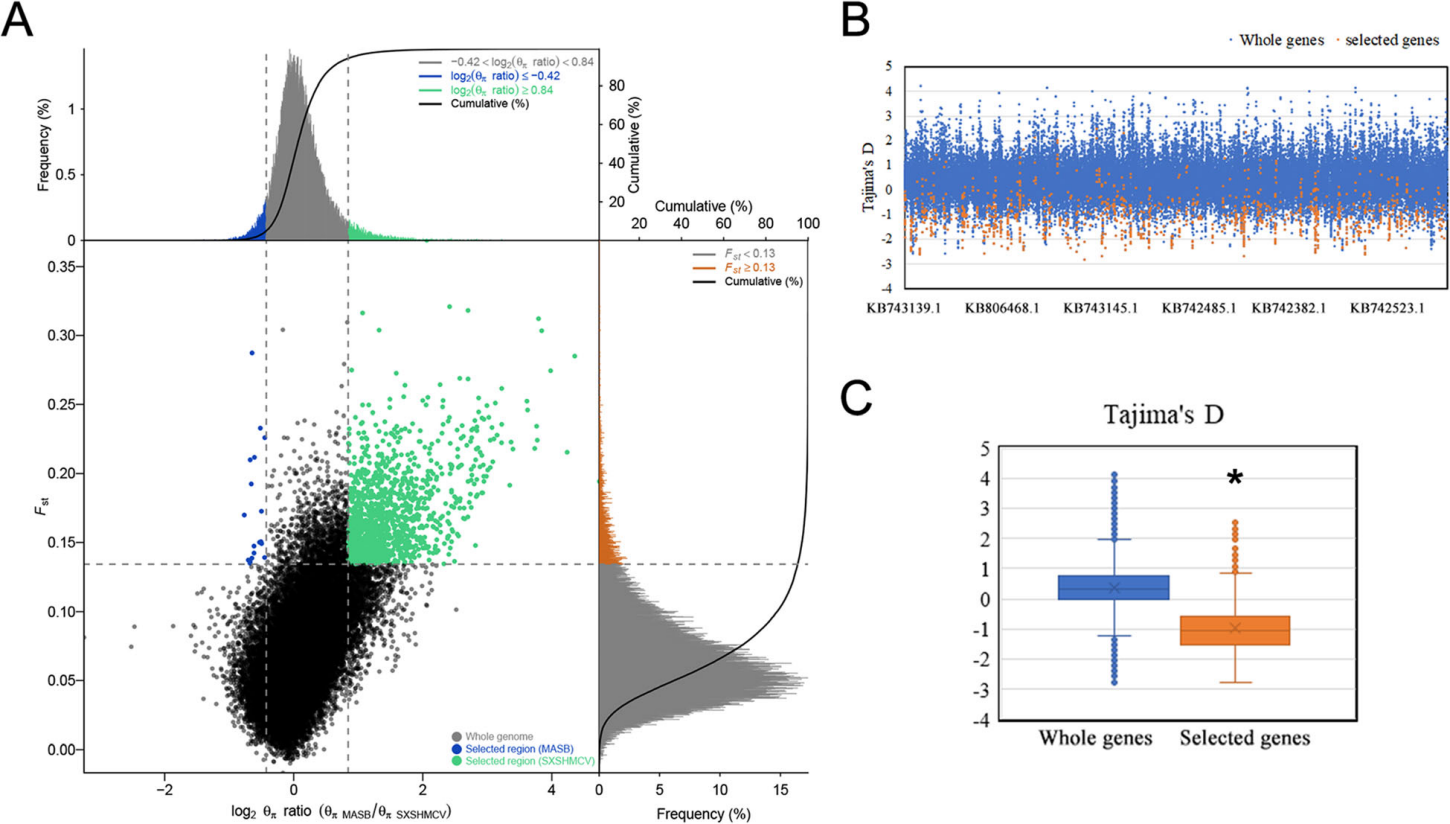
Identification of the genomic regions with strong selective sweep signals in domestic ducks. **a** Distribution of F_ST_ values and log_2_(θπ ratio) calculated in 40-kb sliding windows with 20-kb overlap between the domestic groups and the wild groups. The data points in blue are genomic regions under selection in wild groups, and the data points in green are genomic regions under selection in the domestic groups. **b** Distribution of Tajima’s D values for the whole genome and selected genes of domestic ducks. **c** Box plots of Tajima’s D values for the whole genome and selected genes of domestic ducks. *Indicates a significantly elevated Tajima’s D relative to the whole genes (Mann-Whitney U test *P* < 0.05)

To accurately detect the genomic footprints of selection, we pooled the domestic duck samples (Shaoxing, Shanma and Cherry Valley Pekin ducks) and compared them to the wild duck (Mallard and Chinese spot-billed duck), which are geographically close. Using the top 5% the *F*_ST_ values and *θ*_*π*_ ratio cutoffs (*F*_ST_ > 0.13 and log_2_ (*θ*_*π*_ ratio (*θ*_*π,* wild duck_/*θ*_*π*, domestic duck_) ≥0.84), we identified 665 candidate domestication regions (CDRs) containing 387 genes under selection in the domestic ducks (Fig. 5a, Supplementary Table S8). We also calculated the Tajima’s D value of selected genes, which were significantly lower than values for other genes (Fig. 5b, c). In addition, ten candidate genes (*Cmip*, *Tmem132b*, *Mphosph6*, *Smg7*, *Lyst*, *Zbtb37*, *Serpinc1*, *Npl*, *Tmem132c* and *Plcg2*) ranking within the top 10 *F*_ST_ values with log_2_ (*θ*_*π*_ ratio (*θ*_*π,* wild duck_/*θ*_*π*, domestic duck_) ≥ 0.84 were functionally involved in cellular adhesion function, type 2 diabetes, lipid metabolism, cell cycle, liver cell proliferation and muscle functioning [31–36] (Table 1).

**Table 1:** Positively selected genes with top 10 *F*_ST_ values in domestic ducks

Gene ID	Gene name	Gene symbol	F_ST_	Description
ENSAPLT00000011005	c-Maf inducing protein	Cmip	0.496	associating with language and reading, type 2 diabetes, obesity, lipid metabolism, breast and gastric cancer, negatively regulating T cell signaling
ENSAPLT00000011847	transmembrane protein 132B	Tmem132b	0.468	associating with excessive daytime sleepiness
ENSAPLT00000002396	M-phase phosphoprotein 6	Mphosph6	0.464	regulating cell cycle and ovary development, recruiting the exosome to the pre-rRNA, associating with coronary artery disease, IgA nephropathy and leukocyte telomere length
ENSAPLT00000016529	nonsense mediated mRNA decay factor	Smg7	0.463	regulating DNA damage response and nonsense-mediated mRNA decay
ENSAPLT00000006672	lysosomal trafficking regulator	Lyst	0.458	associating with Chediak-Higashi syndrome
ENSAPLT00000005034	zinc finger and BTB domain containing 37	Zbtb37	0.424	involving in aryl hydrocarbon receptor in hematopoietic stem cell functional regulation
ENSAPLT00000005105	serpin family C member 1	Serpinc1	0.424	associating with antithrombin deficiency and ovarian cancer
ENSAPLT00000004292	N-acetylneuraminate pyruvate lyase	Npl	0.421	regulating the cellular concentrations of sialic acid which is essential for muscle function
ENSAPLT00000012003	transmembrane protein 132C	Tmem132c	0.412	associating with pulmorary function, breast cancer, insulin secretion impairment, body weight
ENSAPLT00000011198	phospholipase C gamma 2	Plcg2	0.391	involving in inherited immune disorders, promoting liver cell proliferation

To identify the active pathways in the domestication of ducks, the positively selected genes in domestic ducks were mapped to the canonical reference pathways in the KEGG database. The top three enriched pathways were “pantothenate and CoA biosynthesis” (2 genes, *P* = 0.02667), “FoxO signaling pathway” (6 genes, *P* = 0.03002), and “inositol phosphate metabolism” (4 genes, *P* = 0.03511) (Supplementary Fig. S4, Supplementary Table S9). The positively selected genes of domestic ducks that were successfully annotated to 47 categories of Gene Ontology (GO), belonging to three parts: cellular components, molecular function and biological processes (Supplementary Fig. S5, Supplementary Table S10). Of these, the categories that were most represented in the “biological process” principal category were “cellular process” (137 genes), followed by “single-organism process” (123 genes). In the principal category of “cellular component”, the two categories most represented were “cell” (149 genes) and “cell part” (149 genes). Within the “molecular function” principal category belonged to the “bind” (107 genes).

### Positively selected genes involved in insulin signaling pathway

**Fig. 6:**
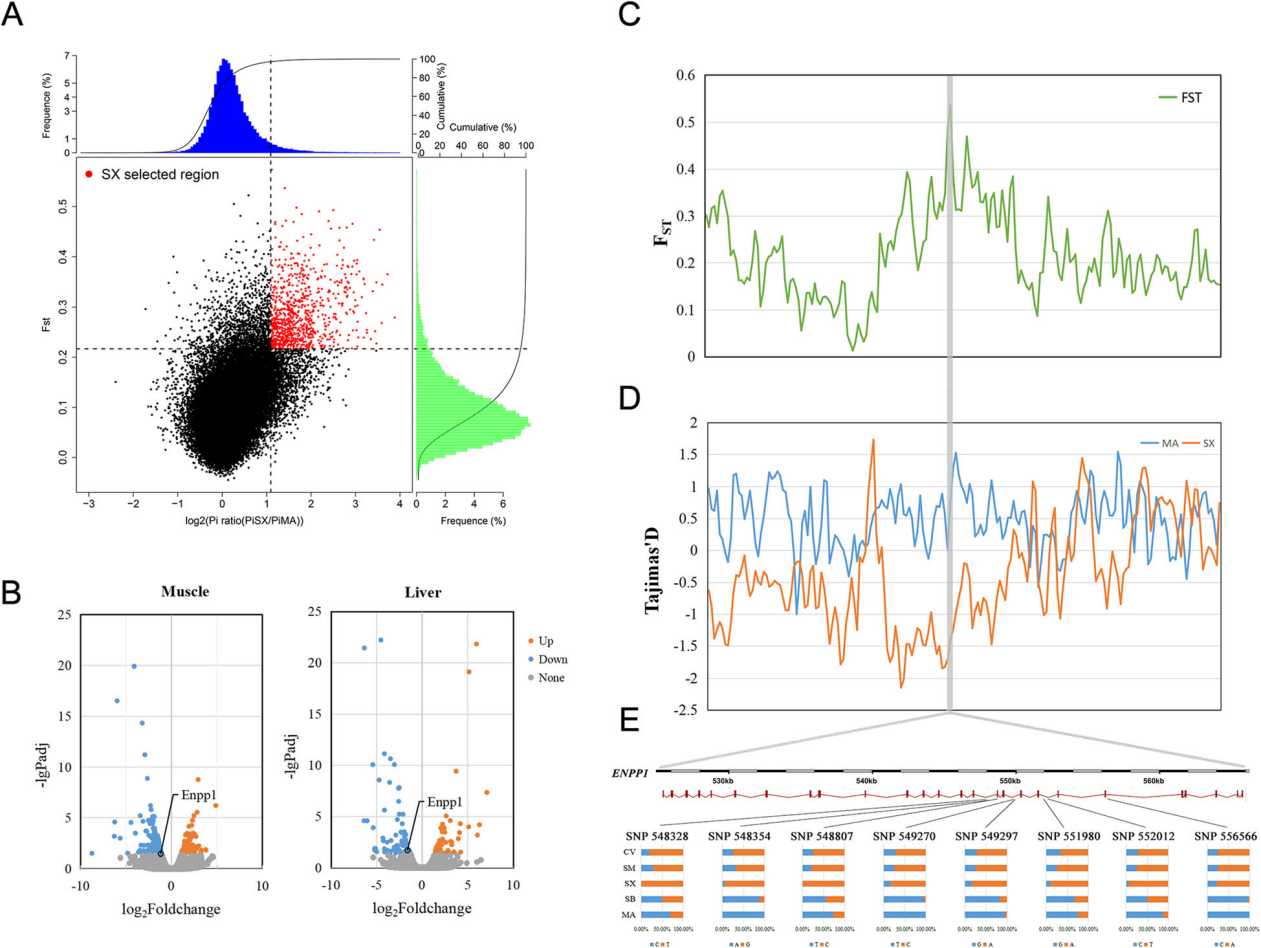
Genomic regions with strong selective signals in the Shaoxing duck (SX) and mallard (MA). **a** Distribution of *F*_ST_ values and log_2_(θπ ratio) calculated in 40-kb sliding windows with 20-kb overlap between Shaoxing duck and mallard. The data points in red are genomic regions under selection in the SX duck. **b** Volcano plot of the differentially expressed genes in the breast muscle and liver of the Shaoxing duck and mallard. Orange splashes indicate significantly upregulated genes. Blue splashes indicate significantly downregulated genes. Grey splashes indicate genes without significant different expression. **c** F_ST_ values around the *Enpp1* locus. **d** Tajima’s D values around the *Enpp1* locus. The blue and orange lines represent the mallard and Shaoxing duck, respectively. **e** Allele frequencies of eight SNPs within the *Enpp1* gene across 6 groups

Using the top 5% of the *F*_*ST*_ values and *θπ* ratio cutoffs based on sliding 40 kb windows for the Shaoxing ducks compared to wild mallards, we identified 497 candidate domestication regions (CDRs) containing 311 genes with both high *F*_*ST*_ values and a high *θπ* ratio (Fig. 6a). Six genes exhibiting strong selective sweep signals were significantly over-represented in insulin signaling pathway, including ectonucleotide pyrophosphatase /phosphpdisesterase-1 (*Enpp1*), ectonucleotide pyrophosphatase/phosphpdisesterase-3 (*Enpp3*), SHC adapter protein 4 (*Shc4*), SOS Ras/Rac guanine nucleotide exchange factor 1 (*Sos1*), neuroblastoma RAS viral oncogene homolog (*Nras*) and protein kinase cAMP-dependent type II regulatory subunit beta (*Prkar2b*).

Notably, we observed much higher *F*_ST_ values (Fig. 6c) and lower Tajima’s D values (Fig. 6d) for the target gene *Enpp1* compared to those in the adjacent genomic regions, providing further support that the candidate genes were reliable. 8 SNPs were found in this sliding window (Fig. 6e). We also used transcriptome sequencing to investigate the molecular signatures of domestication and identified significantly downregulation *Enpp1* expression in the muscle and liver tissues of Shaoxing ducks compared to mallards (Fig. 6b).

### Transcriptome differences in muscle, liver and cerebellum between Shaoxing ducks and mallards

Shaoxing duck is an outstanding representative of the local egg-laying duck breed in China, which contributes greatly to the Chinese waterfowl industry. To infer whether the potential positively selected genes between mallards and Shaoxing ducks could also affecting gene expression, we used Illumina paired-end RNA-seq approach to sequenced the breast muscle, liver and cerebellum of mallards and Shaoxing ducks. We obtained a total of 731 million clean reads, approximately 60.6% of them were successfully mapped to the duck genome (Supplementary Table S11). Compared with mallards, 319, 161 and 28 differentially expressed genes were identified in muscle, liver and cerebellum of Shaoxing ducks respectively (Supplementary Fig. S6, Supplementary Table S13–18). Six positively selected genes of resequencing, including *Coq9*, *Adamts9*, *Zcchc24*, *Eya1*, *Enpp3* and *Enpp1*, were differentially expressed in muscle (Supplementary Fig. S10). However, only *Enpp1* was found differntically expressed in liver. GO enrichment analysis was performed to discover the major functional categories represented in these genes. The GO categories related to cellular process, single-organism process, biological regulation, binding and catalytic (Supplementary Fig. S7, S8 and S9). There were a few KEGG pathways that were significantly enriched in muscle, including oxidative phosphorylation, fatty acid degradation, and cardiac muscle contraction (Supplementary Table S12).

## Discussion

### Population structure

In this study, we carried out whole-genome resequencing of 60 individuals to explore the genetic relationships among domestic ducks and wild ducks in eastern China. PCA and structure analysis clearly distinguished the wild ducks from domesticated ducks. Notably, individuals from Chinese spot-billed ducks and mallards were clustered together in PCA plot and separated in structure analysis only from K = 5, indicating a close relationship between them. Further, we constructed NJ tree based on whole-genome variants to infer phylogenetic relationships of these ducks. The result showed that four domestic ducks belong to the same large branch, which was consistent with previous studies suggesting a single domestication of domestic ducks [15, 37]. Additionally, phylogenetic tree confirmed that Chinese spot-billed ducks is a sister clade of mallard. Moreover, we found that Chinese spot-billed duck shared a relatively high degree of coancestry with mallard. Taken together, these results supported that Chinese spot-billed ducks and mallards were weakly genetically differentiated, although they were quite different in morphological appearance (Fig. 1). It was not surprising as hybridization is common between Chinese spot-billed ducks and mallards. Several mallard × Chinese spot-billed duck have recently been reported to occur on Hongkong, China [38], Khank Lake, Russia [39], and Tokyo, Japan [19]. The asymmetric hybridization and sex-biased gene flow between Chines spot-billed ducks and mallards was also confirmed [19, 25]. Due to the close genetic relationship between Chinese spot-billed duck and mallard, it was difficult to distinguish the role of Chinese spot-billed in domestic duck origination. Moreover, we found that the coancestry between Chinese spot-billed duck and domestic duck was similar to that between mallard and domestic duck. Taken together, our results indicated that Chinese spot-billed duck also shown substantial genetic contribution of Chinese domestic duck.

### Demographic history

We carried out PSMC analysis to infer fluctuations in historical effective population size (*N*e) of 6 breeds, and observed the similar trajectories for four Chinese domestic ducks with an apparent expansion during the Penultimate Glaciation and the Last Interglaciation, and a decline between 50 and 60 thousand years ago (Fig. 4a). However, mallard and Chines spot-billed duck population reached their pinnacle between 20 and 40 thousand years ago. The trend of *N*e is similar with previous studies of other Chinese domestic ducks, which increased in the interglacial periods and decreased in the Pleistocene [37, 40]. On coastal regions such as eastern China, Quaternary glacial-interglacial changes in climate (Fig. 4b) and sea level (Fig. 4c) had major effects on terrestrial plant and animal communities. The population expansion of interglacial periods can be explained by the warm and humid weather [41]. Beside, a severe reduction of *N*e approximately coinciding with the beginning of the Last Glacial Period or occurring during this period was observed in many avian populations, which may be due to climatic deterioration, habitat loss, and reduction of food supply [21]. Therefore, we believe that the similar reason is responsible for the bottleneck of ducks during Last Glacial Period.

### Selection for domestication

**Fig. 7:**
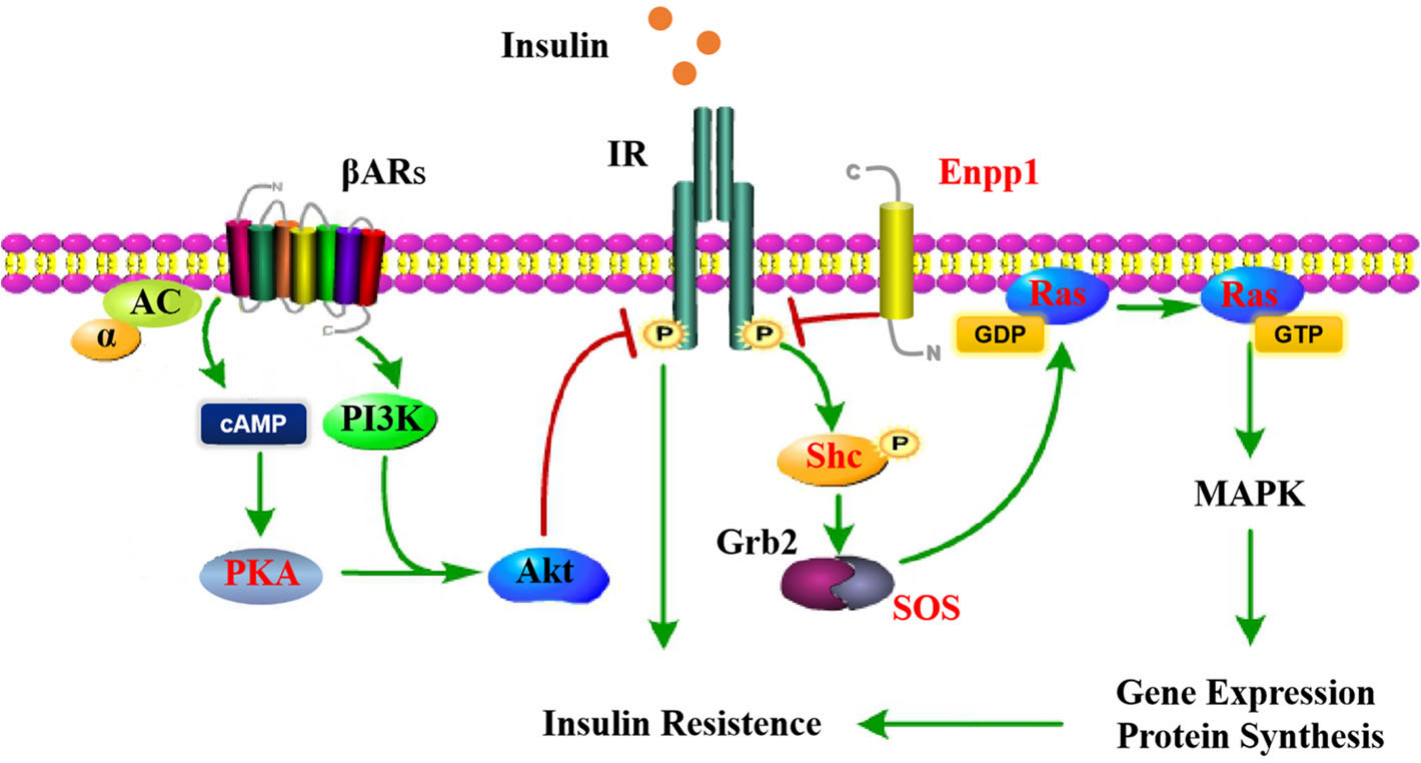
Selection of the insulin signaling pathway [46, 47]. The candidate genes under selection in duck domestication are shown in red

Shaoxing duck is a typical Chinese egg-type duck breed, which is under intense artificial selection to achieve excellent egg production. In order to the candidate regions for the targeted selection of Chinese native duck during domestication, we scanned the genome of Shaoxing ducks and mallards for regions with extreme Fst and the highest θπ ratio. On our results, the *Enpp1*, *Enpp3*, S*hc4, Sos1, Nras* and *Prkar2b,* which are related to the insulin signaling pathway, showed signals of positive selection in Shaoxing duck. *Enpp1* is the subtypes of ENPP family, which directly interacts with the insulin receptor and blocks the insulin signaling pathway [42], serving as a gatekeeper of insulin action. Transcriptome results showed that the expression level of Enpp1 in skeletal muscle and live of Shaoxing ducks was significantly lower than that of mallards, suggesting *Enpp1* may have played a crucial role in duck domestication by improving insulin sensitivity. *Enpp3* is positively associated with the serum ATP concentration, facilitating lipid deposition [43]. And *Enpp3* and *Prkar2b* were also identified as the targets of selection during the domestication of Pekin ducks and other indigenous Chinese ducks [7]. The protein encoded by Prkar2b is a regulatory subunits of the protein kinase A (PKA) and is involved in insulin resistance [44]. *Shc4* (also known as *ShcD)* serves as a phosphotyrosine adapter molecule that induces Ras GTPase and mitogen activated protein kinase (MAPK) activation [45]. Also, the *SOS1* and *NRAS* provide protein-making instructions that are involved in regulating the activation of the Ras/MAPK signaling pathway, which helps to control insulin signaling (Fig. 7).

Skeletal muscle plays an important role in regulating glucose uptake and body metabolism [48]. The association between increased muscle lipid content and insulin resistance has been confirmed [49]. And it has been observed that improving insulin sensitivity helped increase muscle mass in songbirds [50]. Additionally, our early study confirmed that Shaoxing ducks had lower intramuscular fat contents compared to mallards [51]. The positive selection of genes associated with insulin signaling and decrease of muscle lipid content indicated that insulin sensitivity of Shaoxing duck was improved with increasing muscle mass during domestication, to achieve people’s breeding object.

## Conclusion

In conclusion, we performed whole-genome resequencing to characterize the evolutionary origin of ducks in eastern China and the genome-wide signatures of artificial selection associated with domestication. We have shown that Chinese spot-billed duck was close related to mallard and contributed to domestic duck origination. Several candidate genes, important pathways and GO categories associated with artificial selection were functionally related to cellular adhesion, type 2 diabetes, lipid metabolism, the cell cycle, liver cell proliferation, and muscle functioning in domestic ducks. We found strong genomic evidence for the involvement of the insulin signaling pathway in the domestication of Shaoxing duck. These results advance our understanding of the genetic relationships between domestic and wild ducks, reveals the genetic footprints of domestication and shed light on the genetic mechanisms underlying species adaptation to captivity.

## Methods

### Sampling

The blood samples from all the 60 individual ducks (10 per breed) were collected from the wing vein using vacuum tubes containing EDTA-K_2_ as an anticoagulant. The spot-billed ducks, mallards and Fenghua ducks were captured in Fenghua City, Zhejiang Province, China (29°35′ N, 121°24′ E). The Shaoxing and Shanma ducks were collected in Zhuji City, Zhejiang Province, China (29°38′ N, 120°10′ E), and the Cherry Valley Pekin duck were raised in Huzhou City, Zhejiang Province, China (30°41′ N, 120°19′ E). From Shaoxing ducks and mallards, 3 randomly selected ducklings were killed by rapid decapitation and sterile dissection, and muscle and liver tissues were sampled and immediately snap frozen in liquid nitrogen.

### Sequencing and quality control

A total of 60 ducks, which were sampled from Eastern China, were sequenced on the Illumina HiSeq 2000 platform (Illumina, San Diego CA, USA). We generated a total of 401.491 Gb of raw sequence data (supplementary Table S2).

Raw reads in fastq format were firstly processed for quality using in-house C scripts. Specifically, low-quality reads were filtered out based as below [52]: reads with ≥10% unidentified nucleotides (N); reads with > 50% bases having phred quality < 5; reads with > 10 nt aligned to the adapter, allowing ≤10% mismatches; putative PCR duplicates generated by PCR amplification in the library construction process (read 1 and read 2 of two paired-end reads that were completely identical).

Consequently, 387.88 Gb were retained for assembly, of which the quality of 96.06 and 91.52% of the bases were ≥ Q20 and ≥ Q30, respectively.

### Reads mapping and SNP calling

The remaining high quality reads were mapped to the mallard (*Anas platyrhynchos*) reference genome (BGI_duck_1.0) [53] using Burrows-Wheeler Aligner (Version: 0.7.8) [54] with the command line was ‘aln -e 10 -t 4 -l 32 -i 15 -q 10’. SAMtools was used to remove the duplicated reads to reduce mismatch generated by PCR amplification before sequencing.

After alignment, we used SAMtools [55] to carry out SNP calling. The ‘mpileup’ command was used to identify SNPs with the parameters as ‘-q 1 -C 50 -S -D -m 2 -F 0.002’. The following filtering steps were applied in order to obtain high quality SNPs as follow: quality score > = 20; coverage depth > =2 and < =1000.

### Annotation of genetic variants

Using the ANNOVAR package [56], 2,809,077 high-quality SNPs were annotated according to the genome. Based on the genome annotation, SNPs were classified into several categories, such as exonic regions, intronic regions, splicing sites, upstream and downstream regions and intergenic regions. SNPs from coding exon regions were identified as either synonymous or nonsynonymous.

### Principal component analysis

The software GCTA [57] was used for PCA. The significance level of the eigenvectors was determined using the Tracey-Widom test to clarify the phylogenetic relationship among 60 individuals. The first three significant components were plotted (supplementary Fig. S2), and the discrete points to a degree reflect the real structure of population.

### Phylogenetic genetic analysis

First, we inferred an individual-based neighbor-joining (NJ) tree from 2,809,077 SNPs data matrix using TreeBeST (http://treesoft.sourceforge.net/treebest.shtml#inno) based on the *p*-distance. The bootstrap was set to 1000 times to evaluate the reliability of branch.

Second, the population genetic structure of 60 individuals was inferred by FRAPPE [58]. We set the number of cluster (K) from 2 to 6 and ran analysis with 10,000 iterations.

Third, population structure was assessed using fineRADstructure [22], which calculates recent shared co-ancestry based on patterns of genomic similarity. The vcf file was transformed using hapsFromVCF module, and then the co-ancestry matrix was calculated and used to identify populations. The MCMC chain ran with a thinning interval of 1000, a burnin of 100,000, and 100,000 iterations.

### Linkage disequilibrium analysis

We compared the pattern of linkage disequilibrium (LD) among 6 breeds using the 2.8 million high-quality SNPs. To estimate LD decay, we calculated the squared correlation coefficient (*r*^*2*^) between pairwise SNPs using the software Haploview [59]. The average *r*^*2*^ value was calculated for pairwise markers in a 500-kb window and averaged across the whole genome.

### Effective population size

We used a hidden Markov model (HMM) of pairwise sequentially Markovian coalescence (PSMC) to reconstructed demographic history of 60 individuals. Firstly, we called genotype each individual using the package SamTools [54] based on the command ‘mpileup’ with the parameter ‘-C 50 -D -S -m 2 -F 0.002’. Then, we performed the program ‘fq2psmcfa’ with the parameter ‘−N30, −t15, −r5 and − p ‘4 + 25*2 + 4 + 6″ to convert the consensus sequence to the required input format. A mutation rate (*μ*) of 1.6 × 10^− 9^ per bp per generation [14] and a generation time of 1 year were used for analysis. In addition, we applied a bootstrapping approach, repeating sampling 100 times to estimate the variance of simulated results.

### Selective sweep analysis

The nucleotide diversity (*θ*_*π*_), population-differentiation statistic (*F*_ST_), Tajima’s *D* statistic and Watterson estimator (*θ*_*W*_) were calculated with sliding windows of 40 kb that had 20 kb overlap between adjacent windows. The putative genomic regions under positive selection during domestication were extracted based on being the highest differences in genetic diversity (log_2_(*θ*_*π*_ ratio)) and the top 5% of *F*_ST_. We identified a total of 665 potential selective-sweep regions overlapping with 387 candidate genes in merging domestic ducks and 491 potential selective-sweep regions overlapping with 311 candidate genes in Shaoxing ducks, which would be used for subsequent analysis and discussion.

### Functional enrichment analysis

Gene Ontology term enrichment analysis was processed with those selective genes by goseq packages in R software. We used the GOSeq R package, in which gene length bias was corrected, to perform GO and functional pathway analysis on the candidate genes. The Gene ontology (GO) and the Kyoto Encyclopedia of Genes and Genomes (KEGG) pathways with a Benjamini adjusted *P*-values less than 0.05 were considered significantly enriched.

### RNA-seq and gene expression analysis

To infer whether the genes under selection could also affecting gene expression between Shaoxing ducks and mallards, we compared gene expression in breast muscle, liver and cerebellum between this two groups. 3 mature females from Shaoxing ducks and mallards respectively were selected for transcriptomics analysis.

All samples were individually sequenced by Illumina HiSeq 4000 sequencing platform. Perl scripts was used to ensure the quality of raw data. The reference genomes and the annotation file were downloaded from ENSEMBL database (http://www.ensembl.org/index.html). We used Bowtie/Bowtie 2 to build the genome index and TopHat v2.0.12 to map clean data to reference genome. And HTSeq v6.0 was used to count the number of fragments for each gene in each sample. The expression level of genes in each sample was estimated by FPKM (Fragments Per Kilobase Per Million Mapped Fragment). We used DEGseq v1.18.0 to analyze differential gene expression between Shaoxng and Shanma ducks. Genes with q ≤ 0.05 and |log_2_Ratio| ≥ 1 are identified as differentially expressed genes.

## Supplementary Information

**Additional file 1: Figure S1.** The number of unique and shared SNPs among six duck groups shown by a venn diagram**. Figure S2.** Principal component analysis (PCA). **Figure S3.** Demographic history of ducks. **Figure S4.** Distribution of KEGG pathway of domestic ducks is shown as a bar chart. **Figure S5.** Gene ontology (GO) classification for positively selected genes in domestication. **Figure S6.** The number of differential expression genes in muscle, liver and cerebellum. **Figure S7.** Function classifications of Gene Ontology terms of differential expression genes in muscle. **Figure S8.** Function classifications of Gene Ontology terms of differential expression genes in liver. **Figure S9.** Function classifications of Gene Ontology terms of differential expression genes in cerebellum. **Figure S10.** Venn diagram of selected genes (blue) and significantly expressed genes in muscle (yellow), liver (red) and cerebellum (green) of Shaoxing ducks. **Table S1.** Breeds included in the study and phenotypic description. **Table S2.** Statistics of genomic sequencing of six duck populations. **Table S3.** Summary of mapping and coverage of six duck populations. **Table S4.** Summary of SNPs of six duck populations included in the analyses. **Table S5.** Summary of the functional annotation statistics of SNP in ducks by ANNOVAR. **Table S6.** *θ*_*π*_ and *θ*_*W*_ for six duck populations. **Table S7.** Summary statistics for genomic nucleotide diversity in different species. **Table S8.** List of CDRs with top 5% highest *F*_ST_ values and log_2_ (*θ*_*π*_ ratio) in domestic ducks. **Table S9.** The KEGG pathway of the loci under selections in domestic ducks (Top 20). **Table S10.** The GO classification of the loci under selections in domestic ducks. **Table S11.** Summarize of sequence mapping of three tissues in Shaoxing ducks and mallards. **Table S12.** Pathway of KEGG differentially expressed gene in muscle of Shaoxing ducks. **Table S13.** The down-regulated genes in muscle of Shaoxing ducks (top 20). **Table S14.** The up-regulated genes in muscle of Shaoxing ducks (top 20). **Table S15.** The down-regulated genes in liver of Shaoxing ducks (top 20). **Table S16.** The up-regulated genes in liver of Shaoxing ducks (top 20). **Table S17.** The down-regulated genes in liver of Shaoxing ducks. **Table S18.** The up-regulated genes in liver of Shaoxing ducks.

## Data Availability

The raw sequence data files discussed in this experiment have been deposited in SRA and BioProject ID is PRJNA599025 (https://dataview.ncbi.nlm.nih.gov/object/PRJNA599 025).

## Abbreviations

YBP: Years before present
SB: Spot-billed duck
MA: Mallard
FH: Fenghua duck
SX: Shaoxing duck
SM: Shanma duck
CV: Cherry Valley Pekin duck
SNP: Single nucleotide polymorphic site
PCA: Principal component analysis
NJ tree: Neighbor-joining tree
PSMC: Pairwise sequentially Markovian coalescent
GO: Gene ontology
CDR: Candidate domestication region
Enpp1: Ectonucleotide pyrophosphatase /phosphpdisesterase-1
Enpp3: Ectonucleotide pyrophosphatase/phosphpdisesterase-3
Shc4: SHC adapter protein 4
Sos1: Ras/Rac guanine nucleotide exchange factor 1
Nras: Neuroblastoma RAS viral oncogene homolog
Prkar2b: Protein kinase cAMP-dependent type II regulatory subunit beta
