# Supplementary material for: Whole-genome resequencing provides insights into the population structure and domestication signatures of ducks in eastern China
Source: BMC Genomics. 2021 May 31;22:401. doi: 10.1186/s12864-021-07710-2 (PMC8165772; doi:10.1186/s12864-021-07710-2)
Supplement: Supplementary file 1 — Additional file 1: Figure S1. The number of unique and shared SNPs among six duck groups shown by a venn diagram. Figure S2. Principal component analysis (PCA). Figure S3. Demographic history of ducks. Figure S4. Distribution of KEGG pathway of domestic ducks is shown as a bar chart. Figure S5. Gene ontology (GO) classification for positively selected genes in domestication. Figure S6. The number of differential expression genes in muscle, liver and cerebellum. Figure S7. Function classifications of Gene Ontology terms of differential expression genes in muscle. Figure S8. Function classifications of Gene Ontology terms of differential expression genes in liver. Figure S9. Function classifications of Gene Ontology terms of differential expression genes in cerebellum. Figure S10. Venn diagram of selected genes (blue) and significantly expressed genes in muscle (yellow), liver (red) and cerebellum (green) of Shaoxing ducks. Table S1. Breeds included in the study and phenotypic description. Table S2. Statistics of genomic sequencing of six duck populations. Table S3. Summary of mapping and coverage of six duck populations. Table S4. Summary of SNPs of six duck populations included in the analyses. Table S5. Summary of the functional annotation statistics of SNP in ducks by ANNOVAR. Table S6. θπ and θW for six duck populations. Table S7. Summary statistics for genomic nucleotide diversity in different species. Table S8. List of CDRs with top 5% highest FST values and log2 (θπ ratio) in domestic ducks. Table S9. The KEGG pathway of the loci under selections in domestic ducks (Top 20). Table S10. The GO classification of the loci under selections in domestic ducks. Table S11. Summarize of sequence mapping of three tissues in Shaoxing ducks and mallards. Table S12. Pathway of KEGG differentially expressed gene in muscle of Shaoxing ducks. Table S13. The down-regulated genes in muscle of Shaoxing ducks (top 20). Table S14. The up-regulated genes in muscle of Shaoxing ducks [file 12864_2021_7710_MOESM1_ESM.docx]

Supplementary Materials


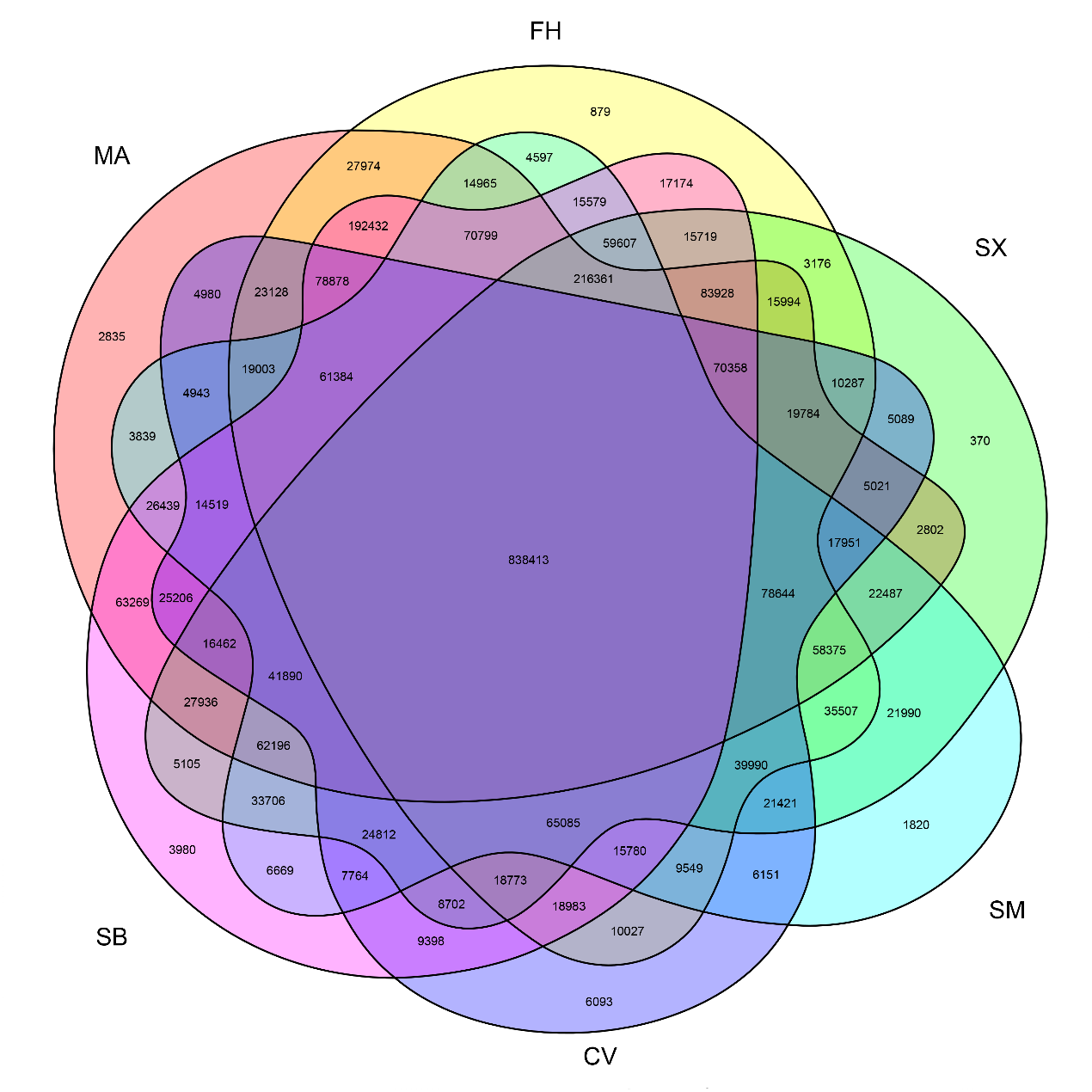


Supplementary Fig. S1 The number of unique and shared SNPs among six duck groups shown by a venn diagram


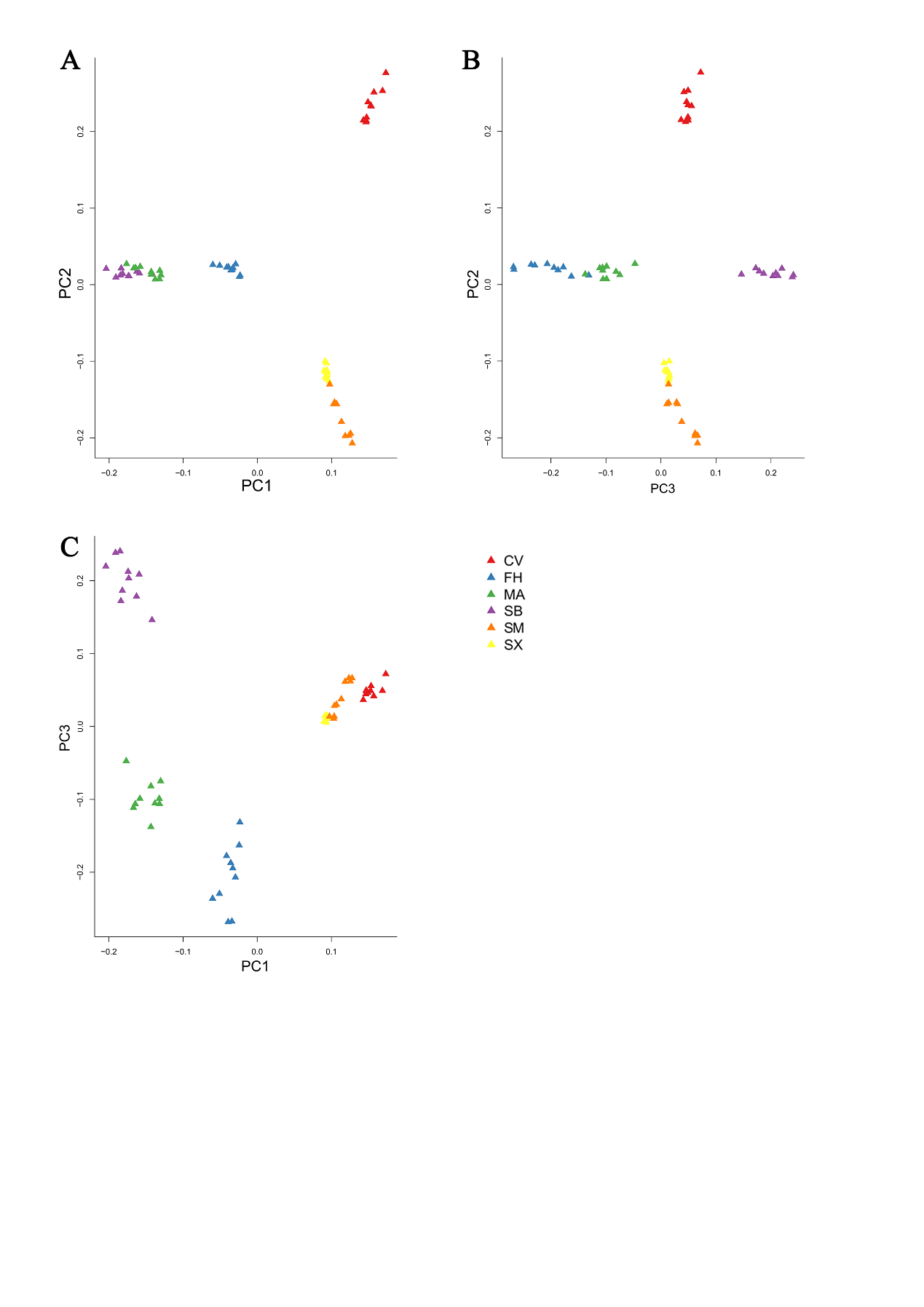


Supplementary Fig. S2 Principal component analysis (PCA) of (A) PC1 and PC2; (B) PC2 and PC3; (C) PC1 and PC3. The points of Shaoxing ducks are in close proximity to that of Shanma ducks in three figures.


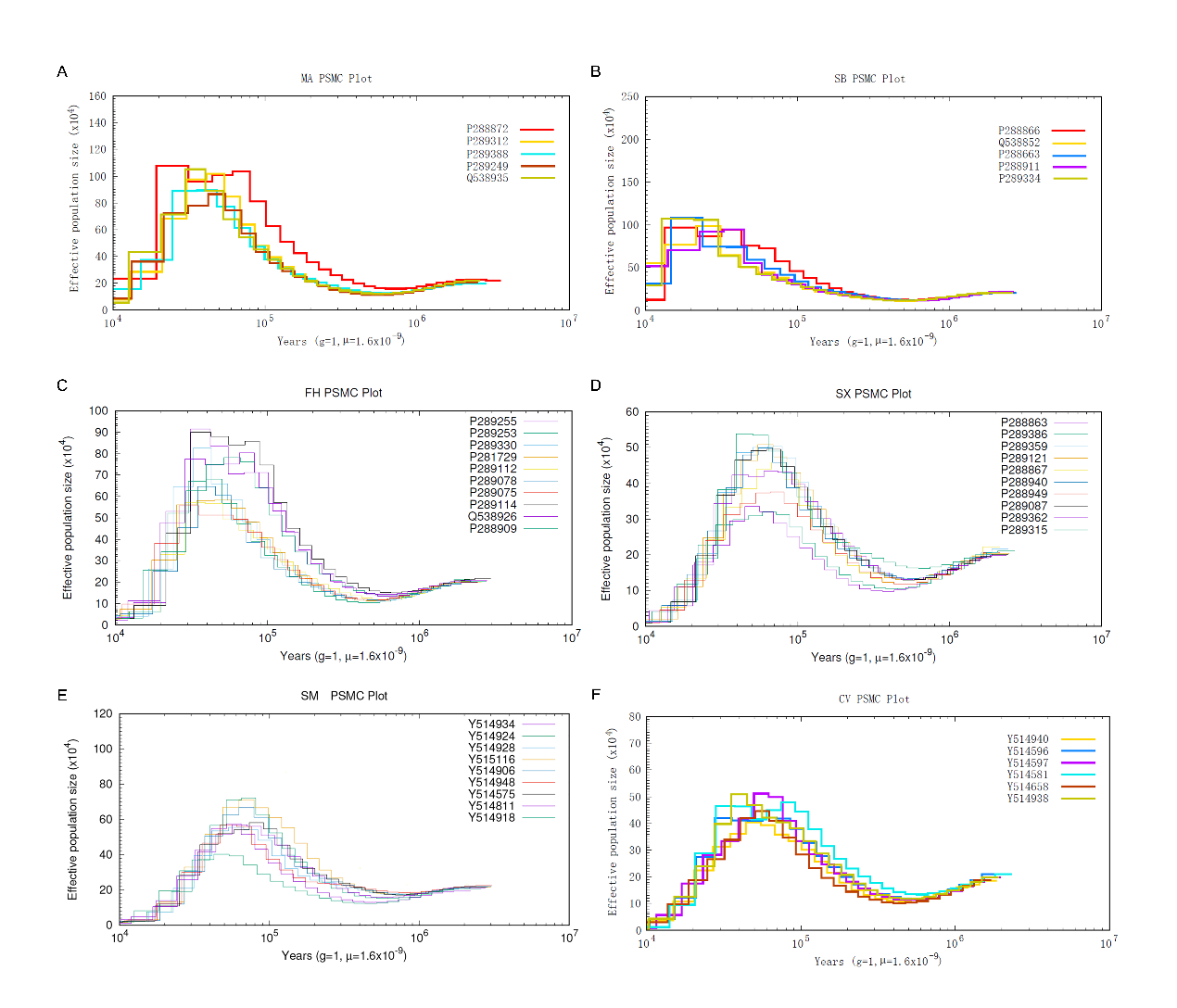


Supplementary Fig. S3 Demographic history of ducks. (A) PSMC analysis results of MA population. (B) PSMC analysis results of SB population. (C) PSMC analysis results of FH population. (D) PSMC analysis results of SX population. (E) PSMC analysis results of SM population. (F) PSMC analysis results of CV population.


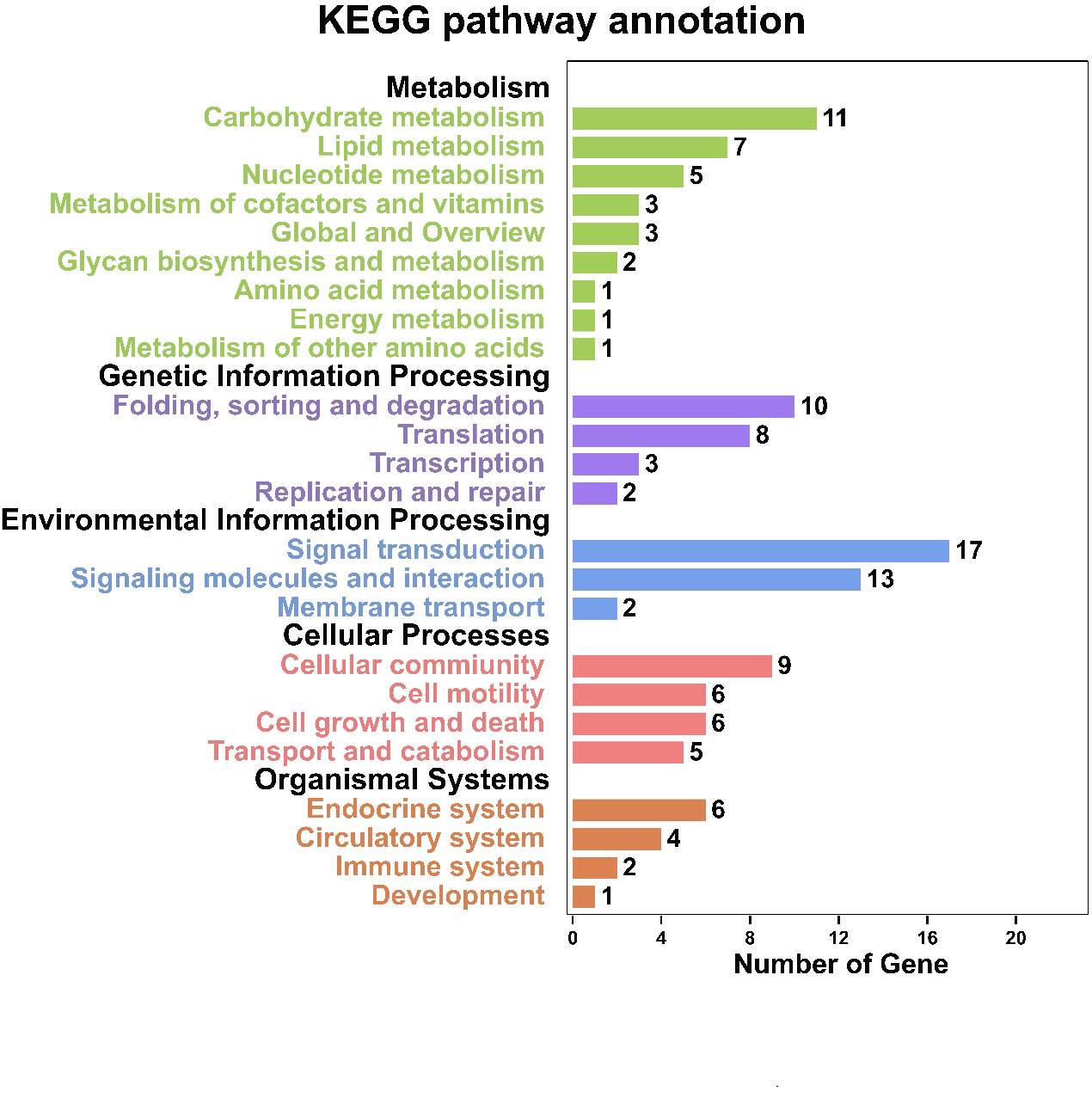


Supplementary Fig. S4 Distribution of KEGG pathway of domestic ducks is shown as a bar chart. The number of gene is shown along X-axis while the different KEGG pathways are shown along Y-axis.


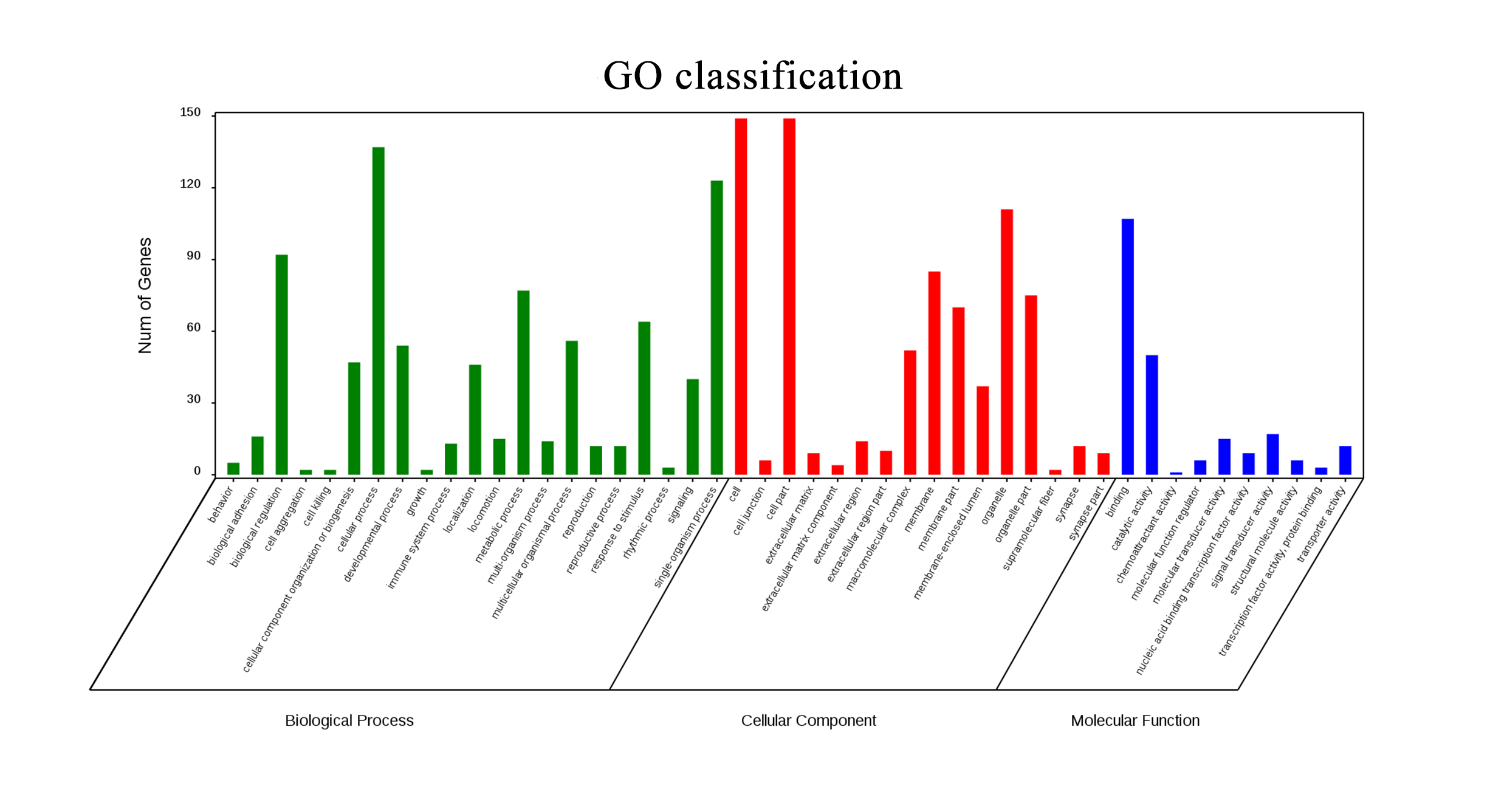


Supplementary Fig. S5 Gene ontology (GO) classification for positively selected genes in domestication. The number of gene is shown along Y-axis while the different GO categories are shown along X-axis.

Supplementary Fig. S6 The number of differential expression genes in muscle, liver and cerebellum


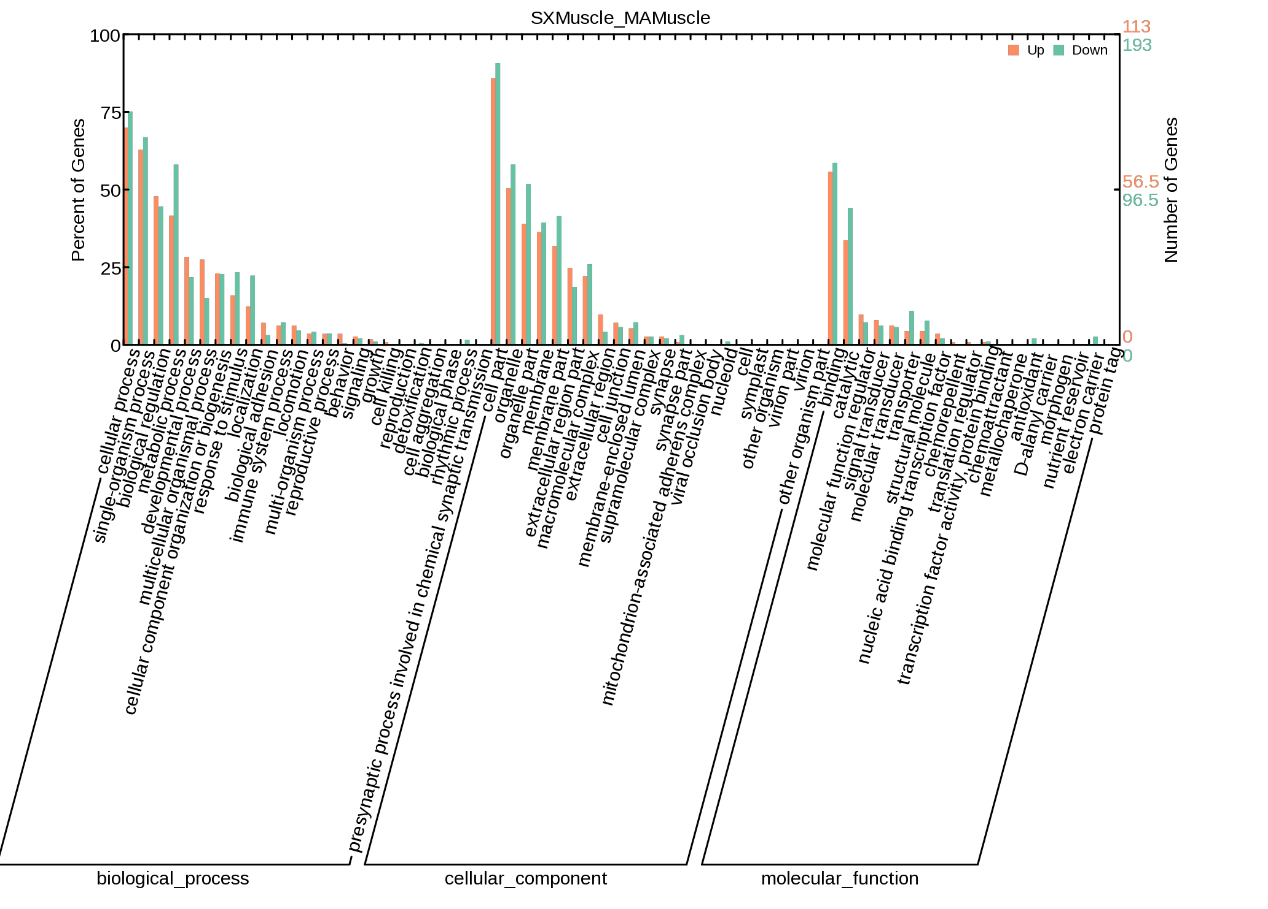


Supplementary Fig. S7 Function classifications of Gene Ontology terms of differential expression genes in muscle. The left ordinate represent the proportion of the GO term, and the right ordinate is the gene number of this term. Red indicates the up-regulated genes, and green indicates the down-regulated genes.


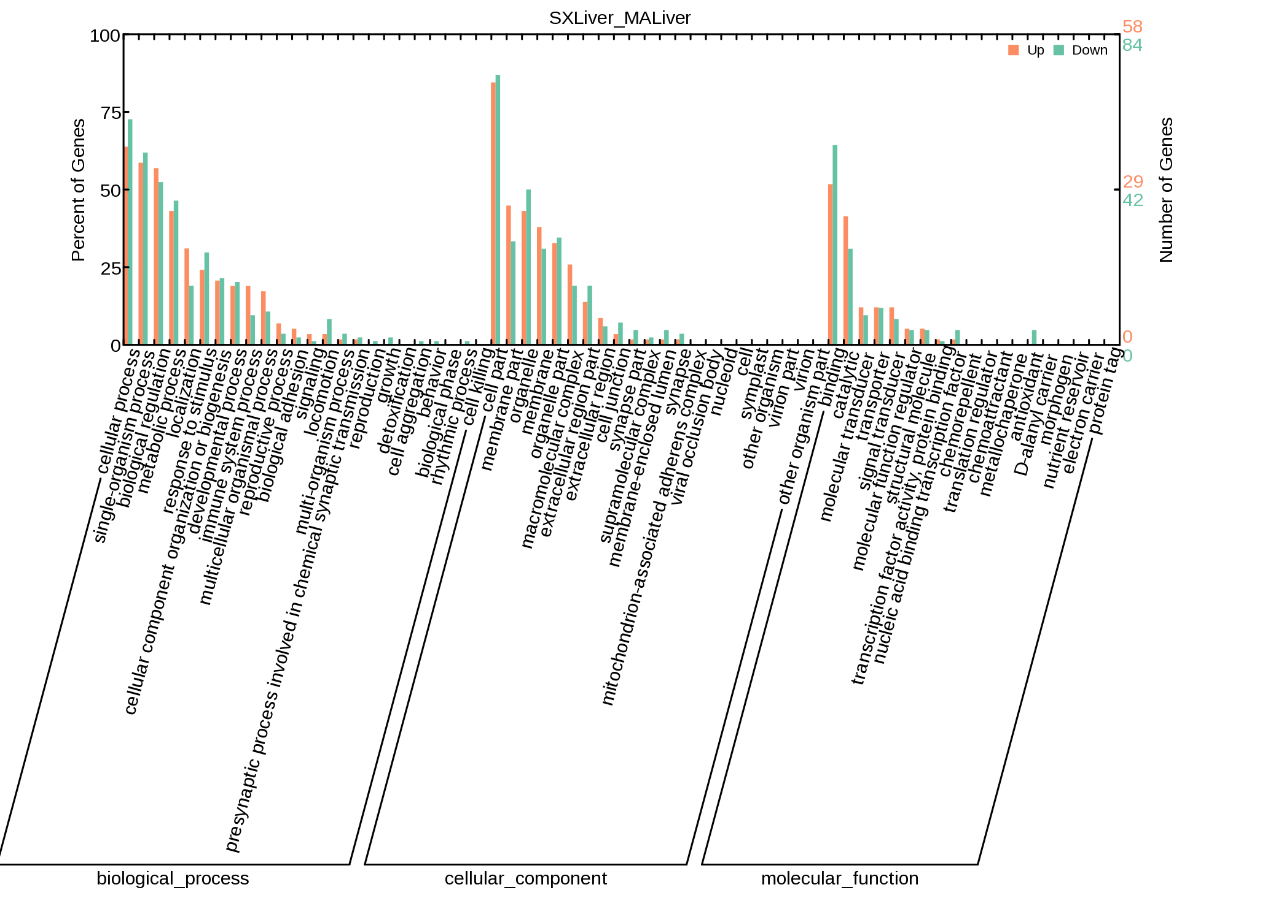


Supplementary Fig. S8 Function classifications of Gene Ontology terms of differential expression genes in liver. The left ordinate represent the proportion of the GO term, and the right ordinate is the gene number of this term. Red indicates the up-regulated genes, and green indicates the down-regulated genes.


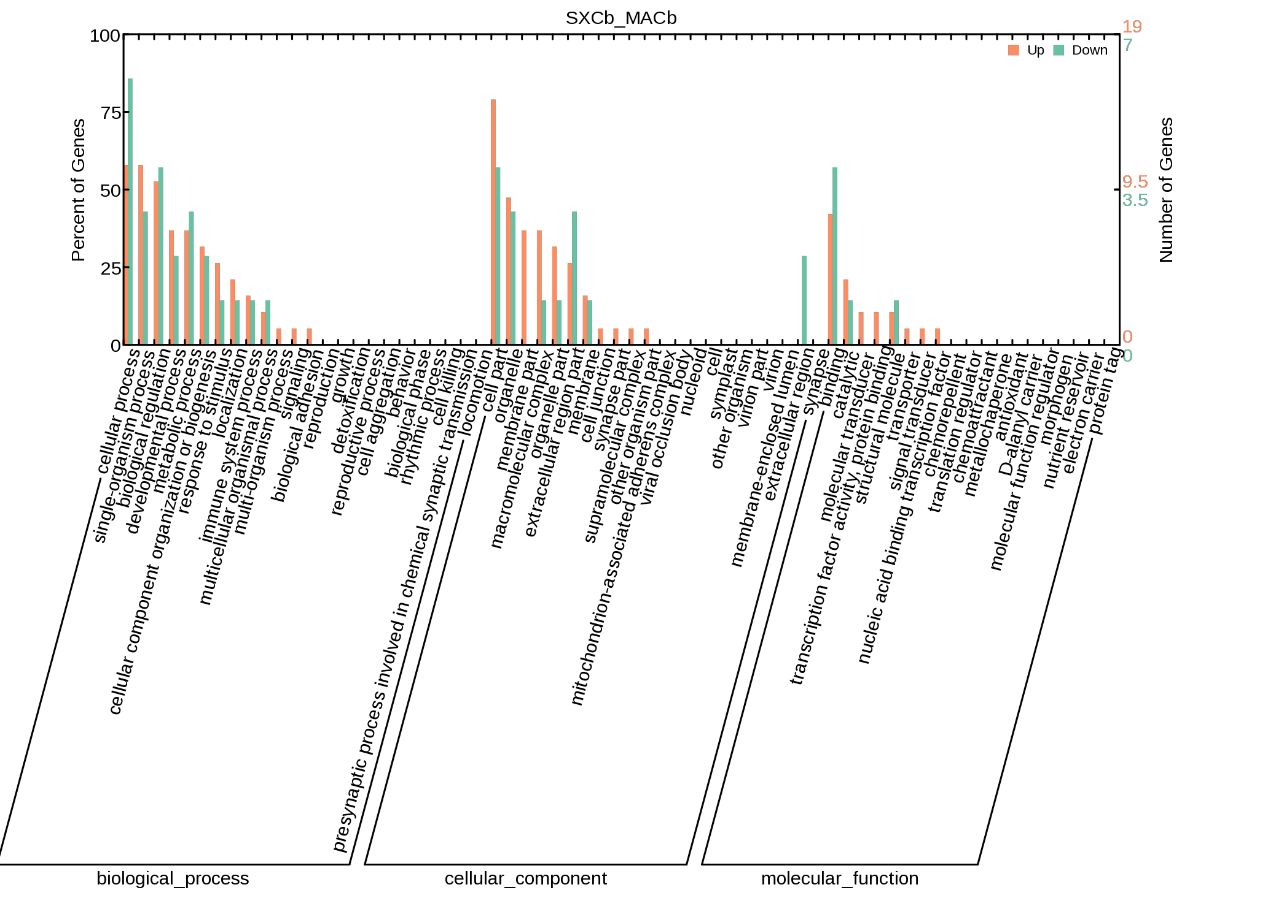


Supplementary Fig. S9 Function classifications of Gene Ontology terms of differential expression genes in cerebellum. The left ordinate represent the proportion of the GO term, and the right ordinate is the gene number of this term. Red indicates the up-regulated genes, and green indicates the down-regulated genes.


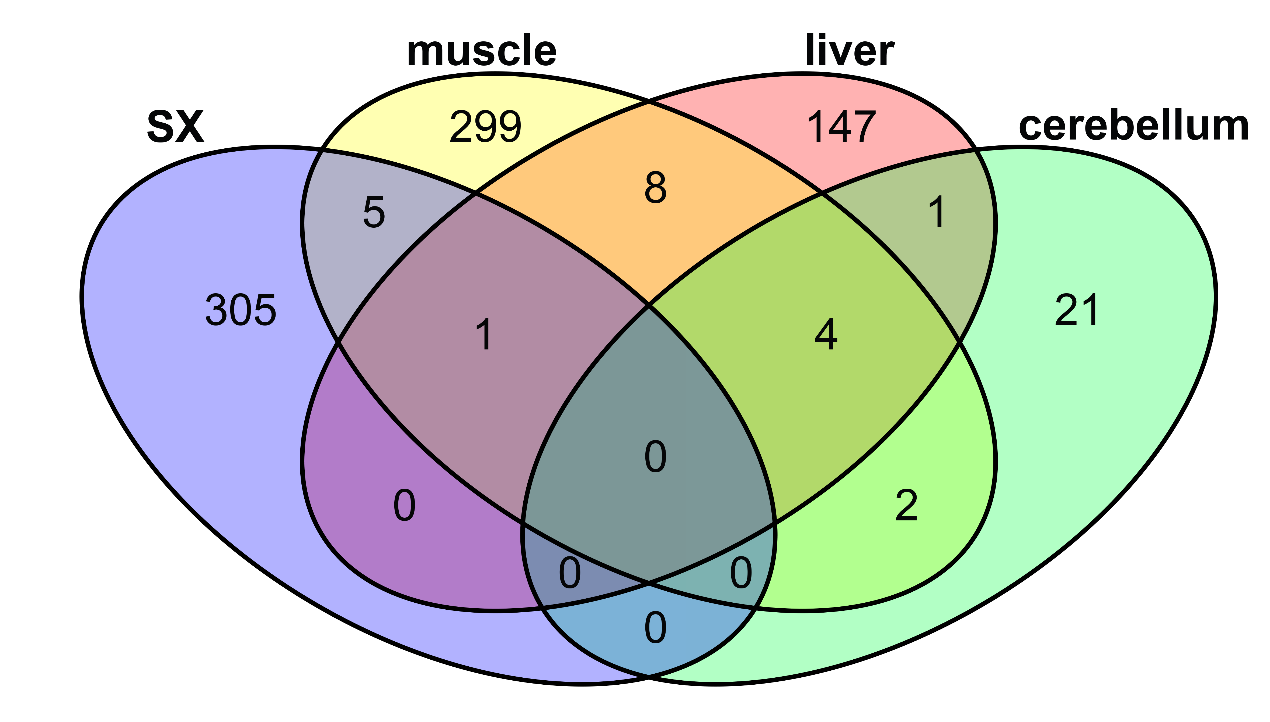


Supplementary Fig. S10 Venn diagram of selected genes (blue) and significantly expressed genes in muscle (yellow), liver (red) and cerebellum (green) of Shaoxing ducks.

Supplementary Tables

Supplementary Table S1. Breeds included in the study and phenotypic description

| Breed | Type | Phenotypic description |
| --- | --- | --- |
| Mallard | Wild duck | The male ducks are matallic green on their head and neck, gray on their wings and belly, dark brown on their body and have yellow beak. The female ducks have mainly brown-speckled plumage and orange red beak with many dark spots. Both sexes have white-bordered and blue speculum on their wings. |
| Chinese Spot-billed Duck | Wild duck | These are mainly light brown ducks with paler head and neck and tan brown belly. The beaks are black tipped bright yellow. The wings are whitish with black flight feathers below and have a blue speculum. The legs and feet are bright orange. |
| Shaoxing Duck | Egg type | This duck is slender and have yellow bill and orange feet. The male duck has a white collar that seperate the green head and brown body, and white belly. The female duck has light brown body with a paler collar. |
| Shama Duck | Egg type | The male duck has dark green head and neck, tan body, white belly and orange feet. The beak of male is blue-yellow with black tip. The female duck has light gray back, light brown belly, yellow beak and orange feet. |
| Fenghua Duck | Egg and meat type | These ducks have simillar appearance with mallards. |
| Cherry Valley Pekin Duck | Meat type | This duck is large and solidly built. The plumage is creamy white with a yellowish tinge. The beak is short and orange-coloured. The legs and feet are a yellowish orange. |

Supplementary Table S2. Statistics of genomic sequencing of six duck populations.

| Sample | breed | Raw Base  (bp) | Clean Base  (bp) | Effective Rate  (%) | Error Rate  (%) | Q20  (%) | Q30  (%) | GC Content  (%) |
| --- | --- | --- | --- | --- | --- | --- | --- | --- |
| Y514581 | CV | 6,533,194,200 | 6,462,274,200 | 98.91 | 0.03 | 95.93 | 91.22 | 42.73 |
| Y514596 | CV | 5,791,020,900 | 5,723,121,000 | 98.83 | 0.03 | 95.83 | 91.10 | 42.86 |
| Y514597 | CV | 6,007,902,900 | 5,936,809,200 | 98.82 | 0.03 | 96.03 | 91.46 | 42.61 |
| Y514658 | CV | 5,524,394,400 | 5,453,904,000 | 98.72 | 0.03 | 95.98 | 91.41 | 42.78 |
| Y514881 | CV | 6,436,633,500 | 6,371,151,600 | 98.98 | 0.03 | 95.64 | 90.66 | 42.45 |
| Y514910 | CV | 10,318,641,900 | 10,178,369,400 | 98.64 | 0.03 | 95.59 | 90.12 | 43.86 |
| Y514925 | CV | 8,957,637,300 | 8,850,498,300 | 98.80 | 0.03 | 95.98 | 91.38 | 42.61 |
| Y514927 | CV | 9,632,760,300 | 9,516,111,000 | 98.79 | 0.03 | 95.69 | 90.27 | 43.43 |
| Y514938 | CV | 6,089,936,700 | 6,024,513,300 | 98.93 | 0.03 | 95.90 | 91.19 | 42.36 |
| Y514940 | CV | 5,570,843,700 | 5,499,125,700 | 98.71 | 0.03 | 95.72 | 90.91 | 42.67 |
| P281729 | FH | 6,229,839,900 | 6,189,873,900 | 99.36 | 0.03 | 95.92 | 91.36 | 42.46 |
| P288909 | FH | 6,833,508,300 | 6,779,187,600 | 99.21 | 0.03 | 96.03 | 91.62 | 43.16 |
| P289075 | FH | 6,213,127,800 | 6,166,217,700 | 99.24 | 0.03 | 95.86 | 91.30 | 42.74 |
| P289078 | FH | 6,269,563,800 | 6,215,130,000 | 99.13 | 0.03 | 95.83 | 91.25 | 43.39 |
| P289112 | FH | 6,515,290,200 | 6,458,615,400 | 99.13 | 0.03 | 95.94 | 91.44 | 43.33 |
| P289114 | FH | 7,209,048,000 | 7,154,875,800 | 99.25 | 0.03 | 95.94 | 91.45 | 42.59 |
| P289253 | FH | 5,807,459,700 | 5,764,559,100 | 99.26 | 0.03 | 96.06 | 91.67 | 42.70 |
| P289255 | FH | 6,872,199,900 | 6,819,311,100 | 99.23 | 0.03 | 95.92 | 91.42 | 42.72 |
| P289330 | FH | 6,145,863,000 | 6,090,861,000 | 99.11 | 0.03 | 95.96 | 91.50 | 43.06 |
| Q538926 | FH | 6,797,516,700 | 6,747,015,300 | 99.26 | 0.03 | 95.81 | 91.19 | 42.82 |
| P288872 | MA | 7,777,491,900 | 7,727,799,900 | 99.36 | 0.03 | 96.01 | 91.54 | 42.66 |
| P289088 | MA | 5,864,935,500 | 5,831,550,300 | 99.43 | 0.03 | 96.30 | 92.06 | 42.75 |
| P289216 | MA | 6,486,920,100 | 6,428,559,000 | 99.10 | 0.03 | 96.14 | 91.83 | 43.22 |
| P289249 | MA | 6,233,058,000 | 6,192,772,500 | 99.35 | 0.03 | 96.24 | 91.96 | 42.83 |
| P289287 | MA | 6,175,804,200 | 6,132,768,900 | 99.30 | 0.03 | 95.81 | 91.17 | 42.79 |
| P289312 | MA | 6,404,539,800 | 6,353,858,700 | 99.21 | 0.03 | 95.98 | 91.52 | 43.17 |
| P289384 | MA | 6,574,561,500 | 6,512,864,400 | 99.06 | 0.03 | 95.93 | 91.44 | 44.16 |
| P289388 | MA | 6,509,927,700 | 6,467,841,600 | 99.35 | 0.03 | 96.23 | 91.95 | 42.90 |
| P289393 | MA | 6,892,477,500 | 6,828,551,100 | 99.07 | 0.03 | 95.67 | 90.75 | 43.74 |
| Q538935 | MA | 6,433,198,800 | 6,392,567,400 | 99.37 | 0.03 | 96.00 | 91.51 | 42.86 |
| P288663 | SB | 6,380,013,000 | 6,327,822,000 | 99.18 | 0.03 | 97.01 | 93.34 | 43.19 |
| P288866 | SB | 6,157,292,700 | 6,122,565,300 | 99.44 | 0.03 | 96.09 | 91.67 | 42.75 |
| P288911 | SB | 6,118,113,600 | 6,077,032,500 | 99.33 | 0.03 | 96.88 | 93.07 | 43.28 |
| P288913 | SB | 6,269,608,800 | 6,225,407,400 | 99.29 | 0.03 | 96.43 | 92.21 | 43.09 |
| P289124 | SB | 9,575,010,000 | 9,500,328,600 | 99.22 | 0.03 | 96.44 | 92.26 | 43.19 |
| P289316 | SB | 5,931,841,800 | 5,892,548,700 | 99.34 | 0.02 | 97.02 | 93.34 | 43.14 |
| P289334 | SB | 6,094,672,500 | 6,052,644,000 | 99.31 | 0.03 | 96.44 | 92.28 | 43.04 |
| Q538852 | SB | 6,093,615,000 | 6,046,461,000 | 99.23 | 0.03 | 96.78 | 92.91 | 43.27 |
| Q538932 | SB | 6,585,876,300 | 6,531,150,000 | 99.17 | 0.03 | 96.31 | 92.03 | 43.23 |
| Q538949 | SB | 7,867,398,000 | 7,829,285,700 | 99.52 | 0.03 | 96.74 | 92.74 | 42.45 |
| Y514575 | SM | 7,377,276,300 | 7,306,740,900 | 99.04 | 0.03 | 96.55 | 92.37 | 43.03 |
| Y514811 | SM | 7,029,386,400 | 6,974,218,200 | 99.22 | 0.03 | 96.38 | 92.02 | 42.57 |
| Y514866 | SM | 8,936,115,900 | 8,858,368,500 | 99.13 | 0.03 | 96.23 | 91.78 | 42.75 |
| Y514906 | SM | 7,531,848,600 | 7,461,924,600 | 99.07 | 0.03 | 96.25 | 91.81 | 43.25 |
| Y514918 | SM | 6,134,908,800 | 6,080,565,300 | 99.11 | 0.03 | 95.41 | 90.28 | 43.08 |
| Y514924 | SM | 6,606,762,900 | 6,559,059,000 | 99.28 | 0.03 | 95.47 | 89.97 | 42.15 |
| Y514928 | SM | 6,955,778,100 | 6,883,298,100 | 98.96 | 0.03 | 95.40 | 89.94 | 43.56 |
| Y514934 | SM | 6,112,745,100 | 6,056,362,800 | 99.08 | 0.03 | 95.41 | 89.91 | 42.73 |
| Y514948 | SM | 7,277,882,100 | 7,192,391,100 | 98.83 | 0.03 | 96.18 | 91.71 | 43.92 |
| Y515116 | SM | 7,185,339,000 | 7,129,912,500 | 99.23 | 0.03 | 95.60 | 90.21 | 42.30 |
| P288863 | SX | 5,479,598,400 | 5,414,701,200 | 98.82 | 0.03 | 95.97 | 91.36 | 42.69 |
| P288867 | SX | 6,106,622,700 | 6,041,441,100 | 98.93 | 0.03 | 96.17 | 91.71 | 42.56 |
| P288940 | SX | 6,234,129,600 | 6,156,561,000 | 98.76 | 0.03 | 96.05 | 91.53 | 43.27 |
| P288949 | SX | 6,053,116,500 | 5,980,602,300 | 98.80 | 0.03 | 96.22 | 91.87 | 43.24 |
| P289087 | SX | 6,330,501,900 | 6,248,131,500 | 98.70 | 0.03 | 96.27 | 91.94 | 43.11 |
| P289121 | SX | 6,510,861,600 | 6,440,452,800 | 98.92 | 0.03 | 96.17 | 91.72 | 42.25 |
| P289315 | SX | 6,676,837,200 | 6,583,051,500 | 98.60 | 0.03 | 95.99 | 91.46 | 43.80 |
| P289359 | SX | 6,564,972,600 | 6,482,793,600 | 98.75 | 0.03 | 96.28 | 91.94 | 42.77 |
| P289362 | SX | 6,440,978,700 | 6,358,015,200 | 98.71 | 0.03 | 96.27 | 91.97 | 42.97 |
| P289386 | SX | 5,762,704,800 | 5,703,385,200 | 98.97 | 0.03 | 95.37 | 90.31 | 43.05 |
| Average | | 6,691,518,450 | 6,629,798,000 | 99.08 | 0.03 | 96.06 | 91.52 | 42.97 |

Supplementary Table S3. Summary of mapping and coverage of six duck populations.

| sample | breed | clean reads | mapped reads | mapping rate  (%) | depth | Coverage  (%) | Coverage at least 4X (%) |
| --- | --- | --- | --- | --- | --- | --- | --- |
| Y514581 | CV | 43081828 | 40962754 | 95.08 | 6.43 | 96.79 | 63.76 |
| Y514596 | CV | 38154140 | 36254190 | 95.02 | 6.22 | 95.69 | 55.46 |
| Y514597 | CV | 39578728 | 37637462 | 95.10 | 6.24 | 96.03 | 57.90 |
| Y514658 | CV | 36359360 | 34545569 | 95.01 | 6.06 | 95.29 | 52.64 |
| Y514881 | CV | 42474344 | 40463063 | 95.26 | 6.47 | 96.78 | 63.43 |
| Y514910 | CV | 67855796 | 64361793 | 94.85 | 8.56 | 98.53 | 85.19 |
| Y514925 | CV | 59003322 | 56151557 | 95.17 | 7.64 | 98.19 | 82.91 |
| Y514927 | CV | 63440740 | 60356960 | 95.14 | 8.19 | 98.39 | 83.37 |
| Y514938 | CV | 40163422 | 38276757 | 95.30 | 6.27 | 96.17 | 59.27 |
| Y514940 | CV | 36660838 | 34848086 | 95.06 | 6.13 | 95.26 | 52.74 |
| P281729 | FH | 41265826 | 39355774 | 95.37 | 6.25 | 96.23 | 59.11 |
| P288909 | FH | 45194584 | 43005800 | 95.16 | 6.55 | 96.54 | 62.82 |
| P289075 | FH | 41108118 | 39245802 | 95.47 | 6.20 | 96.36 | 59.24 |
| P289078 | FH | 41434200 | 39262509 | 94.76 | 6.24 | 95.91 | 56.98 |
| P289112 | FH | 43057436 | 40929314 | 95.06 | 6.34 | 96.27 | 60.25 |
| P289114 | FH | 47699172 | 45590416 | 95.58 | 6.66 | 97.10 | 66.81 |
| P289253 | FH | 38430394 | 36658940 | 95.39 | 6.17 | 95.11 | 52.78 |
| P289255 | FH | 45462074 | 43358674 | 95.37 | 6.57 | 96.67 | 63.74 |
| P289330 | FH | 40605740 | 38589589 | 95.03 | 6.29 | 95.55 | 55.56 |
| Q538926 | FH | 44980102 | 42864353 | 95.30 | 6.50 | 96.84 | 63.59 |
| P288872 | MA | 51518666 | 49184330 | 95.47 | 6.88 | 97.63 | 73.18 |
| P289088 | MA | 38877002 | 37265051 | 95.85 | 6.26 | 95.22 | 55.41 |
| P289216 | MA | 42857060 | 40640207 | 94.83 | 6.45 | 95.67 | 57.95 |
| P289249 | MA | 41285150 | 39521653 | 95.73 | 6.29 | 95.99 | 59.05 |
| P289287 | MA | 40885126 | 38899437 | 95.14 | 6.29 | 95.93 | 57.07 |
| P289312 | MA | 42359058 | 40150270 | 94.79 | 6.43 | 95.73 | 57.43 |
| P289384 | MA | 43419096 | 40929045 | 94.27 | 6.56 | 95.41 | 57.71 |
| P289388 | MA | 43118944 | 41246885 | 95.66 | 6.43 | 96.36 | 62.13 |
| P289393 | MA | 45523674 | 43171965 | 94.83 | 6.63 | 96.24 | 62.53 |
| Q538935 | MA | 42617116 | 40701219 | 95.50 | 6.36 | 96.23 | 60.01 |
| P288663 | SB | 42185480 | 40111946 | 95.08 | 6.37 | 95.96 | 58.87 |
| P288866 | SB | 40817102 | 39052727 | 95.68 | 6.32 | 95.90 | 58.24 |
| P288911 | SB | 40513550 | 38576673 | 95.22 | 6.30 | 95.50 | 56.00 |
| P288913 | SB | 41502716 | 39587059 | 95.38 | 6.25 | 96.29 | 58.51 |
| P289124 | SB | 63335524 | 60228719 | 95.09 | 7.62 | 98.31 | 84.07 |
| P289316 | SB | 39283658 | 37492509 | 95.44 | 6.26 | 94.83 | 53.70 |
| P289334 | SB | 40350960 | 38401678 | 95.17 | 6.11 | 96.18 | 56.37 |
| Q538852 | SB | 40309740 | 38374712 | 95.20 | 6.20 | 95.51 | 55.04 |
| Q538932 | SB | 43541000 | 41178961 | 94.58 | 6.34 | 96.49 | 59.34 |
| Q538949 | SB | 52195238 | 50149691 | 96.08 | 7.01 | 97.76 | 74.41 |
| Y514575 | SM | 48711606 | 46161027 | 94.76 | 6.73 | 97.45 | 70.60 |
| Y514811 | SM | 46494788 | 44272620 | 95.22 | 6.54 | 97.38 | 68.97 |
| Y514866 | SM | 59055790 | 56115318 | 95.02 | 7.51 | 98.35 | 80.76 |
| Y514906 | SM | 49746164 | 47022074 | 94.52 | 6.76 | 97.44 | 70.50 |
| Y514918 | SM | 40537102 | 38164913 | 94.15 | 6.18 | 96.33 | 57.86 |
| Y514924 | SM | 43727060 | 41803413 | 95.60 | 6.55 | 97.03 | 65.51 |
| Y514928 | SM | 45888654 | 43450898 | 94.69 | 6.64 | 96.89 | 64.78 |
| Y514934 | SM | 40375752 | 38374074 | 95.04 | 6.31 | 96.15 | 58.24 |
| Y514948 | SM | 47949274 | 45201766 | 94.27 | 6.65 | 97.20 | 67.75 |
| Y515116 | SM | 47532750 | 45422987 | 95.56 | 6.80 | 97.54 | 70.61 |
| P288863 | SX | 36098008 | 34311738 | 95.05 | 6.05 | 95.13 | 51.52 |
| P288867 | SX | 40276274 | 38408052 | 95.36 | 6.26 | 96.20 | 59.23 |
| P288940 | SX | 41043740 | 38851929 | 94.66 | 6.33 | 96.09 | 58.27 |
| P288949 | SX | 39870682 | 37655528 | 94.44 | 6.34 | 95.36 | 54.98 |
| P289087 | SX | 41654210 | 39522876 | 94.88 | 6.42 | 95.93 | 59.01 |
| P289121 | SX | 42936352 | 40994193 | 95.48 | 6.39 | 96.75 | 63.53 |
| P289315 | SX | 43887010 | 41279069 | 94.06 | 6.53 | 96.38 | 61.20 |
| P289359 | SX | 43218624 | 41074824 | 95.04 | 6.42 | 96.65 | 63.07 |
| P289362 | SX | 42386768 | 40221573 | 94.89 | 6.43 | 96.16 | 59.66 |
| P289386 | SX | 38022568 | 35892035 | 94.40 | 6.14 | 95.43 | 53.54 |
| average | | 44198653 | 42029750 | 95.09 | 6.52 | 96.41 | 62.24 |

Supplementary Table S4 Summary of SNPs of six duck populations included in the analyses.

| Population | Number of individuals | Number of SNPs |
| --- | --- | --- |
| SB | 10 | 2,217,306 |
| MA | 10 | 2,213,195 |
| FH | 10 | 2,211,132 |
| CV | 10 | 1,598,468 |
| SX | 10 | 1,947,941 |
| SM | 10 | 1,922,235 |

Supplementary Table S5. Summary of the functional annotation statistics of SNP in ducks by ANNOVAR.

| Category | | Number of SNPs |
| --- | --- | --- |
| Upstream | | 33799 |
| Exonic | Stop gain | 74 |
|  | Stop loss | 4 |
|  | Synonymous | 42,463 |
|  | Non-synonymous | 12,084 |
| Intronic | | 887,051 |
| Splicing | | 177 |
| Downstream | | 37,213 |
| upstream/downstream | | 634 |
| Intergenic | | 1,795,578 |
| ts | | 2,044,203 |
| tv | | 764,874 |
| ts/tv | | 2.672 |
| Total | | 2,809,077 |

| Population | *θ_π_*（×10^-4^） | *θ_W_*（×10^-4^） | Tajima’D |
| --- | --- | --- | --- |
| MA | 5.949 | 5.901 | 0.0235459 |
| SB | 5.862 | 5.895 | -0.0157630 |
| FH | 5.815 | 5.945 | -0.0626571 |
| SX | 5.303 | 5.212 | 0.0500372 |
| SM | 5.462 | 5.122 | 0.1899537 |
| CV | 4.694 | 4.275 | 0.2810593 |

Supplementary Table S6. *θ_π_* and *θ_W_* for six duck populations

Supplementary Table S7. Summary statistics for genomic nucleotide diversity in different species.

| Species | Diversity Index (*θ_π_*) |
| --- | --- |
| duck | 0.4694-0.5949×10^-3^ |
| sheep^a^ | 1.889-2.513×10^-3^ |
| Yak^b^ | 1.31-1.40×10^-3^ |
| Pigeon^c^ | 3.6×10^-3^ |
| Dog^d^ | 0.75-1.38×10^-3^ |

^a^ Yang, J., W. Li, F. Lv, S. He, S. Tian, W. Peng, Y. Sun, Y. Zhao, X. Tu, M. Zhang, X. Xie, Y. Wang, J. Li, Y. Liu, Z. Shen, F. Wang, G. Liu, H. Lu, J. Kantanen, J. Han, M. Li, and M. Liu. 2016. Whole-Genome Sequencing of Native Sheep Provides Insights into Rapid Adaptations to Extreme Environments. MOLECULAR BIOLOGY AND EVOLUTION 33:2576-2592.

^b^ Qiang, Q., L. Wang, K. Wang, Y. Yang, M. Tao, Z. Wang, Z. Xiao, Z. Ni, F. Hou, and R. Long. 2015. Yak whole-genome resequencing reveals domestication signatures and prehistoric population expansions. Nature Communications 6:10283.

^c^ Shapiro, M. D., Z. Kronenberg, C. Li, E. T. Domyan, H. Pan, M. Campbell, H. Tan, C. D. Huff, H. Hu, A. I. Vickrey, S. C. A. Nielsen, S. A. Stringham, H. Hu, E. Willerslev, M. T. P. Gilbert, M. Yandell, G. Zhang, and J. Wang. 2013. Genomic Diversity and Evolution of the Head Crest in the Rock Pigeon. SCIENCE 339:1063-1067.

^d^ Gou, X., Z. Wang, N. Li, F. Qiu, Z. Xu, D. Yan, S. Yang, J. Jia, X. Kong, Z. Wei, S. Lu, L. Lian, C. Wu, X. Wang, G. Li, T. Ma, Q. Jiang, X. Zhao, J. Yang, B. Liu, D. Wei, H. Li, J. Yang, Y. Yan, G. Zhao, X. Dong, M. Li, W. Deng, J. Leng, C. Wei, C. Wang, H. Mao, H. Zhang, G. Ding, and Y. Li. 2014. Whole-genome sequencing of six dog breeds from continuous altitudes reveals adaptation to high-altitude hypoxia. GENOME RESEARCH 24:1308-1315.

Supplementary Table S8. List of CDRs with top 5% highest FST values and log_2_ (*θ_π_* ratio) in domestic ducks

| Gene-ID | CDRs list | Starting position | Ending position | *F_ST_* | log_2_(*θ_π_* ratio) |
| --- | --- | --- | --- | --- | --- |
| KB742984.1 | CMIP | 3400001 | 3440000 | 0.496 | 3.844 |
| KB742984.1 | CMIP | 3360001 | 3400000 | 0.475 | 2.574 |
| KB742984.1 | CMIP | 3340001 | 3380000 | 0.474 | 1.412 |
| KB742969.1 | TMEM132B | 80001 | 120000 | 0.468 | 2.063 |
| KB743146.1 | MPHOSPH6 | 80001 | 120000 | 0.464 | 3.795 |
| KB742446.1 | SMG7 | 520001 | 560000 | 0.463 | 1.323 |
| KB743543.1 | LYST | 380001 | 420000 | 0.458 | 2.417 |
| KB742984.1 | CMIP | 3380001 | 3420000 | 0.440 | 3.224 |
| KB743146.1 | MPHOSPH6 | 60001 | 100000 | 0.427 | 3.981 |
| KB742446.1 | ZBTB37/SERPINC1/HEBP2 | 720001 | 760000 | 0.424 | 1.570 |
| KB742446.1 | NPL | 660001 | 700000 | 0.421 | 1.669 |
| KB742969.1 | TMEM132C | 800001 | 840000 | 0.412 | 1.794 |
| KB742446.1 | NPL | 680001 | 720000 | 0.403 | 2.528 |
| KB742984.1 | PLCG2 | 3420001 | 3460000 | 0.391 | 3.613 |
| KB743139.1 | SLC7A10/LRP3 | 740001 | 780000 | 0.384 | 1.492 |
| KB742432.1 | STX7 | 180001 | 220000 | 0.379 | 3.774 |
| KB742432.1 | STX7 | 200001 | 240000 | 0.378 | 2.702 |
| KB742432.1 | MOXD1 | 220001 | 260000 | 0.377 | 1.895 |
| KB743316.1 | ROBO2 | 1000001 | 1040000 | 0.375 | 1.437 |
| KB742446.1 | NPL/ZBTB37/HEBP2 | 700001 | 740000 | 0.373 | 2.538 |
| KB742446.1 | LAMC2/NMNAT2 | 560001 | 600000 | 0.372 | 1.475 |
| KB743546.1 | PREP | 300001 | 340000 | 0.372 | 2.276 |
| KB743073.1 | ENSAPLT00000003613 | 1280001 | 1320000 | 0.372 | 1.722 |
| KB743070.1 | ENSAPLT00000011274 | 80001 | 120000 | 0.371 | 1.046 |
| KB743070.1 | ENSAPLT00000011274 | 60001 | 100000 | 0.370 | 1.070 |
| KB742446.1 | NMNAT2/SMG7 | 540001 | 580000 | 0.369 | 1.513 |
| KB744033.1 | LIMK2/HNF1A/RNF185/ENSAPLT00000002965 | 820001 | 860000 | 0.369 | 2.702 |
| KB743546.1 | PREP | 320001 | 360000 | 0.366 | 2.627 |
| KB742685.1 | ENSAPLT00000016920 | 1500001 | 1540000 | 0.366 | 1.702 |
| KB742446.1 | RC3H1/SERPINC1 | 740001 | 780000 | 0.365 | 1.684 |
| KB742543.1 | ADAMTS5 | 320001 | 360000 | 0.364 | 1.230 |
| KB742969.1 | TMEM132C | 780001 | 820000 | 0.364 | 1.607 |
| KB742446.1 | ENSAPLT00000006207 | 840001 | 880000 | 0.364 | 0.983 |
| KB744292.1 | ZNF438 | 300001 | 340000 | 0.361 | 2.079 |
| KB742446.1 | LAMC1/LAMC2 | 580001 | 620000 | 0.358 | 1.606 |
| KB742446.1 | LAMC1 | 620001 | 660000 | 0.357 | 1.424 |
| KB742668.1 | MTX2 | 540001 | 580000 | 0.357 | 1.960 |
| KB743950.1 | CDH19 | 120001 | 160000 | 0.356 | 0.862 |
| KB742992.1 | PROSER1/NHLRC3/STOML3 | 20001 | 60000 | 0.356 | 1.960 |
| KB743913.1 | MARCH6/ENSAPLT00000013710 | 100001 | 140000 | 0.353 | 2.141 |
| KB742446.1 | LAMC1 | 640001 | 680000 | 0.352 | 1.551 |
| KB744033.1 | LIMK2/RNF185 | 840001 | 880000 | 0.351 | 2.312 |
| KB742617.1 | FGF23/FGF6 | 1440001 | 1480000 | 0.350 | 1.461 |
| KB742543.1 | ADAMTS5 | 340001 | 380000 | 0.350 | 1.612 |
| KB743139.1 | LRP3 | 720001 | 760000 | 0.350 | 1.053 |
| KB743256.1 | AREL1/YLPM1/FCF1 | 1120001 | 1160000 | 0.350 | 4.237 |
| KB742617.1 | FGF6 | 1460001 | 1500000 | 0.349 | 1.520 |
| KB743543.1 | NID1 | 460001 | 500000 | 0.347 | 1.829 |
| KB742969.1 | STX2 | 1560001 | 1600000 | 0.345 | 1.111 |
| KB743670.1 | SMOC2 | 1920001 | 1960000 | 0.344 | 1.272 |
| KB742543.1 | ADAMTS5 | 360001 | 400000 | 0.344 | 1.892 |
| KB742984.1 | CMIP | 3320001 | 3360000 | 0.344 | 0.876 |
| KB743139.1 | CEP89/FAAP24 | 600001 | 640000 | 0.342 | 1.055 |
| KB743040.1 | PSME4 | 2480001 | 2520000 | 0.342 | 1.585 |
| KB743139.1 | SLC7A10 | 760001 | 800000 | 0.342 | 1.530 |
| KB743073.1 | PAPPA2 | 940001 | 980000 | 0.341 | 2.744 |
| KB743177.1 | IGF2BP3 | 1300001 | 1340000 | 0.340 | 2.390 |
| KB743177.1 | IGF2BP3 | 1280001 | 1320000 | 0.339 | 2.301 |
| KB744573.1 | ENSAPLT00000006531 | 1 | 40000 | 0.338 | 1.139 |
| KB742446.1 | ENSAPLT00000006207 | 860001 | 900000 | 0.338 | 1.162 |
| KB743364.1 | REEP2 | 1240001 | 1280000 | 0.338 | 2.053 |
| KB742682.1 | SLC5A3 | 400001 | 440000 | 0.335 | 1.122 |
| KB742446.1 | LAMC1 | 600001 | 640000 | 0.333 | 1.456 |
| KB742432.1 | MED23 | 660001 | 700000 | 0.333 | 1.163 |
| KB742685.1 | ADCK1 | 1400001 | 1440000 | 0.333 | 1.875 |
| KB742939.1 | ZCRB1 | 420001 | 460000 | 0.333 | 1.775 |
| KB742815.1 | UTRN | 260001 | 300000 | 0.332 | 1.898 |
| KB745111.1 | ENSAPLT00000008164/ENSAPLT00000008721 | 60001 | 100000 | 0.331 | 2.993 |
| KB742682.1 | MRPS6/SLC5A3 | 380001 | 420000 | 0.330 | 1.120 |
| KB742446.1 | RC3H1 | 780001 | 820000 | 0.330 | 1.534 |
| KB742563.1 | TSPAN2 | 80001 | 120000 | 0.328 | 1.596 |
| KB743543.1 | LYST | 360001 | 400000 | 0.328 | 2.919 |
| KB742432.1 | MOXD1 | 240001 | 280000 | 0.326 | 2.056 |
| KB742815.1 | UTRN | 240001 | 280000 | 0.325 | 2.129 |
| KB742969.1 | GLT1D1 | 880001 | 920000 | 0.325 | 1.503 |
| KB742430.1 | SPATA17 | 820001 | 860000 | 0.325 | 2.084 |
| KB745111.1 | ENSAPLT00000006993 | 1 | 40000 | 0.324 | 2.782 |
| KB743543.1 | NID1 | 440001 | 480000 | 0.324 | 2.551 |
| KB742617.1 | ENSAPLT00000003051 | 1380001 | 1420000 | 0.324 | 2.040 |
| KB743464.1 | SUCLG1 | 440001 | 480000 | 0.323 | 3.008 |
| KB743546.1 | PREP | 280001 | 320000 | 0.323 | 2.066 |
| KB743625.1 | NUP205 | 1340001 | 1380000 | 0.322 | 1.233 |
| KB742648.1 | ENSAPLT00000016377 | 320001 | 360000 | 0.321 | 2.938 |
| KB742386.1 | MTHFD1L | 980001 | 1020000 | 0.320 | 1.272 |
| KB743153.1 | CCSAP/NUP133/ENSAPLT00000004261 | 1520001 | 1560000 | 0.320 | 1.309 |
| KB743139.1 | PEPD | 1060001 | 1100000 | 0.319 | 2.000 |
| KB742540.1 | ENSAPLT00000014637/RAB43 | 720001 | 760000 | 0.319 | 2.109 |
| KB742969.1 | SLC15A4 | 820001 | 860000 | 0.318 | 1.977 |
| KB742781.1 | THADA | 1220001 | 1260000 | 0.318 | 1.687 |
| KB742984.1 | CENPNk/ATMIN/CMC2 | 3100001 | 3140000 | 0.318 | 1.285 |
| KB745111.1 | ENSAPLT00000008721 | 80001 | 120000 | 0.317 | 2.854 |
| KB742648.1 | ENSAPLT00000016377 | 300001 | 340000 | 0.317 | 2.750 |
| KB742567.1 | RTTN | 480001 | 520000 | 0.316 | 1.042 |
| KB742939.1 | PPHLN1 | 380001 | 420000 | 0.316 | 0.960 |
| KB743364.1 | KDM3B/REEP2 | 1220001 | 1260000 | 0.315 | 2.411 |
| KB742685.1 | ENSAPLT00000016920 | 1600001 | 1640000 | 0.315 | 1.898 |
| KB742969.1 | SLC15A4/GLT1D1 | 860001 | 900000 | 0.315 | 1.830 |
| KB743073.1 | ENSAPLT00000003613 | 1300001 | 1340000 | 0.314 | 1.503 |
| KB743543.1 | LYST | 340001 | 380000 | 0.313 | 3.768 |
| KB742606.1 | ENSAPLT00000007138 | 280001 | 320000 | 0.313 | 0.958 |
| KB744033.1 | PATZ1/PIK3IP1 | 880001 | 920000 | 0.312 | 3.047 |
| KB742969.1 | GLT1D1 | 900001 | 940000 | 0.311 | 1.818 |
| KB743050.1 | NRCAM | 620001 | 660000 | 0.311 | 2.839 |
| KB745111.1 | ENSAPLT00000008164 | 40001 | 80000 | 0.310 | 2.757 |
| KB748162.1 | WFDC1 | 120001 | 160000 | 0.309 | 1.344 |
| KB742984.1 | ATMIN/GCSH | 3120001 | 3160000 | 0.309 | 1.067 |
| KB742481.1 | MMP27/ENSAPLT00000006239 | 700001 | 740000 | 0.308 | 2.036 |
| KB742617.1 | ENSAPLT00000003051 | 1360001 | 1400000 | 0.308 | 1.703 |
| KB742567.1 | RTTN | 460001 | 500000 | 0.307 | 0.982 |
| KB742386.1 | ENSAPLT00000012286 | 1100001 | 1140000 | 0.307 | 2.237 |
| KB742617.1 | ENSAPLT00000003051/TIGAR | 1400001 | 1440000 | 0.306 | 2.217 |
| KB742432.1 | MED23 | 680001 | 720000 | 0.306 | 1.502 |
| KB748162.1 | ATP2C2/WFDC1 | 100001 | 140000 | 0.306 | 1.111 |
| KB744033.1 | LIMK2/PIK3IP1 | 860001 | 900000 | 0.306 | 2.458 |
| KB744033.1 | EIF4ENIF/SFI1/DRG1 | 920001 | 960000 | 0.306 | 2.156 |
| KB742939.1 | PPHLN1/ZCRB1 | 400001 | 440000 | 0.306 | 1.268 |
| KB743543.1 | NID1 | 480001 | 520000 | 0.306 | 1.765 |
| KB743073.1 | ASTN1/PAPPA2 | 920001 | 960000 | 0.305 | 2.723 |
| KB743050.1 | NRCAM | 580001 | 620000 | 0.305 | 3.225 |
| KB745378.1 | TDP1 | 1 | 40000 | 0.305 | 1.480 |
| KB742540.1 | ENSAPLT00000014747/ENSAPLT00000014637/CNBP | 740001 | 780000 | 0.304 | 1.125 |
| KB742432.1 | ENPP1 | 540001 | 580000 | 0.303 | 1.412 |
| KB742815.1 | UTRN | 340001 | 380000 | 0.302 | 1.138 |
| KB742668.1 | MTX2 | 520001 | 560000 | 0.302 | 1.864 |
| KB743950.1 | CDH19 | 140001 | 180000 | 0.302 | 1.056 |
| KB742543.1 | ADAMTS5 | 380001 | 420000 | 0.301 | 1.463 |
| KB743316.1 | ROBO2 | 960001 | 1000000 | 0.301 | 1.103 |
| KB742563.1 | TRIM33/DENND2C/BCAS2 | 240001 | 280000 | 0.301 | 1.169 |
| KB742471.1 | ENSAPLT00000017077/TRMU/ENSAPLT00000017064 | 1940001 | 1980000 | 0.300 | 2.004 |
| KB742563.1 | TSPAN2/SYCP1/TSHB | 100001 | 140000 | 0.300 | 1.841 |
| KB743050.1 | NRCAM | 600001 | 640000 | 0.300 | 3.025 |
| KB742992.1 | PROSER1/NHLRC3 | 1 | 40000 | 0.299 | 1.318 |
| KB742617.1 | GABARAPL1/TMEM52B | 1820001 | 1860000 | 0.299 | 2.601 |
| KB742617.1 | GABARAPL1/TMEM52B/ENSAPLT00000001223 | 1840001 | 1880000 | 0.299 | 2.569 |
| KB745378.1 | KCNK13 | 80001 | 120000 | 0.298 | 1.563 |
| KB742984.1 | PLCG2 | 3440001 | 3480000 | 0.297 | 3.735 |
| KB742504.1 | SMS | 640001 | 680000 | 0.297 | 2.513 |
| KB742433.1 | PCDH10 | 1960001 | 2000000 | 0.296 | 1.117 |
| KB742424.1 | CDH2 | 1640001 | 1680000 | 0.296 | 2.942 |
| KB742781.1 | PLEKHH2 | 1100001 | 1140000 | 0.296 | 2.891 |
| KB742386.1 | ENSAPLT00000010356 | 660001 | 700000 | 0.295 | 1.550 |
| KB743139.1 | PEPD | 1040001 | 1080000 | 0.295 | 1.093 |
| KB742969.1 | RIMBP2 | 1440001 | 1480000 | 0.295 | 1.625 |
| KB742539.1 | PITRM1/PFKP | 520001 | 560000 | 0.294 | 2.447 |
| KB743013.1 | PALLD/CBR4 | 240001 | 280000 | 0.294 | 1.733 |
| KB742969.1 | TMEM132C | 760001 | 800000 | 0.293 | 1.538 |
| KB743543.1 | LYST/GNG4 | 320001 | 360000 | 0.293 | 2.425 |
| KB743177.1 | KLHL7 | 1140001 | 1180000 | 0.293 | 1.201 |
| KB742969.1 | TMEM132D | 960001 | 1000000 | 0.292 | 1.733 |
| KB742781.1 | THADA | 1240001 | 1280000 | 0.292 | 1.365 |
| KB742617.1 | C12orf4/FGF6 | 1480001 | 1520000 | 0.291 | 1.575 |
| KB742540.1 | RAF1/MKRN2/TSEN2 | 880001 | 920000 | 0.291 | 2.234 |
| KB743464.1 | SUCLG1 | 420001 | 460000 | 0.291 | 1.815 |
| KB742504.1 | SMS/PHEX | 620001 | 660000 | 0.291 | 2.120 |
| KB742444.1 | SRBD1 | 260001 | 300000 | 0.290 | 1.065 |
| KB742685.1 | ENSAPLT00000016920 | 1520001 | 1560000 | 0.290 | 1.825 |
| KB744936.1 | SRD5A2 | 140001 | 180000 | 0.290 | 2.102 |
| KB743050.1 | PNPLA8 | 500001 | 540000 | 0.289 | 2.832 |
| KB742444.1 | SRBD1 | 240001 | 280000 | 0.289 | 0.953 |
| KB743625.1 | UTP20/SLC5A8 | 1220001 | 1260000 | 0.288 | 1.842 |
| KB742479.1 | PFDN4 | 3420001 | 3460000 | 0.288 | 1.329 |
| KB742540.1 | RAB43/GP9 | 700001 | 740000 | 0.287 | 1.881 |
| KB743625.1 | UTP20 | 1240001 | 1280000 | 0.287 | 2.928 |
| KB742386.1 | MTHFD1L/ENSAPLT00000012286 | 1080001 | 1120000 | 0.286 | 2.096 |
| KB742597.1 | ENSAPLT00000003658/PHC1/M6PR/ENSAPLT00000001984 | 440001 | 480000 | 0.286 | 2.791 |
| KB742386.1 | PLEKHG1/MTHFD1L | 960001 | 1000000 | 0.285 | 1.417 |
| KB742969.1 | RIMBP2 | 1460001 | 1500000 | 0.285 | 1.636 |
| KB743625.1 | UTP20 | 1260001 | 1300000 | 0.284 | 2.052 |
| KB742523.1 | MEF2A | 660001 | 700000 | 0.284 | 1.119 |
| KB742432.1 | ENPP3/MED23 | 620001 | 660000 | 0.283 | 1.002 |
| KB742815.1 | PARK2 | 1600001 | 1640000 | 0.283 | 1.739 |
| KB742617.1 | TIGAR/FGF23 | 1420001 | 1460000 | 0.282 | 1.692 |
| KB743073.1 | PAPPA2 | 960001 | 1000000 | 0.282 | 2.300 |
| KB743256.1 | AREL1/ISCA2 | 1100001 | 1140000 | 0.282 | 2.912 |
| KB745111.1 | ENSAPLT00000008164/ENSAPLT00000006993 | 20001 | 60000 | 0.282 | 2.819 |
| KB745111.1 | ENSAPLT00000008721 | 100001 | 140000 | 0.281 | 2.445 |
| KB743139.1 | SLC7A9/CEP89 | 580001 | 620000 | 0.281 | 2.073 |
| KB747079.1 | ADGRL2 | 100001 | 140000 | 0.281 | 0.945 |
| KB743050.1 | NRCAM | 640001 | 680000 | 0.281 | 2.916 |
| KB742967.1 | MMP24 | 1280001 | 1320000 | 0.280 | 1.343 |
| KB742969.1 | TMEM132B | 60001 | 100000 | 0.280 | 0.983 |
| KB743625.1 | UTP20/NUP205/ARL1 | 1280001 | 1320000 | 0.279 | 1.850 |
| KB742969.1 | SLC15A4 | 840001 | 880000 | 0.279 | 2.170 |
| KB742432.1 | STX7/TAAR5 | 160001 | 200000 | 0.279 | 1.818 |
| KB743073.1 | TNR | 1220001 | 1260000 | 0.279 | 1.358 |
| KB743543.1 | GPR137B/NID1 | 500001 | 540000 | 0.279 | 1.353 |
| KB745378.1 | KCNK13 | 100001 | 140000 | 0.279 | 1.453 |
| KB742504.1 | PHEX | 600001 | 640000 | 0.278 | 1.806 |
| KB742811.1 | STX1A | 60001 | 100000 | 0.278 | 1.213 |
| KB742539.1 | PITRM1 | 540001 | 580000 | 0.278 | 2.093 |
| KB743464.1 | RPIA | 460001 | 500000 | 0.277 | 1.950 |
| KB742565.1 | SLC4A4 | 860001 | 900000 | 0.277 | 1.220 |
| KB742951.1 | GRIK1 | 520001 | 560000 | 0.277 | 1.185 |
| KB743913.1 | ENSAPLT00000013710/ENSAPLT00000013659 | 80001 | 120000 | 0.277 | 1.657 |
| KB743153.1 | PSMB1 | 800001 | 840000 | 0.277 | 1.056 |
| KB743073.1 | ENSAPLT00000003613/CACYBP/MRPS14 | 1260001 | 1300000 | 0.277 | 1.145 |
| KB742504.1 | SMS | 660001 | 700000 | 0.276 | 1.813 |
| KB743050.1 | PNPLA8 | 460001 | 500000 | 0.276 | 1.839 |
| KB745378.1 | TDP1 | 20001 | 60000 | 0.276 | 1.500 |
| KB742617.1 | STYK1/GABARAPL1 | 1800001 | 1840000 | 0.275 | 2.442 |
| KB742386.1 | MTHFD1L | 1000001 | 1040000 | 0.274 | 1.500 |
| KB742716.1 | ZMYND12/SLC2A1/ENSAPLT00000005992 | 360001 | 400000 | 0.274 | 1.163 |
| KB742617.1 | STYK1/YBX3 | 1780001 | 1820000 | 0.274 | 1.288 |
| KB742424.1 | CDH2 | 1620001 | 1660000 | 0.274 | 1.932 |
| KB743336.1 | CRIM1 | 320001 | 360000 | 0.274 | 1.246 |
| KB743913.1 | MARCH6 | 120001 | 160000 | 0.273 | 1.901 |
| KB742685.1 | ENSAPLT00000016920 | 1620001 | 1660000 | 0.272 | 1.575 |
| KB742992.1 | ENSAPLT00000004236/PROSER1/STOML3 | 40001 | 80000 | 0.272 | 1.547 |
| KB742563.1 | SYCP1/ENSAPLT00000008665/ENSAPLT00000009052 | 140001 | 180000 | 0.272 | 1.335 |
| KB743139.1 | PEPD | 1080001 | 1120000 | 0.272 | 1.193 |
| KB742446.1 | ENSAPLT00000006207 | 880001 | 920000 | 0.271 | 1.465 |
| KB744190.1 | T brachyury transcription factor | 60001 | 100000 | 0.271 | 0.925 |
| KB742815.1 | UTRN | 360001 | 400000 | 0.271 | 1.448 |
| KB742969.1 | TMEM132C | 700001 | 740000 | 0.271 | 1.119 |
| KB744033.1 | SFI1\EIF4ENIF1 | 940001 | 980000 | 0.270 | 1.848 |
| KB742504.1 | PHEX | 580001 | 620000 | 0.269 | 1.372 |
| KB744148.1 | ELAVL4 | 260001 | 300000 | 0.269 | 1.169 |
| KB743336.1 | CRIM1 | 300001 | 340000 | 0.269 | 1.473 |
| KB742977.1 | FAM84B | 540001 | 580000 | 0.269 | 1.385 |
| KB742781.1 | DYNC2LI1/PLEKHH2 | 1080001 | 1120000 | 0.269 | 2.004 |
| KB742606.1 | ENSAPLT00000007138 | 300001 | 340000 | 0.269 | 1.048 |
| KB743525.1 | MED12L/P2RY12 /P2RY13/GPR87 | 520001 | 560000 | 0.268 | 1.037 |
| KB743070.1 | SFT2D1 | 440001 | 480000 | 0.268 | 1.081 |
| KB743336.1 | CRIM1/FEZ2 | 280001 | 320000 | 0.267 | 1.663 |
| KB742887.1 | DERA | 2680001 | 2720000 | 0.266 | 1.707 |
| KB743546.1 | PREP | 260001 | 300000 | 0.266 | 1.555 |
| KB742685.1 | ENSAPLT00000016920 | 1580001 | 1620000 | 0.266 | 1.929 |
| KB743050.1 | PNPLA8/AVPR2 | 440001 | 480000 | 0.266 | 1.458 |
| KB742931.1 | ENSAPLT00000010078 | 4000001 | 4040000 | 0.266 | 1.105 |
| KB744486.1 | EFCAB11 | 660001 | 700000 | 0.265 | 0.879 |
| KB743177.1 | IGF2BP3 | 1260001 | 1300000 | 0.265 | 1.416 |
| KB743625.1 | ENSAPLT00000011897 | 680001 | 720000 | 0.265 | 1.021 |
| KB743153.1 | CCSAP/NUP133/ABCB10 | 1500001 | 1540000 | 0.264 | 1.495 |
| KB742619.1 | KIT | 640001 | 680000 | 0.264 | 0.862 |
| KB742951.1 | MAP3K7CL | 720001 | 760000 | 0.264 | 1.257 |
| KB742815.1 | UTRN | 280001 | 320000 | 0.263 | 1.365 |
| KB743197.1 | BEGAIN | 1520001 | 1560000 | 0.263 | 1.280 |
| KB744033.1 | HNF1A/ENSAPLT00000002965 | 800001 | 840000 | 0.263 | 1.865 |
| KB744148.1 | ELAVL4 | 240001 | 280000 | 0.263 | 1.216 |
| KB742446.1 | RC3H1 | 760001 | 800000 | 0.263 | 1.781 |
| KB742887.1 | LMO3 | 2440001 | 2480000 | 0.263 | 1.112 |
| KB742969.1 | TMEM132B | 40001 | 80000 | 0.263 | 0.943 |
| KB742951.1 | MAP3K7CL | 700001 | 740000 | 0.263 | 0.926 |
| KB743153.1 | ENSAPLT00000014084/ENSAPLT00000014050 | 880001 | 920000 | 0.261 | 0.878 |
| KB742565.1 | SLC4A4 | 840001 | 880000 | 0.261 | 1.152 |
| KB742781.1 | THADA | 1200001 | 1240000 | 0.261 | 1.855 |
| KB742685.1 | ENSAPLT00000016920 | 1700001 | 1740000 | 0.261 | 1.770 |
| KB742697.1 | UBE2V1 | 180001 | 220000 | 0.261 | 1.030 |
| KB743336.1 | FEZ2 | 260001 | 300000 | 0.261 | 1.649 |
| KB743610.1 | MAPKAP1 | 400001 | 440000 | 0.260 | 1.815 |
| KB743110.1 | OSBPL5 | 1820001 | 1860000 | 0.260 | 1.001 |
| KB743922.1 | UBE2Z/CALCOCO2/SNF8 /ATP5G1 | 120001 | 160000 | 0.260 | 0.953 |
| KB742808.1 | ZCCHC24 | 4600001 | 4640000 | 0.260 | 1.368 |
| KB742781.1 | THADA | 1260001 | 1300000 | 0.259 | 1.293 |
| KB742540.1 | ENSAPLT00000014546 | 660001 | 700000 | 0.258 | 1.939 |
| KB742540.1 | RAF1 | 840001 | 880000 | 0.257 | 1.692 |
| KB742386.1 | PCMT1/NUP43/LATS1 | 540001 | 580000 | 0.257 | 0.941 |
| KB744195.1 | SCRG1 | 920001 | 960000 | 0.257 | 1.105 |
| KB742853.1 | FAM198B | 1060001 | 1100000 | 0.257 | 0.860 |
| KB742697.1 | UBE2V1 | 160001 | 200000 | 0.256 | 1.443 |
| KB742760.1 | TULP4 | 200001 | 240000 | 0.256 | 1.404 |
| KB742617.1 | YBX3 | 1760001 | 1800000 | 0.256 | 1.000 |
| KB744266.1 | HP1BP3/KIF17/SH2D5 | 240001 | 280000 | 0.256 | 1.211 |
| KB742931.1 | ENSAPLT00000010078/TMX3 | 4020001 | 4060000 | 0.256 | 1.347 |
| KB743891.1 | CCDC174/ENSAPLT00000010716/ENSAPLT00000000961 | 220001 | 260000 | 0.255 | 0.972 |
| KB742540.1 | RAF1 | 860001 | 900000 | 0.255 | 2.161 |
| KB744976.1 | DCHS1 | 120001 | 160000 | 0.255 | 0.990 |
| KB742840.1 | ENSAPLT00000014215 | 920001 | 960000 | 0.255 | 0.956 |
| KB742444.1 | SRBD1 | 280001 | 320000 | 0.255 | 0.859 |
| KB743045.1 | LRP2 | 1460001 | 1500000 | 0.255 | 1.152 |
| KB742551.1 | FASN | 580001 | 620000 | 0.255 | 1.449 |
| KB744741.1 | KIF26A | 40001 | 80000 | 0.255 | 1.279 |
| KB742523.1 | ST8SIA2 | 3360001 | 3400000 | 0.254 | 1.455 |
| KB744477.1 | COPB2/MRPS22 | 440001 | 480000 | 0.254 | 1.895 |
| KB743336.1 | ENSAPLT00000012625/FEZ2 | 240001 | 280000 | 0.253 | 1.737 |
| KB742563.1 | DENND2C/AMPD1/BCAS2/TRIM33 | 220001 | 260000 | 0.253 | 1.314 |
| KB743402.1 | CCND1 | 800001 | 840000 | 0.252 | 1.908 |
| KB743045.1 | ENSAPLT00000007569/ENSAPLT00000007300/KLHL41 | 1380001 | 1420000 | 0.251 | 1.336 |
| KB742404.1 | C16orf72 | 2500001 | 2540000 | 0.251 | 0.873 |
| KB743534.1 | GRM8 | 120001 | 160000 | 0.251 | 1.082 |
| KB742781.1 | PLEKHH2/THADA | 1140001 | 1180000 | 0.251 | 2.307 |
| KB742432.1 | MOXD1 | 260001 | 300000 | 0.250 | 2.387 |
| KB742969.1 | TMEM132C | 720001 | 760000 | 0.250 | 1.397 |
| KB743073.1 | ENSAPLT00000003613 | 1320001 | 1360000 | 0.250 | 1.091 |
| KB742539.1 | PFKP/PITRM1 | 500001 | 540000 | 0.250 | 2.831 |
| KB743256.1 | YLPM1/FCF1 | 1140001 | 1180000 | 0.250 | 1.729 |
| KB742617.1 | ENSAPLT00000003266/ENSAPLT00000001223 | 1860001 | 1900000 | 0.249 | 2.482 |
| KB743810.1 | SLC35C2 | 140001 | 180000 | 0.249 | 1.430 |
| KB742833.1 | FGFRL1 | 380001 | 420000 | 0.248 | 1.171 |
| KB742877.1 | ELOVL2 | 520001 | 560000 | 0.248 | 0.956 |
| KB742685.1 | ENSAPLT00000016920 | 1760001 | 1800000 | 0.248 | 2.396 |
| KB742597.1 | PHC1/M6PR | 420001 | 460000 | 0.248 | 2.211 |
| KB743364.1 | KDM3B | 1200001 | 1240000 | 0.247 | 2.076 |
| KB742406.1 | TINAG | 540001 | 580000 | 0.247 | 0.866 |
| KB746343.1 | ENSAPLT00000001840 | 260001 | 300000 | 0.247 | 2.558 |
| KB744799.1 | RASSF3 | 140001 | 180000 | 0.247 | 1.318 |
| KB743139.1 | LRP3/WDR88 | 700001 | 740000 | 0.247 | 0.852 |
| KB743050.1 | PNPLA8 | 480001 | 520000 | 0.246 | 2.738 |
| KB743204.1 | MAPK11 | 2160001 | 2200000 | 0.246 | 1.146 |
| KB744486.1 | FOXN3 | 520001 | 560000 | 0.246 | 1.046 |
| KB744198.1 | GRIN2A | 760001 | 800000 | 0.246 | 1.316 |
| KB742386.1 | MTHFD1L | 1020001 | 1060000 | 0.246 | 1.718 |
| KB743153.1 | GNPAT/ENSAPLT00000014084/ENSAPLT00000014050 | 860001 | 900000 | 0.246 | 1.373 |
| KB742540.1 | GP9 | 680001 | 720000 | 0.245 | 1.654 |
| KB742685.1 | ADCK1 | 1380001 | 1420000 | 0.245 | 1.585 |
| KB744033.1 | SFI1\PISD | 960001 | 1000000 | 0.244 | 1.546 |
| KB743692.1 | ENSAPLT00000013461 | 260001 | 300000 | 0.244 | 1.047 |
| KB743336.1 | ENSAPLT00000012625 | 220001 | 260000 | 0.244 | 1.501 |
| KB742479.1 | ENSAPLT00000002724/ANKRD60 | 1940001 | 1980000 | 0.244 | 2.351 |
| KB743740.1 | STAB1 | 1040001 | 1080000 | 0.244 | 1.524 |
| KB742760.1 | TULP4 | 180001 | 220000 | 0.244 | 1.107 |
| KB742432.1 | MOXD1 | 280001 | 320000 | 0.243 | 2.198 |
| KB744477.1 | PER2 | 820001 | 860000 | 0.243 | 2.519 |
| KB744518.1 | RANBP10 | 20001 | 60000 | 0.243 | 1.342 |
| KB742957.1 | ITGA8 | 100001 | 140000 | 0.242 | 1.102 |
| KB742617.1 | ENSAPLT00000003266 | 1880001 | 1920000 | 0.242 | 2.752 |
| KB742969.1 | TMEM132C | 740001 | 780000 | 0.242 | 1.378 |
| KB742650.1 | ENSAPLT00000012039 | 220001 | 260000 | 0.242 | 0.849 |
| KB742716.1 | PPIH/CCDC30/YBX1 | 300001 | 340000 | 0.242 | 1.345 |
| KB743111.1 | SPTLC3 | 980001 | 1020000 | 0.242 | 1.082 |
| KB742619.1 | KIT | 620001 | 660000 | 0.241 | 1.326 |
| KB742386.1 | ENSAPLT00000010342 | 600001 | 640000 | 0.241 | 1.816 |
| KB743158.1 | RAB3GAP1/ZRANB3/ENSAPLT00000001872 | 2100001 | 2140000 | 0.241 | 1.142 |
| KB743525.1 | MED12L/P2RY12/P2RY13 | 540001 | 580000 | 0.241 | 1.244 |
| KB744699.1 | NRTN/ENSAPLT00000001423 | 400001 | 440000 | 0.240 | 0.960 |
| KB743625.1 | UHRF1BP1L | 720001 | 760000 | 0.240 | 1.080 |
| KB744477.1 | PER2 | 800001 | 840000 | 0.240 | 2.576 |
| KB742967.1 | MMP24 | 1260001 | 1300000 | 0.240 | 0.972 |
| KB743197.1 | RCOR1 | 400001 | 440000 | 0.240 | 1.208 |
| KB742716.1 | ZMYND12/CCDC30/ENSAPLT00000005992 | 340001 | 380000 | 0.239 | 1.516 |
| KB742444.1 | SRBD1 | 220001 | 260000 | 0.239 | 1.296 |
| KB742647.1 | IMMP2L | 460001 | 500000 | 0.239 | 2.885 |
| KB743818.1 | ZDHHC14 | 20001 | 60000 | 0.239 | 1.331 |
| KB742647.1 | IMMP2L | 440001 | 480000 | 0.239 | 1.760 |
| KB742479.1 | C20orf85/ANKRD60 | 1960001 | 2000000 | 0.238 | 1.181 |
| KB742887.1 | LMO3 | 2500001 | 2540000 | 0.238 | 2.066 |
| KB743534.1 | GRM8 | 40001 | 80000 | 0.238 | 1.216 |
| KB742565.1 | SLC4A4 | 880001 | 920000 | 0.238 | 1.179 |
| KB742931.1 | TMX3 | 4040001 | 4080000 | 0.237 | 1.357 |
| KB743670.1 | ENSAPLT00000001135 | 240001 | 280000 | 0.237 | 1.168 |
| KB742781.1 | THADA | 1160001 | 1200000 | 0.237 | 2.016 |
| KB743740.1 | NT5DC2/STAB1 | 1020001 | 1060000 | 0.237 | 1.386 |
| KB742685.1 | STON2 | 2920001 | 2960000 | 0.236 | 1.415 |
| KB742815.1 | UTRN | 320001 | 360000 | 0.236 | 0.978 |
| KB742951.1 | GRIK1 | 540001 | 580000 | 0.235 | 1.312 |
| KB742969.1 | TMEM132D | 940001 | 980000 | 0.235 | 1.837 |
| KB742833.1 | AFAP1 | 5080001 | 5120000 | 0.234 | 1.026 |
| KB743158.1 | ZRANB3/RAB3GAP1 | 2120001 | 2160000 | 0.234 | 1.436 |
| KB742424.1 | CDH2 | 1600001 | 1640000 | 0.234 | 1.399 |
| KB742523.1 | MEF2A | 680001 | 720000 | 0.233 | 0.982 |
| KB743073.1 | TNN/CACYBP/MRPS14 | 1240001 | 1280000 | 0.233 | 1.056 |
| KB742432.1 | ENPP1 | 560001 | 600000 | 0.233 | 1.201 |
| KB743810.1 | ELMO2/SLC35C2 | 120001 | 160000 | 0.232 | 1.491 |
| KB742815.1 | UTRN | 100001 | 140000 | 0.232 | 1.026 |
| KB742682.1 | KCNE2 | 220001 | 260000 | 0.232 | 0.928 |
| KB743402.1 | CCND1/FGF19/ORAOV1 | 820001 | 860000 | 0.232 | 1.809 |
| KB742471.1 | PIK3CG | 580001 | 620000 | 0.232 | 1.442 |
| KB743111.1 | ENSAPLT00000012919 | 1320001 | 1360000 | 0.231 | 0.861 |
| KB742781.1 | PPM1B | 820001 | 860000 | 0.231 | 0.907 |
| KB742551.1 | FASN/DUS1L | 560001 | 600000 | 0.231 | 1.212 |
| KB743158.1 | THSD7B | 2660001 | 2700000 | 0.231 | 1.042 |
| KB742386.1 | IYD | 760001 | 800000 | 0.231 | 0.881 |
| KB743139.1 | COQ9/CIAPIN1/POLR2C/ENSAPLT00000006564 | 540001 | 580000 | 0.231 | 1.316 |
| KB743670.1 | SMOC2 | 1940001 | 1980000 | 0.230 | 1.184 |
| KB744976.1 | LCMT2 | 140001 | 180000 | 0.230 | 1.392 |
| KB743103.1 | LRRC3/SAMD7/LRRIQ4 | 40001 | 80000 | 0.230 | 1.734 |
| KB742890.1 | ENSAPLT00000002925 | 160001 | 200000 | 0.229 | 0.861 |
| KB742404.1 | USP7 | 2360001 | 2400000 | 0.229 | 0.944 |
| KB742969.1 | GLT1D1 | 920001 | 960000 | 0.229 | 1.722 |
| KB743818.1 | ZDHHC14 | 40001 | 80000 | 0.228 | 1.329 |
| KB743158.1 | THSD7B | 2680001 | 2720000 | 0.228 | 1.666 |
| KB743162.1 | NEDD1 | 20001 | 60000 | 0.228 | 0.976 |
| KB744195.1 | ENSAPLT00000003336/SCRG1 | 940001 | 980000 | 0.228 | 1.222 |
| KB742504.1 | PHEX | 560001 | 600000 | 0.228 | 0.975 |
| KB744198.1 | GRIN2A | 740001 | 780000 | 0.228 | 1.121 |
| KB742685.1 | ENSAPLT00000016920 | 1540001 | 1580000 | 0.227 | 1.565 |
| KB742444.1 | SRBD1 | 180001 | 220000 | 0.227 | 1.453 |
| KB743402.1 | FGF19/ORAOV1 | 840001 | 880000 | 0.227 | 2.093 |
| KB743534.1 | GRM8 | 140001 | 180000 | 0.227 | 1.616 |
| KB743070.1 | SFT2D1/MPC1 | 420001 | 460000 | 0.227 | 1.267 |
| KB742386.1 | MTHFD1L | 1040001 | 1080000 | 0.227 | 1.608 |
| KB743197.1 | TRAF3 | 280001 | 320000 | 0.226 | 1.858 |
| KB743050.1 | AVPR2 | 420001 | 460000 | 0.226 | 1.614 |
| KB743110.1 | OSBPL5 | 1860001 | 1900000 | 0.226 | 1.070 |
| KB743050.1 | NRCAM | 660001 | 700000 | 0.226 | 1.427 |
| KB744663.1 | C1orf27/PDC | 200001 | 240000 | 0.226 | 1.009 |
| KB742479.1 | ENSAPLT00000001285 | 1700001 | 1740000 | 0.226 | 0.972 |
| KB742597.1 | ENSAPLT00000003658/ENSAPLT00000001984 | 460001 | 500000 | 0.225 | 1.301 |
| KB744088.1 | VPS13B | 1740001 | 1780000 | 0.225 | 1.006 |
| KB742471.1 | TRMU/ENSAPLT00000017077 | 1960001 | 2000000 | 0.225 | 1.196 |
| KB742887.1 | DERA | 2700001 | 2740000 | 0.225 | 1.296 |
| KB742481.1 | MMP7 | 640001 | 680000 | 0.224 | 1.957 |
| KB743197.1 | EVL | 1760001 | 1800000 | 0.224 | 1.772 |
| KB743045.1 | FASTKD1/KLHL41 | 1360001 | 1400000 | 0.224 | 2.492 |
| KB742957.1 | ITGA8/FAM188A | 80001 | 120000 | 0.224 | 1.009 |
| KB743633.1 | ENSAPLT00000008016 | 520001 | 560000 | 0.224 | 1.164 |
| KB743197.1 | EVL | 1820001 | 1860000 | 0.223 | 1.559 |
| KB743073.1 | ENSAPLT00000003613 | 1360001 | 1400000 | 0.223 | 1.558 |
| KB742781.1 | PLEKHH2 | 1120001 | 1160000 | 0.223 | 2.700 |
| KB742781.1 | DYNC2LI1/ABCG5/ABCG8 | 1060001 | 1100000 | 0.223 | 1.683 |
| KB742969.1 | ENSAPLT00000001260 | 540001 | 580000 | 0.223 | 0.869 |
| KB742853.1 | TMA16 | 2780001 | 2820000 | 0.223 | 1.014 |
| KB743110.1 | OSBPL5 | 1840001 | 1880000 | 0.223 | 1.110 |
| KB743235.1 | EYA1 | 1320001 | 1360000 | 0.223 | 1.046 |
| KB742490.1 | ENSAPLT00000011916 | 1560001 | 1600000 | 0.223 | 1.308 |
| KB742432.1 | EPB41L2 | 980001 | 1020000 | 0.223 | 0.940 |
| KB743073.1 | RFWD2 | 1040001 | 1080000 | 0.222 | 1.018 |
| KB743013.1 | PALLD | 260001 | 300000 | 0.222 | 1.462 |
| KB743735.1 | ELOVL4 | 40001 | 80000 | 0.222 | 1.387 |
| KB742404.1 | ENSAPLT00000016276/ENSAPLT00000000847 | 2640001 | 2680000 | 0.222 | 0.943 |
| KB743534.1 | GRM8 | 360001 | 400000 | 0.222 | 1.159 |
| KB742540.1 | ENSAPLT00000014546 | 640001 | 680000 | 0.222 | 0.922 |
| KB742716.1 | CCDC30 | 320001 | 360000 | 0.222 | 1.508 |
| KB742685.1 | ENSAPLT00000016920 | 1640001 | 1680000 | 0.221 | 1.540 |
| KB742481.1 | ENSAPLT00000005979 | 600001 | 640000 | 0.221 | 1.005 |
| KB746535.1 | SF3A2/ENSAPLT00000011459/ENSAPLT00000011465/OAZ1 | 20001 | 60000 | 0.221 | 1.142 |
| KB743177.1 | IGF2BP3 | 1320001 | 1360000 | 0.221 | 1.045 |
| KB742685.1 | ENSAPLT00000016920 | 1780001 | 1820000 | 0.221 | 2.630 |
| KB742432.1 | ENPP3 | 600001 | 640000 | 0.221 | 1.203 |
| KB743158.1 | ZRANB3 | 2140001 | 2180000 | 0.220 | 1.167 |
| KB743534.1 | GRM8 | 300001 | 340000 | 0.220 | 0.968 |
| KB742406.1 | TINAG | 560001 | 600000 | 0.220 | 0.853 |
| KB742887.1 | DERA/STRAP | 2720001 | 2760000 | 0.220 | 0.909 |
| KB742617.1 | YBX3 | 1740001 | 1780000 | 0.220 | 1.299 |
| KB742383.1 | ENSAPLT00000004961 | 480001 | 520000 | 0.219 | 1.420 |
| KB744033.1 | ENSAPLT00000006670/PISD | 980001 | 1020000 | 0.219 | 0.957 |
| KB742826.1 | CA10 | 500001 | 540000 | 0.219 | 0.929 |
| KB743633.1 | ENSAPLT00000008016 | 500001 | 540000 | 0.219 | 1.018 |
| KB743609.1 | BMPR1B | 1420001 | 1460000 | 0.219 | 1.147 |
| KB743396.1 | ESR2 | 340001 | 380000 | 0.219 | 1.392 |
| KB742815.1 | UTRN | 300001 | 340000 | 0.219 | 1.072 |
| KB744088.1 | VPS13B | 1720001 | 1760000 | 0.219 | 1.268 |
| KB743013.1 | PALLD | 380001 | 420000 | 0.218 | 1.271 |
| KB742826.1 | CA10 | 520001 | 560000 | 0.218 | 0.917 |
| KB742833.1 | RNF212 | 540001 | 580000 | 0.218 | 1.098 |
| KB743692.1 | ENSAPLT00000013461 | 280001 | 320000 | 0.218 | 1.009 |
| KB742430.1 | SPATA17 | 800001 | 840000 | 0.218 | 2.446 |
| KB742853.1 | GRIA2 | 760001 | 800000 | 0.218 | 1.724 |
| KB744088.1 | VPS13B | 1700001 | 1740000 | 0.217 | 1.263 |
| KB743204.1 | ENSAPLT00000003155/TRABD | 2000001 | 2040000 | 0.217 | 0.919 |
| KB742432.1 | AKAP7/ENSAPLT00000000930 | 820001 | 860000 | 0.217 | 0.914 |
| KB743073.1 | ASTN1 | 900001 | 940000 | 0.217 | 2.098 |
| KB742471.1 | TBC1D22A | 2540001 | 2580000 | 0.217 | 2.216 |
| KB743922.1 | CALCOCO2/ATP5G1/UBE2Z | 140001 | 180000 | 0.217 | 0.899 |
| KB742386.1 | PLEKHG1 | 940001 | 980000 | 0.217 | 1.075 |
| KB743103.1 | SEC62/SAMD7 | 60001 | 100000 | 0.217 | 1.330 |
| KB742539.1 | PFKP | 480001 | 520000 | 0.216 | 1.854 |
| KB743950.1 | CDH19 | 160001 | 200000 | 0.216 | 1.405 |
| KB742815.1 | PARK2 | 1460001 | 1500000 | 0.216 | 1.770 |
| KB743435.1 | ENSAPLT00000004541/SLCO1A2 | 2760001 | 2800000 | 0.216 | 1.049 |
| KB743040.1 | ACYP2 | 2460001 | 2500000 | 0.216 | 1.099 |
| KB743050.1 | THAP5/DNAJB9 | 360001 | 400000 | 0.216 | 1.649 |
| KB742597.1 | ENSAPLT00000002446/PHC1 | 400001 | 440000 | 0.216 | 0.921 |
| KB743357.1 | MAML3 | 160001 | 200000 | 0.216 | 1.384 |
| KB743197.1 | EVL | 1800001 | 1840000 | 0.216 | 1.801 |
| KB744477.1 | PER2 | 840001 | 880000 | 0.216 | 2.044 |
| KB743070.1 | MPC1/RPS6KA2 | 360001 | 400000 | 0.216 | 0.886 |
| KB743050.1 | THAP5/DNAJB9 | 380001 | 420000 | 0.215 | 1.210 |
| KB742567.1 | SOCS6 | 580001 | 620000 | 0.215 | 0.979 |
| KB742539.1 | PFKP | 460001 | 500000 | 0.215 | 1.058 |
| KB742563.1 | CSDE1/AMPD1/NRAS | 180001 | 220000 | 0.215 | 0.892 |
| KB742537.1 | PAMR1/ENSAPLT00000012223 | 480001 | 520000 | 0.215 | 0.877 |
| KB742424.1 | CDH2 | 1660001 | 1700000 | 0.215 | 1.695 |
| KB743246.1 | ENSAPLT00000015360 | 1040001 | 1080000 | 0.215 | 1.784 |
| KB743013.1 | PALLD | 400001 | 440000 | 0.215 | 0.994 |
| KB744518.1 | RANBP10 | 1 | 40000 | 0.214 | 1.409 |
| KB742616.1 | ENSAPLT00000012524 | 620001 | 660000 | 0.214 | 0.849 |
| KB742969.1 | TMEM132C | 640001 | 680000 | 0.214 | 1.117 |
| KB742406.1 | TINAG | 520001 | 560000 | 0.214 | 1.121 |
| KB742668.1 | MTX2 | 500001 | 540000 | 0.214 | 1.661 |
| KB742833.1 | SORCS2 | 5220001 | 5260000 | 0.214 | 1.036 |
| KB744292.1 | SVIL | 260001 | 300000 | 0.214 | 1.028 |
| KB744477.1 | COPB2/MRPS22 | 420001 | 460000 | 0.214 | 1.419 |
| KB742479.1 | CYP24A1/PFDN4 | 3440001 | 3480000 | 0.214 | 0.926 |
| KB743256.1 | ENSAPLT00000011627 | 40001 | 80000 | 0.214 | 1.014 |
| KB742815.1 | UTRN | 120001 | 160000 | 0.213 | 1.153 |
| KB742860.1 | GPRC6A/RFX6/FAM162B | 900001 | 940000 | 0.213 | 0.995 |
| KB743070.1 | FGFR1OP/ENSAPLT00000011274 | 40001 | 80000 | 0.213 | 1.229 |
| KB743818.1 | ZDHHC14 | 60001 | 100000 | 0.213 | 1.253 |
| KB743261.1 | GRM1 | 400001 | 440000 | 0.213 | 1.012 |
| KB742730.1 | ENSAPLT00000001355 | 200001 | 240000 | 0.212 | 0.910 |
| KB742815.1 | UTRN | 40001 | 80000 | 0.212 | 1.017 |
| KB743740.1 | STAB1 | 1060001 | 1100000 | 0.212 | 1.207 |
| KB744936.1 | SRD5A2 | 120001 | 160000 | 0.212 | 1.667 |
| KB743197.1 | RCOR1 | 380001 | 420000 | 0.212 | 1.381 |
| KB742685.1 | ENSAPLT00000016920 | 1720001 | 1760000 | 0.212 | 1.991 |
| KB742815.1 | UTRN | 140001 | 180000 | 0.212 | 1.261 |
| KB742386.1 | MTHFD1L | 1060001 | 1100000 | 0.212 | 1.546 |
| KB743103.1 | LRRC34/LRRC31/LRRIQ4 | 20001 | 60000 | 0.212 | 1.068 |
| KB743412.1 | CACHD1 | 60001 | 100000 | 0.212 | 1.026 |
| KB742479.1 | ENSAPLT00000002724/RAB22A | 1920001 | 1960000 | 0.212 | 1.793 |
| KB742619.1 | KIT | 600001 | 640000 | 0.212 | 0.980 |
| KB743197.1 | EVL/DEGS2 | 1740001 | 1780000 | 0.212 | 1.664 |
| KB743178.1 | TATDN1/ENSAPLT00000007406/RNF139/ENSAPLT00000007405 | 420001 | 460000 | 0.212 | 1.181 |
| KB743013.1 | PALLD | 300001 | 340000 | 0.211 | 1.141 |
| KB743543.1 | GNG4/B3GALNT2 | 300001 | 340000 | 0.211 | 1.186 |
| KB742781.1 | THADA | 1280001 | 1320000 | 0.211 | 1.152 |
| KB744518.1 | RANBP10 | 40001 | 80000 | 0.211 | 0.968 |
| KB742977.1 | ENSAPLT00000014574 | 80001 | 120000 | 0.211 | 1.352 |
| KB742815.1 | PARK2 | 1580001 | 1620000 | 0.210 | 1.105 |
| KB743430.1 | CCDC102B | 80001 | 120000 | 0.210 | 1.046 |
| KB743204.1 | MAPK11/MAPK12 | 2140001 | 2180000 | 0.210 | 0.983 |
| KB742931.1 | TMX3 | 4060001 | 4100000 | 0.210 | 1.063 |
| KB743197.1 | EML1 | 1900001 | 1940000 | 0.210 | 0.939 |
| KB746535.1 | SF3A2/ENSAPLT00000011404/ENSAPLT00000011459/ENSAPLT00000011465 | 1 | 40000 | 0.210 | 0.988 |
| KB743610.1 | MAPKAP1 | 420001 | 460000 | 0.210 | 1.082 |
| KB743316.1 | ROBO2 | 980001 | 1020000 | 0.210 | 1.389 |
| KB743226.1 | CACUL1 | 440001 | 480000 | 0.209 | 0.897 |
| KB742481.1 | MMP7 | 620001 | 660000 | 0.209 | 1.619 |
| KB743625.1 | NUP205 | 1320001 | 1360000 | 0.209 | 0.907 |
| KB742619.1 | TMEM165/SRD5A3 | 780001 | 820000 | 0.209 | 1.578 |
| KB742481.1 | ENSAPLT00000005609/ENSAPLT00000005979 | 580001 | 620000 | 0.209 | 1.132 |
| KB743162.1 | NEDD1 | 40001 | 80000 | 0.209 | 0.908 |
| KB742432.1 | EPB41L2 | 960001 | 1000000 | 0.209 | 1.318 |
| KB744198.1 | GRIN2A | 660001 | 700000 | 0.209 | 1.751 |
| KB742840.1 | ENSAPLT00000014215 | 900001 | 940000 | 0.208 | 1.152 |
| KB742675.1 | RELN | 100001 | 140000 | 0.208 | 1.316 |
| KB742760.1 | TULP4 | 220001 | 260000 | 0.208 | 0.933 |
| KB742630.1 | MAP3K8 | 60001 | 100000 | 0.208 | 0.914 |
| KB742648.1 | IQUB/NDUFA5/ASB15 | 160001 | 200000 | 0.208 | 1.751 |
| KB743013.1 | SH3RF1/CBR4 | 200001 | 240000 | 0.208 | 1.067 |
| KB743139.1 | SLC7A9/ENSAPLT00000006564 | 560001 | 600000 | 0.208 | 2.166 |
| KB742522.1 | SKI | 1420001 | 1460000 | 0.208 | 0.989 |
| KB743013.1 | PALLD/CBR4 | 220001 | 260000 | 0.207 | 0.995 |
| KB744266.1 | KIF17/DDOST/HP1BP3/SH2D5 | 260001 | 300000 | 0.207 | 0.998 |
| KB745546.1 | BRE | 100001 | 140000 | 0.207 | 1.455 |
| KB742877.1 | ELOVL2 | 500001 | 540000 | 0.207 | 0.980 |
| KB742685.1 | ENSAPLT00000016920 | 1800001 | 1840000 | 0.207 | 2.647 |
| KB742444.1 | SRBD1 | 200001 | 240000 | 0.207 | 1.528 |
| KB747340.1 | MYH15 | 1 | 40000 | 0.206 | 1.017 |
| KB743197.1 | EVL | 1780001 | 1820000 | 0.206 | 1.735 |
| KB743534.1 | GRM8 | 60001 | 100000 | 0.206 | 1.948 |
| KB742647.1 | IMMP2L | 420001 | 460000 | 0.206 | 0.964 |
| KB742619.1 | CEP135/EXOC1 | 1000001 | 1040000 | 0.205 | 1.749 |
| KB743108.1 | ENSAPLT00000008686 | 680001 | 720000 | 0.205 | 1.558 |
| KB742471.1 | PRKAR2B | 660001 | 700000 | 0.205 | 1.425 |
| KB743139.1 | CMTM4/DYNC1LI2 | 2100001 | 2140000 | 0.205 | 1.040 |
| KB742887.1 | PLCZ1/CAPZA3 | 1520001 | 1560000 | 0.205 | 1.070 |
| KB744077.1 | PDE8A | 360001 | 400000 | 0.205 | 1.561 |
| KB744033.1 | DEPDC5 | 1040001 | 1080000 | 0.204 | 1.068 |
| KB743073.1 | PAPPA2 | 980001 | 1020000 | 0.204 | 1.617 |
| KB742815.1 | UTRN | 220001 | 260000 | 0.204 | 1.592 |
| KB744601.1 | ENSAPLT00000016901 | 120001 | 160000 | 0.204 | 0.973 |
| KB743364.1 | KDM3B/ENSAPLT00000013236 | 1180001 | 1220000 | 0.204 | 1.407 |
| KB743153.1 | CCSA/NUP133/ENSAPLT00000004261 | 1540001 | 1580000 | 0.204 | 0.946 |
| KB742878.1 | TAPT1 | 780001 | 820000 | 0.204 | 1.015 |
| KB742471.1 | HBP1/PRKAR2B | 680001 | 720000 | 0.204 | 1.264 |
| KB743364.1 | ENSAPLT00000009716 | 780001 | 820000 | 0.204 | 0.863 |
| KB743291.1 | ERBB4 | 1280001 | 1320000 | 0.203 | 1.118 |
| KB744148.1 | ELAVL4 | 280001 | 320000 | 0.203 | 0.886 |
| KB743950.1 | CDH19 | 180001 | 220000 | 0.202 | 1.433 |
| KB742479.1 | DOK5 | 3380001 | 3420000 | 0.202 | 0.884 |
| KB743610.1 | MAPKAP1 | 380001 | 420000 | 0.202 | 1.652 |
| KB742685.1 | ENSAPLT00000016920 | 1740001 | 1780000 | 0.202 | 2.150 |
| KB744295.1 | VEGFC | 220001 | 260000 | 0.202 | 1.018 |
| KB742815.1 | PARK2 | 1300001 | 1340000 | 0.202 | 1.109 |
| KB742811.1 | ENSAPLT00000013062/MTMR4 | 760001 | 800000 | 0.202 | 0.899 |
| KB743070.1 | MPC1 | 220001 | 260000 | 0.202 | 1.011 |
| KB744094.1 | GAS2 | 100001 | 140000 | 0.201 | 1.742 |
| KB742523.1 | MEF2A | 640001 | 680000 | 0.201 | 0.996 |
| KB742432.1 | AKAP7 | 840001 | 880000 | 0.201 | 1.029 |
| KB743625.1 | NUP205/UTP20/ARL1 | 1300001 | 1340000 | 0.200 | 1.231 |
| KB743751.1 | PPP2R3B | 440001 | 480000 | 0.200 | 0.934 |
| KB744486.1 | TTC8 | 240001 | 280000 | 0.200 | 1.000 |
| KB743614.1 | AGA/ENSAPLT00000013850 | 1 | 40000 | 0.200 | 2.273 |
| KB743534.1 | GRM8 | 260001 | 300000 | 0.200 | 0.865 |
| KB742671.1 | ZNF518A | 1560001 | 1600000 | 0.199 | 0.962 |
| KB743364.1 | TCERG1 | 800001 | 840000 | 0.199 | 0.963 |
| KB742877.1 | SYCP2L/ELOVL2 | 540001 | 580000 | 0.199 | 1.081 |
| KB742464.1 | RNF24 | 2020001 | 2060000 | 0.199 | 0.877 |
| KB743057.1 | EIF2S2 | 620001 | 660000 | 0.198 | 0.868 |
| KB743430.1 | CCDC102B | 60001 | 100000 | 0.198 | 0.938 |
| KB742471.1 | PIK3CG | 560001 | 600000 | 0.198 | 1.205 |
| KB743162.1 | ENSAPLT00000002317 | 140001 | 180000 | 0.198 | 0.978 |
| KB742951.1 | BACH1 | 640001 | 680000 | 0.197 | 0.990 |
| KB742523.1 | CHD2 | 3260001 | 3300000 | 0.197 | 1.144 |
| KB743545.1 | TTC27 | 360001 | 400000 | 0.197 | 0.860 |
| KB742923.1 | POMP/SLC46A3 | 400001 | 440000 | 0.197 | 0.943 |
| KB743226.1 | CACUL1 | 420001 | 460000 | 0.197 | 1.029 |
| KB742406.1 | TINAG | 500001 | 540000 | 0.197 | 0.875 |
| KB743506.1 | ENSAPLT00000010340 | 20001 | 60000 | 0.197 | 1.463 |
| KB744198.1 | EMP2 | 540001 | 580000 | 0.197 | 1.000 |
| KB742537.1 | PAMR1 | 460001 | 500000 | 0.196 | 0.898 |
| KB742714.1 | GPAM | 1520001 | 1560000 | 0.196 | 1.142 |
| KB742405.1 | DPY19L4 | 40001 | 80000 | 0.196 | 0.914 |
| KB743197.1 | ENSAPLT00000000731/ENSAPLT00000000730 | 880001 | 920000 | 0.196 | 1.889 |
| KB742957.1 | ITGA8 | 120001 | 160000 | 0.196 | 0.970 |
| KB743336.1 | CRIM1 | 340001 | 380000 | 0.196 | 1.017 |
| KB742617.1 | C12orf4/RAD51AP1 | 1500001 | 1540000 | 0.196 | 1.365 |
| KB743073.1 | TNR | 1200001 | 1240000 | 0.196 | 1.166 |
| KB744638.1 | C1orf21 | 100001 | 140000 | 0.195 | 1.151 |
| KB742833.1 | AFAP1 | 5120001 | 5160000 | 0.195 | 0.941 |
| KB747003.1 | TNFRSF21 | 40001 | 80000 | 0.195 | 0.917 |
| KB743162.1 | NEDD1/ENSAPLT00000002023/ENSAPLT00000002191 | 60001 | 100000 | 0.195 | 0.896 |
| KB745378.1 | ENSAPLT00000016688 | 200001 | 240000 | 0.195 | 1.087 |
| KB743459.1 | THOC7 | 420001 | 460000 | 0.195 | 0.857 |
| KB743670.1 | PDE10A | 300001 | 340000 | 0.194 | 0.891 |
| KB744094.1 | GAS2 | 80001 | 120000 | 0.194 | 1.652 |
| KB743013.1 | PALLD | 280001 | 320000 | 0.194 | 1.108 |
| KB742923.1 | POMP/SLC46A3 | 380001 | 420000 | 0.194 | 0.942 |
| KB743810.1 | ELMO2/DDX27/ZNFX1/ENSAPLT00000012363 | 100001 | 140000 | 0.194 | 0.945 |
| KB742408.1 | KDM4A/ENSAPLT00000004338 | 240001 | 280000 | 0.194 | 1.132 |
| KB744976.1 | LCMT2 | 160001 | 200000 | 0.194 | 1.363 |
| KB742815.1 | FNDC1 | 560001 | 600000 | 0.194 | 0.997 |
| KB742773.1 | FERD3L | 3640001 | 3680000 | 0.194 | 1.157 |
| KB742675.1 | RELN | 80001 | 120000 | 0.194 | 1.226 |
| KB742685.1 | STON2 | 2960001 | 3000000 | 0.194 | 1.475 |
| KB743183.1 | PRKD1 | 920001 | 960000 | 0.193 | 1.142 |
| KB743534.1 | GRM8 | 340001 | 380000 | 0.193 | 2.051 |
| KB744033.1 | DEPDC5/ENSAPLT00000007561/ENSAPLT00000006670 | 1020001 | 1060000 | 0.193 | 1.034 |
| KB742739.1 | ABCC4 | 120001 | 160000 | 0.193 | 1.732 |
| KB742701.1 | CTPS2 | 1320001 | 1360000 | 0.193 | 1.009 |
| KB744937.1 | PSKH1/CTRL | 120001 | 160000 | 0.193 | 0.956 |
| KB742677.1 | CSRP1 | 1340001 | 1380000 | 0.193 | 0.999 |
| KB743545.1 | TTC27 | 340001 | 380000 | 0.193 | 0.863 |
| KB742563.1 | TRIM33 | 260001 | 300000 | 0.193 | 1.099 |
| KB743625.1 | CNOT4 | 1400001 | 1440000 | 0.193 | 0.949 |
| KB742815.1 | UTRN | 160001 | 200000 | 0.193 | 1.269 |
| KB742647.1 | IMMP2L | 360001 | 400000 | 0.192 | 1.204 |
| KB742969.1 | TMEM132D | 1100001 | 1140000 | 0.192 | 1.122 |
| KB744113.1 | CFAP161 | 620001 | 660000 | 0.192 | 1.432 |
| KB743197.1 | RCOR1 | 360001 | 400000 | 0.191 | 1.180 |
| KB744546.1 | ADAR/UBE2Q1/CHRNB2 | 1 | 40000 | 0.191 | 1.070 |
| KB742969.1 | ENSAPLT00000001260 | 520001 | 560000 | 0.191 | 1.256 |
| KB743116.1 | ANKRD28 | 40001 | 80000 | 0.191 | 1.075 |
| KB743818.1 | ZDHHC14 | 1 | 40000 | 0.191 | 1.291 |
| KB742833.1 | FGFRL1 | 400001 | 440000 | 0.191 | 0.927 |
| KB743670.1 | QKI | 1260001 | 1300000 | 0.191 | 1.326 |
| KB742567.1 | ENSAPLT00000009173 | 400001 | 440000 | 0.191 | 1.106 |
| KB742685.1 | ENSAPLT00000016920 | 1680001 | 1720000 | 0.191 | 1.344 |
| KB743335.1 | ERCC6 | 320001 | 360000 | 0.191 | 1.280 |
| KB742685.1 | ENSAPLT00000016920 | 1560001 | 1600000 | 0.191 | 1.357 |
| KB746044.1 | MTMR9 | 20001 | 60000 | 0.190 | 1.418 |
| KB742432.1 | ENPP3 | 580001 | 620000 | 0.190 | 1.031 |
| KB746044.1 | MTMR9/ENSAPLT00000015329 | 40001 | 80000 | 0.190 | 1.750 |
| KB742977.1 | ENSAPLT00000014574 | 100001 | 140000 | 0.190 | 0.972 |
| KB743546.1 | PREP | 240001 | 280000 | 0.190 | 0.858 |
| KB742408.1 | ENSAPLT00000004338 | 280001 | 320000 | 0.190 | 1.252 |
| KB742631.1 | GAD2 | 340001 | 380000 | 0.190 | 0.909 |
| KB742969.1 | RIMBP2/PIWIL1 | 1420001 | 1460000 | 0.190 | 0.901 |
| KB743506.1 | ENSAPLT00000010340 | 1 | 40000 | 0.189 | 1.197 |
| KB742878.1 | FBXL5/CC2D2A | 520001 | 560000 | 0.189 | 0.938 |
| KB743603.1 | GNB1L/ENSAPLT00000002306 | 280001 | 320000 | 0.188 | 1.479 |
| KB744477.1 | ENSAPLT00000004358 | 780001 | 820000 | 0.188 | 1.695 |
| KB742523.1 | ENSAPLT00000001352 | 700001 | 740000 | 0.188 | 0.911 |
| KB744198.1 | GRIN2A | 720001 | 760000 | 0.188 | 1.237 |
| KB742432.1 | TAAR/TAAR1 | 120001 | 160000 | 0.188 | 1.281 |
| KB742984.1 | WWOX | 1740001 | 1780000 | 0.188 | 0.853 |
| KB742675.1 | GSAP | 500001 | 540000 | 0.188 | 1.102 |
| KB744292.1 | ITGB1 | 900001 | 940000 | 0.188 | 2.105 |
| KB742619.1 | EXOC1/CEP135 | 980001 | 1020000 | 0.188 | 1.369 |
| KB743065.1 | ARPP21 | 300001 | 340000 | 0.188 | 1.075 |
| KB742685.1 | ENSAPLT00000016920 | 1820001 | 1860000 | 0.187 | 2.217 |
| KB744699.1 | ATCAY/ENSAPLT00000001423 | 440001 | 480000 | 0.187 | 1.028 |
| KB743402.1 | CCND1 | 780001 | 820000 | 0.187 | 1.989 |
| KB743111.1 | SPTLC3 | 960001 | 1000000 | 0.187 | 0.966 |
| KB742471.1 | PIK3CG | 540001 | 580000 | 0.187 | 1.346 |
| KB742862.1 | CHCHD2 | 740001 | 780000 | 0.187 | 1.771 |
| KB742386.1 | LATS1/NUP43 | 520001 | 560000 | 0.187 | 0.987 |
| KB743235.1 | EYA1 | 1300001 | 1340000 | 0.187 | 0.940 |
| KB743070.1 | MPC1 | 400001 | 440000 | 0.187 | 0.937 |
| KB742685.1 | ENSAPLT00000016920 | 1660001 | 1700000 | 0.187 | 1.345 |
| KB742523.1 | MCTP2 | 2780001 | 2820000 | 0.187 | 1.053 |
| KB742815.1 | PARK2 | 1440001 | 1480000 | 0.187 | 1.633 |
| KB742957.1 | ITGA8 | 140001 | 180000 | 0.186 | 0.912 |

Supplementary Table S9. The KEGG pathway of the loci under selections in domestic ducks (Top 20)

| NO. | Pathway | Gene number | Pvalue |
| --- | --- | --- | --- |
| 1 | [Pantothenate and CoA biosynthesis](file:///C:\\Users\\soi\\AppData\\Local\\Temp\\360zip$Temp\\360$0\\pathwaygseaf1e1b2\\out\\201525970043.os\\KO\\out.htm#gene1) | 2 | 0.026677 |
| 2 | [FoxO signaling pathway](file:///C:\\Users\\soi\\AppData\\Local\\Temp\\360zip$Temp\\360$0\\pathwaygseaf1e1b2\\out\\201525970043.os\\KO\\out.htm#gene2) | 6 | 0.030017 |
| 3 | [Inositol phosphate metabolism](file:///C:\\Users\\soi\\AppData\\Local\\Temp\\360zip$Temp\\360$0\\pathwaygseaf1e1b2\\out\\201525970043.os\\KO\\out.htm#gene3) | 4 | 0.035111 |
| 4 | [Phosphatidylinositol signaling system](file:///C:\\Users\\soi\\AppData\\Local\\Temp\\360zip$Temp\\360$0\\pathwaygseaf1e1b2\\out\\201525970043.os\\KO\\out.htm#gene4) | 4 | 0.076842 |
| 5 | [VEGF signaling pathway](file:///C:\\Users\\soi\\AppData\\Local\\Temp\\360zip$Temp\\360$0\\pathwaygseaf1e1b2\\out\\201525970043.os\\KO\\out.htm#gene5) | 3 | 0.081043 |
| 6 | [Nicotinate and nicotinamide metabolism](file:///C:\\Users\\soi\\AppData\\Local\\Temp\\360zip$Temp\\360$0\\pathwaygseaf1e1b2\\out\\201525970043.os\\KO\\out.htm#gene6) | 2 | 0.086185 |
| 7 | [Fructose and mannose metabolism](file:///C:\\Users\\soi\\AppData\\Local\\Temp\\360zip$Temp\\360$0\\pathwaygseaf1e1b2\\out\\201525970043.os\\KO\\out.htm#gene7) | 2 | 0.086185 |
| 8 | [ECM-receptor interaction](file:///C:\\Users\\soi\\AppData\\Local\\Temp\\360zip$Temp\\360$0\\pathwaygseaf1e1b2\\out\\201525970043.os\\KO\\out.htm#gene8) | 3 | 0.093906 |
| 9 | [Focal adhesion](file:///C:\\Users\\soi\\AppData\\Local\\Temp\\360zip$Temp\\360$0\\pathwaygseaf1e1b2\\out\\201525970043.os\\KO\\out.htm#gene9) | 6 | 0.110934 |
| 10 | [ErbB signaling pathway](file:///C:\\Users\\soi\\AppData\\Local\\Temp\\360zip$Temp\\360$0\\pathwaygseaf1e1b2\\out\\201525970043.os\\KO\\out.htm#gene10) | 3 | 0.114655 |
| 11 | [Starch and sucrose metabolism](file:///C:\\Users\\soi\\AppData\\Local\\Temp\\360zip$Temp\\360$0\\pathwaygseaf1e1b2\\out\\201525970043.os\\KO\\out.htm#gene11) | 2 | 0.117913 |
| 12 | [RNA transport](file:///C:\\Users\\soi\\AppData\\Local\\Temp\\360zip$Temp\\360$0\\pathwaygseaf1e1b2\\out\\201525970043.os\\KO\\out.htm#gene12) | 5 | 0.123180 |
| 13 | [Jak-STAT signaling pathway](file:///C:\\Users\\soi\\AppData\\Local\\Temp\\360zip$Temp\\360$0\\pathwaygseaf1e1b2\\out\\201525970043.os\\KO\\out.htm#gene13) | 4 | 0.123567 |
| 14 | [Regulation of actin cytoskeleton](file:///C:\\Users\\soi\\AppData\\Local\\Temp\\360zip$Temp\\360$0\\pathwaygseaf1e1b2\\out\\201525970043.os\\KO\\out.htm#gene14) | 6 | 0.131763 |
| 15 | [Proteasome](file:///C:\\Users\\soi\\AppData\\Local\\Temp\\360zip$Temp\\360$0\\pathwaygseaf1e1b2\\out\\201525970043.os\\KO\\out.htm#gene15) | 2 | 0.140443 |
| 16 | [ABC transporters](file:///C:\\Users\\soi\\AppData\\Local\\Temp\\360zip$Temp\\360$0\\pathwaygseaf1e1b2\\out\\201525970043.os\\KO\\out.htm#gene16) | 2 | 0.140443 |
| 17 | [Insulin signaling pathway](file:///C:\\Users\\soi\\AppData\\Local\\Temp\\360zip$Temp\\360$0\\pathwaygseaf1e1b2\\out\\201525970043.os\\KO\\out.htm#gene17) | 4 | 0.153747 |
| 18 | [RNA degradation](file:///C:\\Users\\soi\\AppData\\Local\\Temp\\360zip$Temp\\360$0\\pathwaygseaf1e1b2\\out\\201525970043.os\\KO\\out.htm#gene18) | 3 | 0.168808 |
| 19 | [Glycerophospholipid metabolism](file:///C:\\Users\\soi\\AppData\\Local\\Temp\\360zip$Temp\\360$0\\pathwaygseaf1e1b2\\out\\201525970043.os\\KO\\out.htm#gene19) | 3 | 0.168808 |
| 20 | [Neuroactive ligand-receptor interaction](file:///C:\\Users\\soi\\AppData\\Local\\Temp\\360zip$Temp\\360$0\\pathwaygseaf1e1b2\\out\\201525970043.os\\KO\\out.htm#gene20) | 8 | 0.180605 |

Supplementary Table S10 The GO classification of the loci under selections in domestic ducks

| NO. | Ontology | Class | Gene number |
| --- | --- | --- | --- |
| 1 | Biological Process | reproduction | 12 |
| 2 | Biological Process | cell killing | 2 |
| 3 | Biological Process | immune system process | 13 |
| 4 | Biological Process | behavior | 5 |
| 5 | Biological Process | metabolic process | 77 |
| 6 | Biological Process | cellular process | 137 |
| 7 | Biological Process | reproductive process | 12 |
| 8 | Biological Process | biological adhesion | 16 |
| 9 | Biological Process | signaling | 40 |
| 10 | Biological Process | multicellular organismal process | 56 |
| 11 | Biological Process | developmental process | 54 |
| 12 | Biological Process | growth | 2 |
| 13 | Biological Process | locomotion | 15 |
| 14 | Biological Process | single-organism process | 123 |
| 15 | Biological Process | rhythmic process | 3 |
| 16 | Biological Process | response to stimulus | 64 |
| 17 | Biological Process | localization | 46 |
| 18 | Biological Process | multi-organism process | 14 |
| 19 | Biological Process | biological regulation | 92 |
| 20 | Biological Process | cellular component organization or biogenesis | 47 |
| 21 | Biological Process | cell aggregation | 2 |
| 22 | Molecular Function | transcription factor activity, protein binding | 3 |
| 23 | Molecular Function | nucleic acid binding transcription factor activity | 9 |
| 24 | Molecular Function | catalytic activity | 50 |
| 25 | Molecular Function | signal transducer activity | 17 |
| 26 | Molecular Function | structural molecule activity | 6 |
| 27 | Molecular Function | transporter activity | 12 |
| 28 | Molecular Function | binding | 107 |
| 29 | Molecular Function | chemoattractant activity | 1 |
| 30 | Molecular Function | molecular transducer activity | 15 |
| 31 | Molecular Function | molecular function regulator | 6 |
| 32 | Cellular Component | extracellular region | 14 |
| 33 | Cellular Component | cell | 149 |
| 34 | Cellular Component | membrane | 85 |
| 35 | Cellular Component | cell junction | 6 |
| 36 | Cellular Component | extracellular matrix | 9 |
| 37 | Cellular Component | membrane-enclosed lumen | 37 |
| 38 | Cellular Component | macromolecular complex | 52 |
| 39 | Cellular Component | organelle | 111 |
| 40 | Cellular Component | extracellular matrix component | 4 |
| 41 | Cellular Component | extracellular region part | 10 |
| 42 | Cellular Component | organelle part | 75 |
| 43 | Cellular Component | membrane part | 70 |
| 44 | Cellular Component | synapse part | 9 |
| 45 | Cellular Component | cell part | 149 |
| 46 | Cellular Component | synapse | 12 |
| 47 | Cellular Component | supramolecular fiber | 2 |

Supplementary Table S11 Summarize of sequence mapping of three tissues in Shaoxing ducks and mallards

|  | MA_Muscle | SX_Muscle | MA_Liver | SX_Liver | MA_Cb | SX_Cb |
| --- | --- | --- | --- | --- | --- | --- |
| Total Reads | 41149861 | 40551060 | 40465345 | 40394843 | 40550410 | 40799808 |
| Mapped Reads | 21742848 | 24305767 | 21939527 | 22676133 | 28458608 | 28632319 |
| Mapping Rate | 0.5286 | 0.5994 | 0.5427 | 0.5612 | 0.7019 | 0.7016 |
| Unmapped Reads | 19407013 | 16245293 | 18525818 | 17718709 | 12091802 | 12167489 |
| Multi Map Reads | 337963 | 394682 | 375501 | 364502 | 183888 | 183684 |
| Multi Map Rate | 0.0082 | 0.0098 | 0.0093 | 0.009 | 0.0045 | 0.0045 |

Supplementary Table S12 Pathway of KEGG differentially expressed gene in muscle

| Description | Gene DE | q value | Rich Ratio |
| --- | --- | --- | --- |
| Parkinson disease | 19 | 3.78E-11 | 8.62 |
| Oxidative phosphorylation | 18 | 4.62E-10 | 7.88 |
| Huntington disease | 20 | 8.99E-09 | 5.68 |
| Alzheimer disease | 19 | 8.99E-09 | 5.99 |
| Fatty acid degradation | 6 | 6.09E-03 | 7.20 |
| Cardiac muscle contraction | 6 | 2.56E-02 | 5.44 |
| Valine, leucine and isoleucine degradation | 6 | 2.56E-02 | 5.31 |

Supplementary Table S13 The down-regulated genes in muscle of Shaoxing ducks (top 20)

| Gene Name | Description | p |
| --- | --- | --- |
| ENSAPLG00000008730 | homeodomain-only protein isoform X2 | 1.250E-20 |
| SCNN1A | amiloride-sensitive sodium channel subunit alpha | 3.060E-17 |
| TIPARP | TCDD-inducible poly [ADP-ribose] polymerase, partial | 4.770E-15 |
| ENSAPLG00000000981 | hypothetical protein Anapl_17174, partial | 6.300E-12 |
| HYAL1 | Hyaluronidase-1, partial | 1.370E-09 |
| ENSAPLG00000009268 | Syndecan-3, partial | 6.550E-07 |
| NT5DC1 | 5'-nucleotidase domain-containing protein 1 | 1.640E-06 |
| MTMR7 | Myotubularin-related protein 7, partial | 6.360E-06 |
| MADD | MAP kinase-activating death domain protein isoform X19 [Haplochromis burtoni] | 7.780E-06 |
| ENSAPLG00000015931 | lpha-aspartyl dipeptidase-like | 7.780E-06 |
| TRIB2 | tribbles homolog 2 | 7.840E-06 |
| ENSAPLG00000009305 | maltase-glucoamylase, intestinal-like isoform X1 | 1.760E-05 |
| ENSAPLG00000000897 | hypothetical protein N301_14584, partial | 1.840E-05 |
| SESN1 | sestrin-1 isoform X2 | 1.860E-05 |
| ENSAPLG00000003760 | teneurin-2 isoform X2 | 1.910E-05 |
| ABCC5 | Multidrug resistance-associated protein 5, partial | 2.200E-05 |
| F2R | proteinase-activated receptor 1 | 2.240E-05 |
| AP3B2 | AP-3 complex subunit beta-2 | 2.320E-05 |
| F2RL1 | Proteinase-activated receptor 2, partial | 2.720E-05 |
| HHIPL2 | LOW QUALITY PROTEIN: HHIP-like protein 2 | 3.000E-05 |

Supplementary Table S14 The up-regulated genes in muscle of Shaoxing ducks (top 20)

| Gene Name | Description | p |
| --- | --- | --- |
| ENAH | protein enabled homolog isoform X5 | 7.440E-13 |
| ENSAPLG00000003647 | class I histocompatibility antigen, F10 alpha chain-like | 9.640E-11 |
| ENSAPLG00000005009 | class II histocompatibility antigen, B-L beta chain | 3.590E-10 |
| SGMS2 | phosphatidylcholine:ceramide cholinephosphotransferase 2 | 2.230E-09 |
| ENSAPLG00000004095 | musculoskeletal embryonic nuclear protein 1 | 5.840E-09 |
| ENSAPLG00000006375 | LOW QUALITY PROTEIN: tumor necrosis factor receptor superfamily member 27 | 2.300E-08 |
| ENSAPLG00000015472 | LOW QUALITY PROTEIN: IgGFc-binding protein-like, partial | 1.160E-07 |
| KIAA1161 | uncharacterized family 31 glucosidase KIAA1161 homolog | 1.510E-07 |
| ENSAPLG00000009683 | dynein heavy chain 5, axonemal-like | 9.480E-07 |
| PKIA | cAMP-dependent protein kinase inhibitor alpha | 1.020E-06 |
| KCNT1 | potassium channel subfamily T member 1 | 1.190E-06 |
| ENSAPLG00000016379 | insulin-like growth factor-binding protein 3 | 1.200E-06 |
| PRSS35 | inactive serine protease 35 | 1.400E-06 |
| ENSAPLG00000002180 | frizzled-2 | 1.640E-06 |
| RPL22L1 | 60S ribosomal protein L22-like 1 | 1.740E-06 |
| TOM1 | Target of Myb protein 1, partial | 1.820E-06 |
| ENSAPLG00000004635 | GATS-like protein 2 | 2.140E-06 |
| BCAR3 | breast cancer anti-estrogen resistance protein 3 isoform X3 | 2.860E-06 |
| FGGY | FGGY carbohydrate kinase domain-containing protein isoform X3 | 3.410E-06 |
| PCMTD2 | protein-L-isoaspartate O-methyltransferase domain-containing protein 2 | 4.830E-06 |

Supplementary Table S15 The down-regulated genes in liver of Shaoxing ducks (top 20)

| Gene Name | Description | p |
| --- | --- | --- |
| ENSAPLG00000001196 | fatty acid-binding protein, liver-like | 6.260E-23 |
| ENSAPLG00000002361 | interferon-induced very large GTPase 1-like, partial | 3.790E-22 |
| ANXA10 | annexin A10 isoform X1 | 1.740E-18 |
| ENSAPLG00000000936 | type II iodothyronine deiodinase isoform X1 | 7.800E-12 |
| Metazoa_SRP | uncharacterized protein LOC104057446 | 2.340E-11 |
| ENSAPLG00000001071 | LOW QUALITY PROTEIN: interferon-induced very large GTPase 1-like [Anser cygnoides domesticus] | 8.900E-11 |
| NUDT21 | cleavage and polyadenylation specificity factor subunit 5 isoform X1 | 8.900E-11 |
| TNFRSF6B | tumor necrosis factor receptor superfamily member 6B | 2.850E-09 |
| TRPM1 | Transient receptor potential cation channel subfamily M member 1, partial | 4.910E-09 |
| ASNS | asparagine synthetase | 1.590E-08 |
| CHKA | choline kinase alpha isoform X3 | 1.860E-08 |
| PLIN2 | perilipin 2 | 3.530E-07 |
| CADM2 | cell adhesion molecule 2 isoform X1 | 2.540E-06 |
| SLC16A5 | monocarboxylate transporter 6 isoform X1 | 6.430E-06 |
| PAPLN | papilin isoform X4 | 6.430E-06 |
| ENSAPLG00000013309 | histone acetyltransferase KAT2A, partial | 2.130E-05 |
| ENSAPLG00000006863 | Calretinin, partial [Tauraco erythrolophus] | 2.770E-05 |
| KCTD16 | LOW QUALITY PROTEIN: BTB/POZ domain-containing protein KCTD16 | 2.770E-05 |
| AVPR1A | vasopressin V1a receptor | 2.860E-05 |
| ABL2 | Tyrosine-protein kinase ABL2, partial | 5.520E-05 |

Supplementary Table S16 The up-regulated genes in liver of Shaoxing ducks (top 20)

| Gene Name | Description | p |
| --- | --- | --- |
| ENSAPLG00000001080 | Serum amyloid P-component, partial | 1.570E-22 |
| ENSAPLG00000006066 | disintegrin and metalloproteinase domain-containing protein 2-like, partial | 7.650E-20 |
| ENSAPLG00000000546 | miRAN | 6.640E-12 |
| ENSAPLG00000014731 | uncharacterized protein LOC106046799 | 3.980E-10 |
| TRPA1 | transient receptor potential cation channel subfamily A member 1 | 4.740E-08 |
| BST1 | ADP-ribosyl cyclase/cyclic ADP-ribose hydrolase 2 | 9.420E-06 |
| ENSAPLG00000005009 | class II histocompatibility antigen, B-L beta chain | 2.770E-05 |
| ABLIM3 | actin-binding LIM protein 3 isoform X1 | 5.410E-05 |
| ARHGEF37 | rho guanine nucleotide exchange factor 37 isoform X1 | 5.940E-05 |
| GALNT14 | polypeptide N-acetylgalactosaminyltransferase 14 | 6.870E-05 |
| ENSAPLG00000000754 | type III iodothyronine deiodinase | 1.105E-04 |
| ENSAPLG00000003647 | class I histocompatibility antigen, F10 alpha chain-like | 1.256E-04 |
| RPS6KL1 | LOW QUALITY PROTEIN: ribosomal protein S6 kinase-like 1, partial | 1.378E-04 |
| TP53I3 | quinone oxidoreductase PIG3 | 1.470E-04 |
| ENSAPLG00000001594 | myelin-oligodendrocyte glycoprotein, partial | 2.343E-04 |
| ARHGEF16 | rho guanine nucleotide exchange factor 16, partial | 3.278E-04 |
| ENSAPLG00000009649 | Butyrophilin subfamily 3 member A3, partial | 3.659E-04 |
| HSD17B2 | estradiol 17-beta-dehydrogenase 2 | 4.064E-04 |
| SIDT1 | SID1 transmembrane family member 1 isoform X1 | 7.092E-04 |
| KLHL23 | kelch-like protein 23, partial | 1.588E-03 |

Supplementary Table S17 The down-regulated genes in liver of Shaoxing ducks

| Gene Name | Description | p |
| --- | --- | --- |
| PENK | proenkephalin (PENK), mRNA | 3.120E-07 |
| ENSAPLG00000000981 | cytochrome b-c1 complex subunit 10 (LOC101799882), mRNA | 1.320E-05 |
| ENSAPLG00000010845 | domesticus mucin-5B (LOC106042636), mRNA | 1.374E-03 |
| SNORA27 | Apteryx australis mantelli genome assembly AptMant0, scaffold scaffold132 | 2.195E-03 |
| CPNE7 | copine VII (CPNE7), mRNA | 4.914E-03 |
| ENSAPLG00000008730 | HOP homeobox (HOPX), transcript variant X6, mRNA | 9.002E-03 |
| ENSAPLG00000012381 | domesticus angiopoietin-related protein 7-like (LOC106038099), mRNA | 1.323E-02 |
| ENSAPLG00000000897 | ribosomal protein S28 (RPS28), partial mRNA | 1.376E-02 |
| ENSAPLG00000014124 | extracellular serine/threonine protein kinase FAM20C-like (LOC101796295), mRNA | 2.376E-02 |

Supplementary Table S18 The up-regulated genes in liver of Shaoxing ducks

| Gene Name | Description | p |
| --- | --- | --- |
| EVPL | envoplakin (EVPL), transcript variant X4, mRNA | 7.950E-08 |
| ADGRD2 | adhesion G protein-coupled receptor D2 (ADGRD2), mRNA | 3.970E-05 |
| ENSAPLG00000008949 | keratin, type I cytoskeletal 15 (LOC101792676), mRNA | 7.550E-05 |
| TNFSF8 | tumor necrosis factor (ligand) superfamily, member 8 (TNFSF8), mRNA | 1.577E-04 |
| ENSAPLG00000014299 | zinc finger protein 335 (ZNF335), mRNA | 2.195E-03 |
| ENSAPLG00000010262 | polyamine-modulated factor 1 (PMF1), mRNA | 3.377E-03 |
| ENSAPLG00000003756 | protein O-GlcNAcase-like (LOC101803302), partial mRNA | 6.909E-03 |
| ENSAPLG00000002689 | steroid receptor RNA activator 1 (SRA1), partial mRNA | 7.133E-03 |
| FOS | FBJ murine osteosarcoma viral oncogene homolog (FOS), partial mRNA | 7.540E-03 |
| ENSAPLG00000003647 | class I histocompatibility antigen, F10 alpha chain-like (LOC101794830), mRNA | 8.124E-03 |
| TRAPPC4 | trafficking protein particle complex 4 (TRAPPC4), partial mRNA | 1.433E-02 |
| DUSP1 | dual specificity phosphatase 1 (DUSP1), partial mRNA | 1.484E-02 |
| PHYHD1 | phytanoyl-CoA dioxygenase domain containing 1 (PHYHD1), mRNA | 1.484E-02 |
| NECAB1 | N-terminal EF-hand calcium binding protein 1 (NECAB1), partial mRNA | 2.721E-02 |
| PARP3 | poly (ADP-ribose) polymerase family, member 3 (PARP3), partial mRNA | 3.050E-02 |
| SCNN1B | sodium channel, non voltage gated 1 beta subunit (SCNN1B), mRNA | 3.159E-02 |
| SMIM8 | small integral membrane protein 8 (SMIM8), mRNA | 3.710E-02 |
| TMEM116 | transmembrane protein 116 (TMEM116), transcript variant X1, mRNA | 4.893E-02 |
